# Genome-Wide Analysis of Callose Synthase (CALS) Genes in Cabbage (*Brassica oleracea* var. *capitata* L.): Identification and Expression Profiling During *Hyaloperonospora parasitica* Infection

**DOI:** 10.3390/ijms262110304

**Published:** 2025-10-23

**Authors:** Jiamin Li, Yuankang Wu, Xuehui Yao, Limei Yang, Mu Zhuang, Honghao Lv, Yong Wang, Jialei Ji, Yangyong Zhang

**Affiliations:** 1 State Key Laboratory of Vegetable Biobreeding, Institute of Vegetables and Flowers, Chinese Academy of Agricultural Sciences, Beijing 100081, China; pyzx240ljm@163.com (J.L.); wuyk1129@163.com (Y.W.); yaoxuehui@caas.cn (X.Y.); yanglimei@caas.cn (L.Y.); zhuangmu@caas.cn (M.Z.); lvhonghao@caas.cn (H.L.); wangyong@caas.cn (Y.W.); jijialei@caas.cn (J.J.); 2 National Key Laboratory of Crop Genetics & Germplasm Innovation and Utilization, Key Laboratory of Biology and Genetic Improvement of Horticultural Crops (East China), Ministry of Agriculture and Rural Affairs of China, Engineering Research Center of Germplasm Enhancement and Utilization of Horticultural Crops, Ministry of Education of China, Nanjing Agricultural University, Nanjing 210095, China

**Keywords:** *Brassica oleracea*, callose synthase enzyme, downy mildew, gene family, expression analysis

## Abstract

Callose synthase (*CALS*) genes are known to play critical roles in microspore development and in plant responses to diverse biotic and abiotic stresses. While the role of *CALS* genes has been extensively characterized in several plant species, their homologs in *Brassica oleracea* (*BoCALS*) remain understudied. In this study, 15 *BoCALS* genes were identified in *B. oleracea* genome, distributed across eight chromosomes. All BoCALS proteins contain Glucan-synthase and Fks1 domains. Phylogenetic analysis grouped *BoCALS* and their homologs from *Arabidopsis thaliana* and *Brassica rapa* into three distinct Clusters (Ⅰ–Ⅲ), revealing conserved evolutionary relationships within the *Brassicaceae* family. Collinearity analysis showed that *AtCALS* genes of *Arabidopsis* have multiple orthologs in *B. oleracea*. Analysis of RNA-Seq data from public databases suggested that most of the *BoCALS* genes exhibit tissue-specific expression patterns, indicating their potential roles in organ differentiation and development. QRT-PCR analysis elucidated a different expression level of *BoCALS* genes in response to *Hyaloperonospora parasitica* infection. Notably, *BoCALS6* expression was significantly higher in resistant varieties compared to susceptible varieties and further up-regulated following *H. parasitica* infection, indicating its potential role in downy mildew resistance. This study presents the first comprehensive characterization of *BoCALS* gene family in *B. oleracea* and provides a foundation for further functional investigations into their roles in downy mildew resistance.

## 1. Introduction

Angiosperms are subjected to various biotic and abiotic stresses throughout their growth and development. To mitigate these challenges, plants have evolved sophisticated defense mechanisms, among which callose deposition plays a pivotal role [1]. Callose, a linear β-1,3-glucan polysaccharide with minor amounts of β-1,6-branching, occurs ubiquitously in the cell walls of higher plants [2]. Callose deposition is a crucial response that enhances cellular resilience against environmental stressors, including pathogen attack and physicochemical damage [3]. Functionally, callose acts as a physical barrier, preventing the penetration of cell wall-degrading enzymes secreted by pathogens into adjacent cells [4]. Upon the alleviation of stress, callose is rapidly degraded to restore normal sieve tube transport [5]. For instance, in *Arabidopsis*, PLASMODESMATA-LOCATED PROTEIN 1 (*PDLP1*) regulates callose accumulation around sucker-specific structures formed by downy mildew pathogens and fungi, thereby limiting pathogen spread [6]. Similarly, callose deposition at plasmodesmata (PD) during the defense against soybean mosaic virus infection restricts viral cell-to-cell movement in soybean, contributing to disease resistance [7].

Callose is synthesized by callose synthases (CALS), a class of enzymes that utilize UDP-glucose as a substrate [8]. The activity and expression levels of CALS enzymes directly regulate callose accumulation in plants [9]. In angiosperms, the *CALS* gene family is exceptionally large. Individual CALS proteins typically comprise approximately 2000 amino acid residues, with a molecular weight of around 200 kDa, ranking among the largest known plant proteins [9]. Genome-wide identification and characterization of the *CALS* gene family have been conducted in numerous plant species, including *Arabidopsis thaliana* (12 genes) [8,10], *Oryza sativa* (rice, 10 genes) [11], *Solanum lycopersicum* (tomato, 10–13 genes) [12], *Brassica napus* (12 genes) [13], *Brassica rapa* (Chinese cabbage, 15 genes) [14], *Cucumis sativus* (cucumber, 10 genes) [15], *Zea mays* (maize, 10 genes) [16] and *Sorghum bicolor* (sorghum, 11 genes) [17]. Notably, the *CALS* gene family is also designated as the Glucan Synthase-Like (*GSL*) gene family [14,18] or PD-located proteins (PDLPs) [19] in several studies.

The *CALS* gene family is involved in mediating higher plant resistance to adversity and stress, a function extensively documented across diverse species. In *A. thaliana*, *AtGSL5* (also known as *PMR4*, POWDERY MILDEW RESISTANT 4) is required for callose deposition in root hairs under inorganic phosphate (Pi) deficiency [20]. Transient expression of *AtGSL5* (*PMR4*) in barley leaves enhances penetration resistance against powdery mildew (*Blumeria graminis* f. sp. *Hordei*) [21]. Similarly, silencing *HvGSL6* via double-stranded RNA interference (dsRNAi) significantly compromised callose deposition at wound sites, leading to increased susceptibility to powdery mildew infection in barley. This finding further supports the essential role of *CALS* genes in mediating resistance to this pathogen [22]. In citrus, *CsCALS2*, *CsCALS7*, and *CsCALS12* have been implicated in defense against *Candidatus* Liberibacter asiaticus (CLas), the causal agent of Huanglongbing (HLB). Callose deposition in phloem sieve tubes restricted CLas colonization by impairing phloem transport, thereby suppressing disease progression [23]. In summary, *CALS* genes encode the essential enzymes responsible for callose synthesis in higher plants. This process effectively blocks the transportation of harmful substances through sieve pores and sieve tube surfaces, ensuring normal phloem function while providing a structural barrier against a range of environmental stresses.

Although the roles of *CALS* genes in plant growth, stress responses, and developmental processes have been well characterized, a comprehensive analysis of the *CALS* gene family in *B. oleracea* remains lacking, and its functional significance in this species remains poorly understood. Cabbage is one of the most widely cultivated vegetable crops globally. However, its production has been severely constrained by four major diseases: Fusarium wilt, clubroot, downy mildew and black rot [24]. Identifying defense-related genes is therefore essential for the development of resistant cultivars. Research on downy mildew resistance genes in cabbage, in particular, remains limited. Consequently, characterizing candidate genes from multiple perspectives and using integrative approaches is crucial for elucidating the molecular mechanisms underlying disease resistance and accelerating resistance breeding programs.

In this study, we identified 15 *BoCALS* genes in the *B. oleracea* genome based on sequence homology with *A. thaliana*. We further generated a tissue-specific expression heatmap using RNA-seq expression data to assess their transcriptional profile across various organs. Furthermore, comparative transcriptomic analysis revealed distinct expression patterns of *BoCALS* genes between *H. parasitica*-susceptible and *H. parasitica*-resistant cabbage lines following pathogen inoculation. These findings establish a foundation framework for further functional investigations of *BoCALS* genes and provide valuable insights into their potential role in conferring resistance to downy mildew in *B. oleracea*.

## 2. Results

### 2.1. Identification and Characterization of BoCALS Family Genes in B. oleracea

To identify the genes encoding callose synthase in *B. oleracea*, a BLASTP (2.16.0 version) search was performed against the *B. oleracea* genome using 12 AtCALS proteins as queries; a total of 19 candidate genes were initially identified. Pfam, InterPro and NCBI CDD search tools were used to analyze the results. Based on the presence and completeness of conserved domains and motifs, four genes lacking conserved CALS enzyme domain were excluded. Ultimately, 15 genes containing intact Glucan-synthase domain (PF02364) were considered as encoding callose synthases (Table 1). The full length of BoCALS proteins ranged from 1768 amino acids (*BoCALS14*) to 2312 amino acids (*BoCALS10*). The relative molecular weights (MW) of BoCALS varied from 204.9 kDa (*BoCALS14*) to 267.09 (*BoCALS10*) kDa, and the theoretical isoelectric points (pl) ranged from 8.41 (*BoCALS9*) to 9.21 (*BoCALS12*). GRAVY value showed that most of (13) BoCALS proteins were hydrophilic, whereas *BoCALS3*, *BoCALS4*, and *BoCALS14* exhibited hydrophobic properties. Subcellular localization predictions showed that all BoCALS proteins were most likely located in the plasma membrane.

### 2.2. Phylogenetic and Gene Structural Analysis of BoCALS Genes

**Figure 1:**
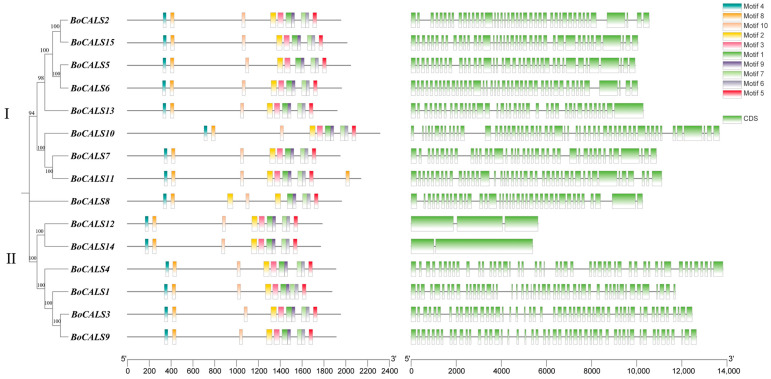
Phylogenetic tree and genetic structure of the *BoCALS* gene family in *B. oleracea.* A. Phylogenetic relationships of *BoCALS* genes in *B. oleracea*. B. Distribution of conserved motifs and domains of *B. oleracea* BoCALS proteins, A schematic of the ten conserved motifs and three conserved domains is shown in the upper right panel, with different motifs and domains distinguished by colors. C. Exon-intron distribution of the *BoCALS* genes, exons are indicated by green boxes and black lines represent introns.

To investigate the relationship between the classification and structure of *BoCALS* genes, we analyzed the gene structure, conserved domains and protein motifs of the BoCALS proteins. Initially, we constructed a phylogenetic tree of the 15 BoCALS proteins and grouped them into two clusters (Supplementary File S2) (Figure 1). All proteins were found to contain the 1,3-beta-glucan-synthase domain and the Fks1 domain [25], and all seven members in the Cluster Ⅰ also possessed the Vta1 domain [26]. The members share similar conserved motifs, specifically, motif 4 and motif 8 were located within the Fks1 domain, while motif 10, motif 2, motif 3, motif 1, motif 9, motif 7, motif 6, and motif 5 were distributed within the 1,3-beta-glucan-synthase domain (Supplementary Table S3). Notably, *BoCALS8* contained an additional motif 2 between its 1,3-beta-glucan-synthase and Fks1 domains. Overall, the high conservation of the Fks1 and the 1,3-beta-glucan-synthase domains provides strong evidence for functional consistency among *BoCALS* family members. Gene structure analysis revealed that most of *BoCALS* genes are exons rich. *BoCALS10* possessed the highest number of exons, with 53. In contrast, *BoCALS12* and *BoCALS14* contained only three and two exons, respectively, with gene lengths shorter than 6000 bp.

### 2.3. Chromosomal Localization and Collinearity Analysis of BoCALS Genes

**Figure 2:**
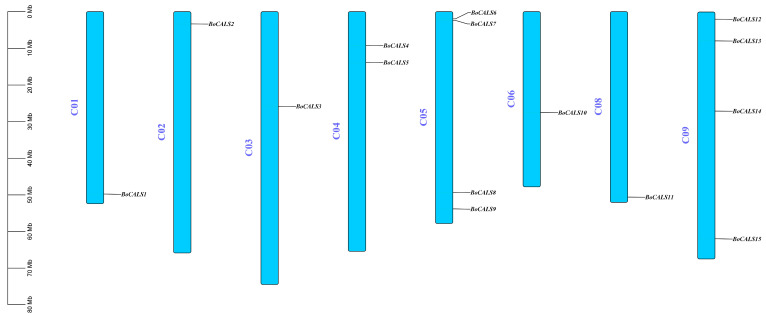
Chromosome localization of *BoCALS*. Eight columns represent eight chromosomes, the scale size of the chromosomes is labeled on the far left, and the location of the genes is shown on the right side of each chromosome.

15 *BoCALS* genes were mapped to eight chromosomes of *B. oleracea* (Figure 2). These genes are unevenly distributed across the *B. oleracea* chromosomes. Specifically, four genes (*BoCALS6*-*9*) are located on chromosome 5, and another four (*BoCALS12*-*15*) on chromosome 9. In contrast, only one *BoCALS* gene was identified on each of chromosomes 1, 2, 3, 6, and 8. Notably, we did not find the *BoCALS* gene on chromosome 7.

**Figure 3:**
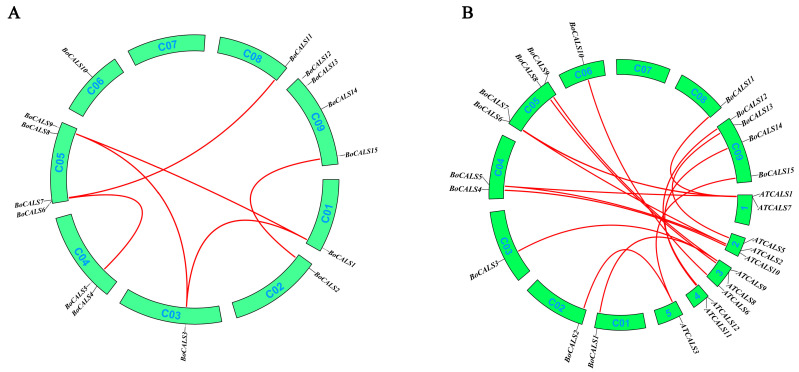
Collinearity analysis of *CALS* genes in *B. oleracea* and *B. oleracea* with *A. thaliana.* Red lines highlight the covariant gene pairs. (**A**) Collinearity analysis of *BoCALS* genes. (**B**) Collinearity analysis of *BoCALS* and *ATCALS* genes.

Subsequently, we investigated the duplication events of *CALS* genes between *B. oleracea* and *A. thaliana* to further understand the evolutionary dynamics of the *CALS* gene family (Figure 3). No tandem duplication events were identified within the *BoCALS* gene family; however, six gene pairs were found to have undergone segmental duplications. Among the 12 *AtCALS* genes, 11 exhibited at least one orthologous region in the *B. oleracea* genome. *AtCALS4* was the only gene without a corresponding in *B. oleracea*, suggesting potential gene loss or lineage-specific divergence.

### 2.4. Analysis of Phylogenetic Tree of CALS Genes Across B. oleracea, B. rapa and A. thaliana

**Figure 4:**
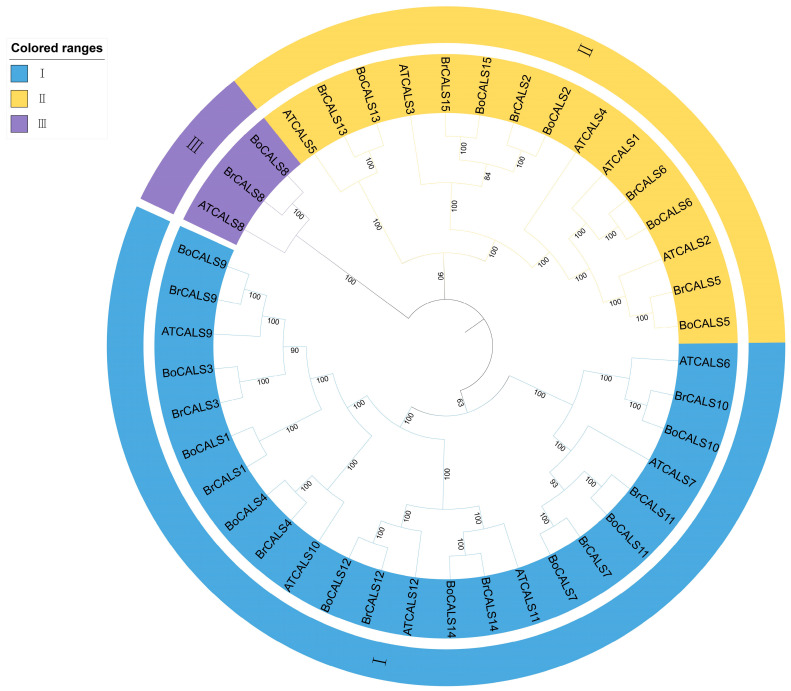
Phylogenetic relationships of CALS proteins from *B. oleracea*, *A. thaliana*, and *B. rapa*.

A phylogenetic tree was constructed based on the full-length protein sequences of *BoCALS*, *AtCALS* and *BrCALS* genes to explore genetic distance among these gene families (Figure 4). Based on sequence similarity, all *CALS* genes were categorized into 3 groups (Group Ⅰ–Ⅲ). Group Ⅰ comprises nine *BoCALS* genes (*BoCALS9*, *BoCALS3*, *BoCALS1*, *BoCALS4*, *BoCALS12*, *BoCALS14*, *BoCALS7*, *BoCALS11*, *BoCALS10*) genes, nine *BrCALS* genes and six *AtCALS* genes; Group Ⅱ contains five *BoCALS* genes (*BoCALS9*, *BoCALS6*, *BoCALS2*, *BoCALS15*, *BoCALS13*), five *BrCALS* genes and five *AtCALS* genes; Group Ⅲ exclusively harbors three genes: *BoCALS8*, *BrCALS8*, and *AtCALS8*. Overall, multiple *CALS* genes from *B. oleracea* and *B. rapa* accessions, as well as *A. thaliana*, clustered on the same sub-branches of the phylogenetic tree. This indicated high homology among BoCALS, BrCALS, and AtCALS proteins. So, by inference, CALS proteins from different species occupying the same phylogenetic branch likely share similar functions [16,17]. Combined with the collinearity analysis in Section 2.3, these results collectively supported that CALS evolution is relatively conserved across these three species. The phylogenetic relationships indicated that callose synthase enzyme of different species may have evolved from a common glucan-synthase domain, and those within the same group may perform similar biological functions, but are under differential expression regulation [27].

### 2.5. Expression Profiles of BoCALS Genes in Different Tissues of B. oleracea

**Figure 5:**
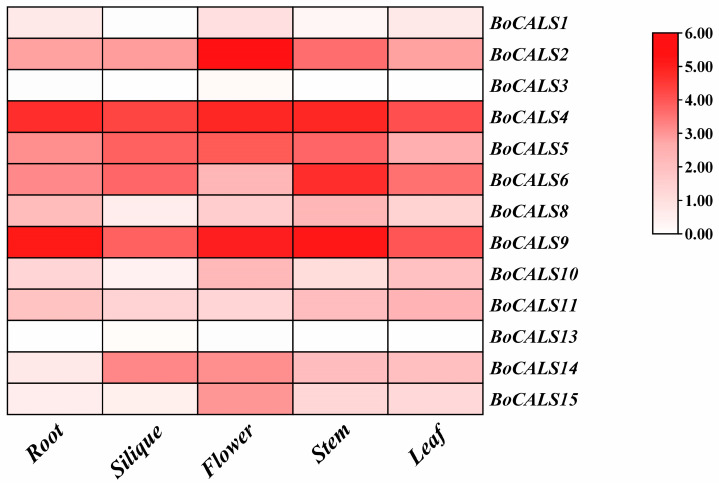
Different expression pattern of *BoCALS* genes in root, silique, flower, stem and leaf of *B. oleracea*. Based on FPKM plotting, the darker red color indicates the higher expression level of the gene.

Using the published RNA-seq dataset from the *B. oleracea* transcriptome database (GSM1052958-964), we analyzed the expression levels of 15 *BoCALS* genes across five major tissues of susceptible variety: roots, siliques, flowers, stems and leaves. A heatmap was constructed using their expression profiles to visualize the spatial expression distribution and to infer potential gene functions in different developmental context (Figure 5). The results revealed that *BoCALS3* and *BoCALS13* showed no detectable expression in any of the test tissues, implying that they may be specifically expressed at certain developmental stages or under particular environmental conditions. On the contrary, *BoCALS4* and *BoCALS9* exhibited high expression level across all tissues, indicating their possible involvement in both vegetative growth and reproductive development. Notably, the expression level of *BoCALS2* and *BoCALS15* was significantly higher in flowers than other tissues, indicating that their potential roles in floral organ development or related biological developmental processes. The elevated expression of *BoCALS14* in flowers and siliques suggested its potential association with reproductive development in *B. oleracea*, maybe through callose-mediated processes.

### 2.6. Relative Expression Analysis of BoCALS Genes Under H. parasitica Challenge

**Figure 6:**
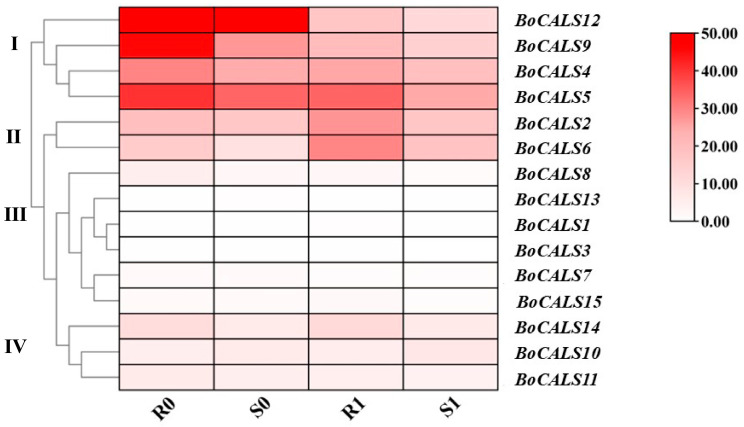
Heatmap of different expressions of the *BoCALS* genes in response to *H. parasitica* infection. R0 and R1 represent the resistant variety 20-2221 before and four days after inoculation with *H. parasitica*, respectively, and S0 and S1 represent the susceptible variety 20-2229 before and four days after inoculation with *H. parasitica*, respectively. Based on FPKM plotting, the darker red color indicates the higher expression level of the gene.

To elucidate the different expressions of the *BoCALS* genes in response to *H. parasitica* infection, we conducted inoculation experiments with the resistant material 20-2221 and the susceptible material 20-2229 of *B. oleracea.* Subsequently, we observed the phenotypes of these two plant materials at four days post inoculation (dpi), by which time mycelial colonization became clearly detectable in susceptible plants. Based on the previously released RNA-seq data (BioProject ID: PRJNA1146208) performed by our research group, we found that the expression of *BoCALS* gene was significantly different between resistant and susceptible materials before and after infection by *H. parasitica* [28]. To investigate the role of *BoCALS* genes in cabbage’s response to *H. parasitica* infection, we plotted a clustered heatmap (Figure 6) of *BoCALS* genes expression in resistant and susceptible materials before and after *H. parasitica* infection. As shown in Figure 6, 15 *BoCALS* genes were divided into four groups according to the different expressions. Genes in Cluster Ⅰ showed high expression level in *B. oleracea* leaves, and the expression level in resistant material were significantly higher than those in susceptible material. After four days of *H. parasitica* infection, the expression of all these genes decreased significantly, which suggests that the infection of *H. parasitica* suppressed their expression. Genes in Cluster Ⅱ showed elevated expression in both resistant and susceptible varieties after four days of *H. parasitica* infection, suggesting that the stress of downy mildew upregulated their expression. The above result means that these two genes may play a role in resistance to downy mildew in *B. oleracea*. No detectable expression of genes in Cluster III was observed in *B. oleracea* leaves, which further supports the reliability of the phylogenetic classification presented in the previous section. Genes in Cluster Ⅳ were expressed at a low level in the two materials, and they expressed at similar levels before and after infection in susceptible and resistant varieties. The above results demonstrating that the genes in Clusters Ⅰ and Ⅱ may be involved in the response to downy mildew infection, whereas those in Clusters Ⅲ and Ⅳ may not be involved in the regulation of resistance to downy mildew in *B. oleracea*.

**Figure 7:**
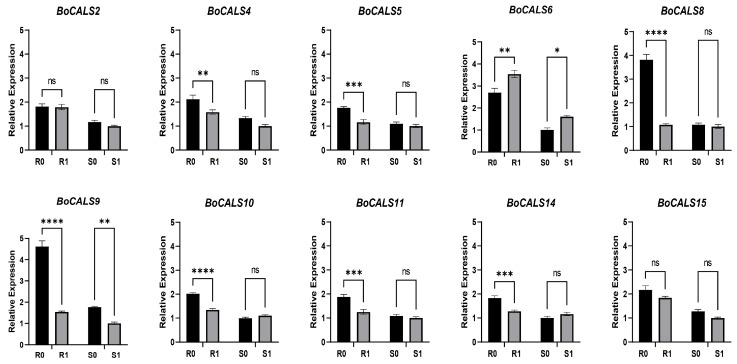
qRT-PCR analysis of the *BoCALS* genes in response to *H. parasitica* infection. QRT-PCR expression profiles of *BoCALS* genes before and four days after *H. parasitica* infection, with expression levels normalized to the *Boactin* internal reference gene. Stars above the bars indicate the significance of differences between treatments, “ns” indicates no significant difference, A single star (*) indicates significant level (*p* < 0.05), two stars (**) indicates significant level (*p* < 0.01), three stars (***) indicates highly significant level (*p* < 0.001), and four stars (****) indicates highly significant level (*p* < 0.0001).

To validate the accuracy of the RNA-seq data, we randomly selected ten *BoCALS* genes for qRT-PCR reaction. The results showed that before and after inoculation, the expression of all the genes was higher in the resistant varieties than in the susceptible varieties (Figure 7). Most of genes showed a significant decrease in expression after inoculation with *H. parasitica*. Notably, the expression of *BoCALS6* was higher in resistant varieties than in susceptible varieties and exhibited a significant up-regulation after *H. parasitica* infection, which agress with the above RNA-seq results (Figure 6).

### 2.7. Cis-Acting Element Analysis of BoCALS6

The *CALS* gene plays a critical role in plant resistance to biotic and abiotic stresses and is putatively regulated by phytohormones. Analysis of the 2000-bp upstream region of the *BoCALS6* promoter (Supplementary Table S1) identified multiple phytohormone-responsive cis-elements, including: abscisic acid responsive element (ABRE) and MeJA-responsive motifs (CGTCA-motif and TGACG-motif). Additionally, stress-associated cis-regulatory elements were detected, such as anaerobic induction essential and low-temperature responsive cis-acting elements. Photosynthesis-related elements—including light responsiveness cis-acting elements and palisade mesophyll cell differentiation elements—were also present. These cis-acting elements in the *BoCALS6* promoter suggest transcriptional regulation by diverse stress-related hormones and environmental factors, contributing to plant growth, development, and stress response.

## 3. Discussion

### 3.1. Characterization of BoCALS Family Genes in B. oleracea

As described above, a total of 15 *BoCALS* genes were identified in the *B. oleracea* genome. Domain analysis revealed that all *BoCALS* family members possess both the 1,3-beta-glucan-synthase (Glu) domain and the Fks1 domain. The Glu domain is known to catalyze callose synthesis [29], while the Fks1 domain is involved in membrane interaction via lipid binding and curvature distortion [25]. These characteristics strongly support the predicted plasma membrane localization of BoCALS proteins and strongly imply their identity as transmembrane enzymes. The universal conservation of these core domains across all *BoCALS* members provides strong evidence for their functional homogeneity within the family. Interestingly, *BoCALS* genes grouped within Cluster Ⅰ additionally encode the Vta1 domain, which has been previously linked to vesicle trafficking and regulation of callose biosynthesis [26]. Multiple conserved motifs were found in the *BoCALS* gene family, indicated high similarity in their protein sequences. Substantial variation in intron number was observed among *BoCALS* genes. For instance, *BoCALS12* and *BoCALS14* possess only one to two introns, contrasting sharply with *BoCALS10*, which harbors as many as 52 introns. Introns are ancient and ubiquitous components of eukaryotic genomes [30]. Genes with high intron density may have retained ancestral multifunctional elements [31], whereas intron-poor genes may have undergone evolutionary intron loss [32]. Interestingly, although *BoCALS12* and *BoCALS14* share similar intron numbers, *BoCALS12* exhibited significantly higher basal expression than *BoCALS14* in uninfected cabbage plants. This differential expression may be attributed to either: the presence of additional cis-regulatory elements in *BoCALS12*, such as intronic or promoter-proximal enhancers [33], or more efficient transcriptional elongation due to sequence specific features like Zinc Finger Homeodomain (*ZF-HD*) [34].

### 3.2. Multiple Functional Roles of the CALS Gene Family in Plant Development

Given the close phylogenetic relationship among *B. rapa*, *B. oleracea* and *A. thaliana*, we constructed a phylogenetic tree to analyze the genetic distance of *CALS* genes across these three species. Interestingly, when we constructed a phylogenetic tree using the CALS genes from *B. oleracea*, *B. rapa*, and *A. thaliana*, all *CALS* genes were grouped into three distinct clades. Specifically: Clade I correspond to Clade II in Figure 1; Clades II align with Clade I in Figure 1; Clade III contains three divergent orthologs: *BoCALS8* (*B. oleracea*), along with one orthologous gene each from *B. rapa* (*BrCALS8*) and *A. thaliana* (*AtCALS8*).

Callose is ubiquitously distributed across the tissues of higher plants [9]. Previous studies have demonstrated that the *CALS* genes are involved not only in responses to biotic and abiotic stresses but also in regulating microspore and pollen development [2]. Members of the *CALS* gene family exhibit distinct expression patterns across plant tissues and developmental stages. For instance, in *Arabidopsis thaliana*, *AtCALS1*, *AtCALS2*, *AtCALS3*, *AtCALS7*, *AtCALS8* and *AtCALS10* participate in callose deposition on the cell plate during cytokinesis [19,35,36,37,38,39,40], as their orthologous counterparts, *BoCALS2*, *BoCALS4*, *BoCALS5*, *BoCALS6*, *BoCALS7* and *BoCALS8* may play the same role in *B. oleracea*. In contrast, *AtCALS5*, *AtCALS9*, *AtCALS10*, *AtCALS11*, and *AtCALS12* function in sporogenesis and gametogenesis, particularly by encoding isoforms essential for pollen development [40,41,42,43,44,45,46,47], the function of their putatively orthologous genes, *BoCALS1*, *BoCALS4*, *BoCALS9*, *BoCALS12*, *BoCALS13* and *BoCALS14* for pollen development are worthy of further exploring. Among these, *AtCALS9*, *AtCALS10*, and *AtCALS12* are implicated in both sporophytic and gametophytic processes, while *AtCALS5*, *AtCALS11*, and *AtCALS12* are responsible for synthesizing the interstitial callose wall between tetraspores during pollen maturation. Additionally, *AtCALS5* contributes to the formation of the outer wall of pollen grains and the structural integrity of pollen tubes. However, functional redundancy may exist among certain members with overlapping roles [47]. During the meiosis and tetrad stages of Chinese cabbage development, the high expression of *CALS* gene *BraA01g041620* leads to excessive callose accumulation, resulting in microspore abortion in Chinese cabbage with nuclear sterility near-isogenic lines [14]. Identifying the orthologous gene of this gene in cabbage will help to understand the potential role of the *BoCALS* gene in pollen development in *B. oleracea*.

Given the critical involvement of callose synthase in microspore development, their impact on plant fertility has been extensively studied. For example, knockdown of the *AtCALS9* gene in *A. thaliana* resulted in defective male gametophytes, irregular callose deposition in bicellular pollen, premature germination within anthers, and ultimately the failure to generate homozygous progeny [43]. Similarly, disruption of *GSL5* gene in rice abolished callose deposition in meiotic cell walls and tetrameric cell plates, leading to aberrant pollen development and significantly reduced fertility [48]. In maize, mutation of the *ZmCals12* gene via CRISPR/Cas9-induced genome editing caused male sterility, as evidenced by pollen abortion and a reduction in plant height. These phenotypic outcomes suggest its utility as a promising genetic resource for hybrid seed production in maize breeding programs [49].

### 3.3. The Multiple Roles and Mechanisms of the CALS Gene Family in Stress Responses in Higher Plants

Callose is typically deposited within the cell walls of higher plants, where it serves as a physical barrier to protect plants against adverse environmental stress. Under adverse conditions, the expression of *CALS* genes is modulated, resulting in the localized deposition of callose [50]. This process contributes to the formation of protruding cell wall structures that impede the penetration of the cell wall-degrading enzymes secreted by pathogens and parasitic microorganisms, as well as the diffusion of other virulence factors, thereby enhancing plant stress resistance [51]. Upon the alleviation of stress, callose is subsequently degraded to restore normal physiological functions [5].

We propose that *CALS* gene family members serve diverse functions in plant stress responses. For example, in a germplasm screening for resistance to the green peach aphid (GPA, *Myzus persicae*), resistant pepper lines exhibited upregulated *CALS* gene expression and induced callose deposition following aphid feeding, whereas susceptible lines showed no such response [52]. In cotton (*Gossypium hirsutum*), overexpression of *GhCALS5* promoted callose accumulation in leaves, thereby enhancing resistance to aphid infection [53]. In kidney beans (*Phaseolus vulgaris*), elevated atmospheric CO_2_ levels induced the expression of *CALS3* and *CALS10*, resulting in increased callose deposition and enhanced resistance to thrips (*Frankliniella occidentalis*) [54]. *A. thaliana* plasmodesmata-located protein 5 (*PDLP5*, also known as HOPW1-1-INDUCED GENE1) stimulates callose deposition to reduce PD permeability by mediating crosstalk between PD regulation and salicylic acid signaling pathway-dependent defense responses [55]. In CLas-infected citrus, *CsCalS11* expression exhibited a significant positive correlation (*p* < 0.01) with callose deposition and foliar starch content, demonstrating that the CsABI5-CsCalS11 regulatory complex orchestrates callose biosynthesis via ABA-mediated signal transduction pathways [56].

Downy mildew, caused by obligate oomycete pathogen, represents a major threat to horticultural crop production by significantly reducing yield and quality [57]. However, only a limited number of genes associated with downy mildew resistance have been identified in *B. oleracea*, including *BoDMR2* and *BoCML46-2* [24,58]. Notably, our results showed that *BoCALS6* was significantly up regulated in both resistant and susceptible accessions following *H. parasitica* infection, which may indicate a potentially critical role in host defense response. Phylogenetic analysis demonstrated that *BoCALS6* is orthologous to *AtCALS1*, which has been identified as a target of the Plasmodiophora brassicae effector RxLR3 [59]. Furthermore, previous studies have shown that in *Arabidopsis thaliana*, *H. parasitica* pathogen-induced transcriptional upregulation of *CALS1* and *CALS12* requires functional salicylic acid (SA) biosynthesis via ICS1 (*SID2*), coupled with NPR1-dependent signaling to potentiate immune-responsive callose synthase activation [60]. Analysis of cis-acting elements in the *BoCALS6* promoter region revealed the presence of multiple hormone-responsive elements, including ABA-responsive element (ABRE), MeJA-responsive motifs (CGTCA-motif and TGACG-motifs), WRKY transcription factor-binding element (W-box), as well as numerous MYB transcription factor response elements (Supplementary Table S1). Previous studies indicated that *CALS* genes may mediate plant disease resistance and stress tolerance through hormone signaling pathways, while WRKY transcription factors are key regulators of abiotic stress responses [55,56,60,61]. These findings may suggest the regulatory mechanism governing *BoCALS6* expression during downy mildew infection in *B. oleracea*, potentially involving hormone-mediated defense signaling and transcription factor networks. In conclusion, these results extend our understanding of the role of *BoCALS* genes in plant stress responses and highlight the functional importance of *BoCALS6* in the defense against downy mildew in *B. oleracea*. These insights contribute to the broader characterization of the *CALS* gene family and lay the groundwork for future studies on the molecular mechanisms underlying plant-pathogen interactions.

However, members of gene families often share conserved protein domains enabling participation in similar biochemical processes [47,62]. Notably, within the *BoCALS* gene family, *BoCALS6* exhibits the closest sequence similarity to *BoCALS5*. Furthermore, RNA-seq analysis (Figure 6) demonstrated that *BoCALS5* expression levels were higher in both susceptible and resistant lines after infection with downy mildew, particularly in susceptible lines. While *BoCALS6* expression was specifically induced, *BoCALS5* maintained constitutively high expression levels. This observation suggests *BoCALS5* may functionally compensate for *BoCALS6* in susceptible genotypes.

These interpretations, based on current literature and experimental data, present a testable hypothesis. Future work will employ targeted approaches, such as knockout or knockdown of key genes (e.g., *BoCALS5*, *BoCALS6*), to definitively elucidate the function and mechanism of *BoCALS* genes in cabbage downy mildew resistance. At the same time, investigations will characterize the tissue-specific expression patterns of *BoCALS6* during *H. parasitica* infection. This spatio-temporal expression analysis will further elucidate the regulatory mechanisms involved in the resistance to downy mildew of *B. oleracea*.

## 4. Materials and Methods

### 4.1. Genome-Wide Identification of the BoCALS Genes in B. oleracea

The protein sequences of the 12 *AtCALS* genes (Supplementary File S1) of *A. thaliana* were retrieved from The Arabidopsis Information Resource (TAIR; https://www.arabidopsis.org/ (accessed on 20 October 2024)) database [63] and used as queries to identify *CALS* homologs in the *B. oleracea* genome through the Brassica Database (BRAD; http://www.brassicadb.cn/ (accessed on 21 October 2024)) [64]. Sequence similarity searches were conducted using the BLASTP (version 2.16.0) program [65]. Candidate genes were subsequently validated based on the presence of conserved domains using multiple protein domain databases, including Pfam [66], InterPro [67], the NCBI Conserved Domain Database (CDD) [68,69] and SMART (version 9) [70,71].

### 4.2. Gene Structure, Physicochemical Characterization, and Subcellular Localization Prediction and Cis-Acting Element Analysis of the BoCALS Proteins

The physicochemical properties of BoCALS proteins, including amino acid length, relative molecular weight (MW), theoretical isoelectric point (pl), and the Grand Average of Hydropathicity (GRAVY), were predictively analyzed using the ProtParam tool (https://web.expasy.org/protparam/ (accessed on 22 October 2024)) [72]. Subsequently, the subcellular localization of each BoCALS protein was predicted using the BUSCA web server (http://busca.biocomp.unibo.it/ (accessed on 22 October 2024)) [73]. Conserved domains were predicted using the MEME Suite (https://web.mit.edu/meme/current/share/doc/meme.html (accessed on 23 October 2024)) [74], with the maximum number of domains set to ten under default parameters. Exon-intron structures were analyzed by Gene Structure Display Server (GSDS 2.0; https://gsds.gao-lab.org/ (accessed on 23 October 2024)). Finally, the above information was visualized using TBtools (version 2.145) [75]. Additionally, a 2000-bp nucleotide sequence upstream of the *BoCALS6* transcription start site (TSS) was retrieved from the Brassica Database (BRAD; http://www.brassicadb.cn/ (accessed on 25 October 2024)) [64]. This sequence was analyzed utilizing the PlantCARE online tool (https://bioinformatics.psb.ugent.be/webtools/plantcare/html/ (accessed on 27 October 2024)) [76] to predict putative cis-acting elements in *BoCALS6* promoter region.

### 4.3. Chromosomal Localization and Collinearity Analysis

The GFF3 file, genome FASTA file, and *BoCALS* gene ID file were downloaded from the *Brassica oleracea* Genome JZS 2.0 database. Chromosome localization of *BoCALS* genes was conducted using the “Gene Location Visualize from GTF/GFF” module in TBtools, by inputting the GFF3 file and the *BoCALS* gene ID list. The *BoCALS* genes were designated as *BoCALS1*-*BoCALS15* according to their physical position on the chromosomes. To perform collinearity analysis, the genomic FASTA files of *B. oleracea* and *A. thaliana* were first submitted to the “Fasta Stats” module to generate the corresponding annotation files. These annotation files, together with the genomic FASTA files, were then used as input in the “One Step MCScanX-Super Fast” module in TBtools for collinearity analysis [75]. Finally, chromosome localization and syntenic relationships were visualized using the “Advanced Circos” module.

### 4.4. Protein Sequence Alignment and Phylogenetic Analysis

Twelve AtCALS protein sequences from the TAIR database were used as queries to identify homologous genes in *B. oleracea* via BLASTP search, which used default parameters with E-value threshold of less than 1 × 10^−20^ and a minimum score of 1000 [65]. The BrCALS protein sequences were obtained from previously published research [14]. Multiple sequence alignment of BoCALS, AtCALS and BrCALS protein (Supplementary File S1) was performed using ClustalW (version 2.1) software with default parameters [77]. Genetic distances among species were calculated using MEGA 12 software (https://megasoftware.net/ (accessed on 1 November 2024)) [78], and the phylogenetic tree was constructed based on the neighbor-joining (NJ) method.

### 4.5. Expression Analysis of BoCALS Genes Using Published RNA-Seq Data

To investigate tissue-specific expression profiles of *BoCALS* genes, publicly available RNA-Seq datasets (GSM1052958-964) were downloaded from the NCBI database. These data comprise transcriptomes from multiple *B. oleracea* tissues, including roots, siliques, flowers, stems and leaves—of susceptible cabbage material maintained without *H. parasitica* inoculation. The expression levels of the genes were quantified using the fragments per kilobase of transcript per million mapped reads (FPKM) method, and the resulting data were normalized using the FPKM algorithm. A heatmap of tissue-specific expression was constructed using the heatmap plug-in in TBtools software [75].

To elucidate differential expression patterns of *BoCALS* genes during *H. parasitica* infection, RNA-seq data (BioProject ID: PRJNA1146208) previously released by our research group were utilized. These data comprise transcriptomes from leaves of resistant material (20-2221) and susceptible material (20-2229) cabbage lines sampled before inoculation with *H. parasitica* and four days post inoculation (4 dpi).

### 4.6. Plant Materials and Treatment

Seeds of downy mildew resistant variety 20-2221 and susceptible variety 20-2229 were sown in 10 cm × 10 cm seedling trays containing sterilized germination substrate. Inoculation with *H. parasitica* was performed out when seedlings developed two true leaves. The strain of *H. parasitica* we used in this study was propagated and preserved in our laboratory. Prior to inoculation, spores were harvested from infected plants of the susceptible variety 20-2229 and prepared as a suspension at a concentration of 1 × 10^5^ spores/mL. The inoculum was uniformly sprayed onto the abaxial surface of the true leaves using a handheld sprayer. Each treatment group consisted of 10 seedlings, and three independent biological replications were conducted. The inoculated plants were maintained at 23–25 °C and 95% relative humidity. They were first kept in complete darkness for 24 h, followed by incubation in a greenhouse under a 16 h light/8 h dark photoperiod. Phenotypes of resistant and susceptible plants were monitored daily throughout this period. At four days post-inoculation (dpi), visible mycelial colonization was evident on susceptible plants. Consequently, leaves were harvested from both control and inoculated plants. Harvested leaves were immediately flash-frozen in liquid nitrogen and stored in an ultra-low temperature refrigerator at −80 °C for subsequent RNA-seq and qRT-PCR analysis.

### 4.7. RNA Extraction, cDNA Synthesis and Quantitative Real-Time PCR Analysis

Total RNA was extracted from cabbage leaves that were collected and stored at −80 °C using the TIANGEN RNAprep Pure Plant Kit according to the instructions (TIANGEN, Beijing, China). Subsequently, the purity and quality of the extracted RNA were assessed using a spectrophotometer (BioDrop, Cambridge, UK) and 1% agarose gel electrophoresis. First-strand cDNA was synthesized using HiScript^®^ III RT SuperMix for qPCR (+gDNA wiper) Kit (Vazyme, Nanjing, China), following the manufacturer’s instructions. Specific primers for *BoCALS* genes were designed using the PrimerQuest^™^ Tool from the Integrated DNA Technologies (IDT, https://www.idtdna.com/ (accessed on 24 December 2024)) (Supplementary Table S2). Quantitative real-time PCR (qRT-PCR) was carried out using the SupRealQ Purple Universal SYBR qPCR Master Mix (U+) enzyme (Vazyme, China) and using the Applied Biosystems QuantStudio^™^ 6 Flex System (Thermo Fisher Scientific, Waltham, MA, USA). The house-keeping gene *BoActin* was selected as the internal control, and each reaction was performed in three technical replicates. The PCR program was performed as follows: initial denaturation at 95 °C for 30 s, followed by 40 cycles of 95 °C for 10 s and 60 °C for 20 s, with a final melt curve stage of 95 °C for 15 s and 60 °C for 60 s. The relative expression levels of the target genes were calculated using the 2^−∆∆Ct^ method [79].

## 5. Conclusions

In this study, 15 *BoCALS* genes were identified in the *B. oleracea* genome based on homology with *Arabidopsis*, and these genes were found to be unevenly distributed across eight chromosomes. Bioinformatic analysis revealed that the BoCALS proteins vary in their amino acid length, relative molecular weight (MW), theoretical isoelectric point (PI), the Grand Average of Hydropathicity (GRAVY) and other characteristics. Subcellular localization predictions indicated that all BoCALS proteins are targeted to the plasma membrane. Gene structure phylogenetic analysis demonstrated that *BoCALS* genes share high sequence similarity and possess conserved domains. Expression profiling based on transcriptome data and qRT-PCR analysis revealed distinct expression patterns between susceptible and resistant varieties of *B. oleracea* before and after *H. parasitica* inoculation. Notably, *BoCALS6* had a higher expression level in resistant materials than in susceptible materials and showed significant up-regulation in response to pathogen stress, suggesting that it may play a key role in downy mildew resistance. Collectively, this study provides a comprehensive characterization of *BoCALS* gene family and offers a solid foundation for further functional studies. These findings also contribute valuable insights into the molecular mechanisms of downy mildew resistance in *B. oleracea*, thereby supporting the development of disease-resistant cultivars through molecular breeding approaches.

## Figures and Tables

**Table 1:** Summary information and Characterization of BoCALS proteins in *B. oleracea*.

Protein ID	Simplified ID	Peptide Length (Amino Acid Residues)	MW (kDa)	Isoelectric Points (pl)	Subcellular Localization (BUSCA)	Grand Average of Hydropathicity (GRAVY)
BolC01g053890.2J	BoCALS1	1872	214.95	8.45	plasma membrane	−0.046
BolC02g005380.2J	BoCALS2	1954	225.84	9.09	plasma membrane	−0.122
BolC03g038840.2J	BoCALS3	1951	223.85	8.78	plasma membrane	0.019
BolC04g011790.2J	BoCALS4	1908	218.90	8.73	plasma membrane	0.021
BolC04g017120.2J	BoCALS5	2043	236.67	9.20	plasma membrane	−0.046
BolC05g003830.2J	BoCALS6	1960	226.98	9.16	plasma membrane	−0.069
BolC05g004600.2J	BoCALS7	1947	226.58	8.85	plasma membrane	−0.169
BolC05g051770.2J	BoCALS8	1960	227.86	8.59	plasma membrane	−0.124
BolC05g058550.2J	BoCALS9	1911	219.20	8.41	plasma membrane	−0.052
BolC06g024740.2J	BoCALS10	2312	267.09	8.85	plasma membrane	−0.076
BolC08g058150.2J	BoCALS11	2137	247.27	8.98	plasma membrane	−0.219
BolC09g002840.2J	BoCALS12	1783	207.22	9.21	plasma membrane	−0.032
BolC09g011540.2J	BoCALS13	1920	220.22	9.05	plasma membrane	−0.072
BolC09g029770.2J	BoCALS14	1768	204.90	9.15	plasma membrane	0.069
BolC09g060050.2J	BoCALS15	2010	232.54	9.13	plasma membrane	−0.078

## Data Availability

All data generated or analyzed during this study are included in this published article and its Supplementary Information Files. The raw sequencing data used during this study are available in the NCBI SRA database (BioProject number: PRJNA1146208). The *B. oleracea* reference genome ‘Braol JZS V2.0’ used in this study can be found at the link http://brassicadb.cn/#/ (accessed on 21 October 2024). The A. thaliana genome can be found at the link https://www.arabidopsis.org/index.jsp (accessed on 20 October 2024). The protein database of National Center for Biotechnology Information (NCBI) can be found at the link https://www.ncbi.nlm.nih.gov (accessed on 21 October 2024). All these databases are open to public access.

## Supplementary Materials

The following supporting information can be downloaded at: https://www.mdpi.com/article/10.3390/ijms262110304/s1.
