# Supplementary material for: Genome-Wide Analysis of Callose Synthase (CALS) Genes in Cabbage (Brassica oleracea var. capitata L.): Identification and Expression Profiling During Hyaloperonospora parasitica Infection
Source: Int J Mol Sci. 2025 Oct 23;26(21):10304. doi: 10.3390/ijms262110304 (PMC12610245; doi:10.3390/ijms262110304)
Supplement: Supplementary file 1 [file ijms-26-10304-s001.zip › Supplementary File S1. Protein sequence of AtCALS, BoCALS and BrCALS.pdf]

Supplementary File S1: Protein sequence of AtCALS, BoCALS and BrCALS

1. AtCALS

>AT1G06490/AtCALS7
MASTSSGGRGEDGRPPQMQPVRSMSRKMTRAGTMMIEHPNEDERPIDSELVPSSLASIAPILRVANDIDQDN
ARVAYLCRFHAFEKAHRMDPTSSGRGVRQFKTYLLHKLEEEEEITEHMLAKSDPREIQLYYQTFYENNIQDGEGK
KTPEEMAKLYQIATVLYDVLKTVVPQARIDDKTLRYAKEVERKKEQYEHYNILPLYALGAKTAVMELPEIKAAILAV
CNVDNLPRPRFHSASANLDEVDRERGRSFNDILEWLALVFGFQRGNVANQREHLILLLANIDVRKRDLENYVEI
KPSTVRKLMEKYFKNYNSWCKYLRCDSYLRFPAGCDKQQLSLLYIGLYLLIWGEASNVRFMPECLCYIFHNMAN
EVHGILFGNVYPVTGDTYEAGAPDEEAFLRNVITPIYQVLRKEVRRNKNGKASHSKWRNYDDLNEYFWDKRCF
RLKWPMNFKADFFIHTDEISQVPNQRHDQVSHGKRKPKTNFVEARTFWNLYRSFDRMWMFLVLSLQTMIIVA
WHPSGSILAIFTEDVFRNVLTIFITSAFLNLLQATLDLVLSFGAWKSLKFSQIMRYITKFLMAAMWAIMLPITYSKS
VQNPTGLIKFFSSWVGSWLHRSLYDYAIALYVLPNILAAVFFLLPPLRRIMERSNMRIVTLIMWWAQPKLYIGRG
MHEEMFALFKYTFFWVMLLLSKLAFSYYVEILPLVNPTKLIWDMHVVNYEWHEFFPNATHNIGVIIAIWGPIVL
VYFMDTQIWYAIFSTLFGGIYGAFSHLGEIRTLGMLRSRFKVVPSAFCSKLTPLPLGHAKRKHLDETVDEKDIARFS
QMWNKFIHTMRDEDLISDRERDLLLVPSSSGDVTVVQWPPFLLASKIPIALDMAKDFKGKEDVDLFKKIKSEYY
MHYAVVEAYETVRDIIYGLLQDESDKRIVREICYEVDISIQQHRFLSEFRMTGMPLLSDKLEKFLKILLSDYEEDDYK
SQIINVLQDIIEIITQDVMVNGHEILERAHLQSGDIESDKKEQRFEKIDLSLTQNISWREKVVRLLLLLTVKESAINIP
QSLEARRRMTFFANSLFMNMPDAPRVRDMLSFSVLTPYYKEDVLYSEEELNKENEDGITILFYLQRIYPEEWSNY
CERVNDLKRNLSEKDKAEQLRQWVSYRGQTLSRTVRGMMYYRVALELQCFQEYTEENATNGGYLPSESNEDD
RKAFSDRARALADLKFTYVVSCQVYGNQKKSSESRDRSCYNNILQLMLKYPSLRVAYIDEREETVNGKSQKVFYS
VLLKGCDKLDEEIYRIKLPGPPTEIGEGKPENQNHAIIFTRGEALQTIDMNQDNYFEECFKMRNVLQEFDEGRRG
KRNPTILGLREHIFTGSVSSLAWFMSNQETSFVTIGQRVLANPLRVRFHYGHPDIFDRIFHITRGGISKASKIINLSE
DIFAGYNSTLRGGYVTHHEYIQAGKGRDVGMNQISFFEAKVANGNGEQTLSRDVYRLGRRFDFYRMLSFYFTT
VGFYFSSMITVLTVYVFLYGRLYLVLSGLEKNILQSASVHESNALEQALAAQSVFQLGFLMVLPMVMEIGLEKGFR
TALGDFIIMQLQLASVFFTFQLGTKAHYFGRTILHGGSKYRATGRGFVVFHAKFAENYRLYSRSHFVKGLELVILLV
VYQVYGTSYRSSSTYMYITFSMWFLVTSWLFAPFIFNPSGFEWQKTVDDWTDWKRWMGNRGGIGIVLDKSW
ESWWDIEQEHLKHTNLRGRVLEILLALRFLLYQYGIVYHLNIARRHTTFLVYGLSWAILLSVLLVLKMVSMGRRKF
GTDFQVMFRILKALLFLGFLSVMTVLFVVCGLTISDLFASILAFLPTGWAILLIGQALRSVFKGLGFWDSVKELGRA
YEYIMGLVIFTPIAVLSWFPFVSEFQTRLLFNQAFSRGLQISMILAGKKDKETPSTKYLGHTEESFGLEHDTNTFNH
YYLWT

>AT4G04970/AtCALS11
MRRQRPSVATARDAPSLEVYNIIPIHDFLTEHPSLRYPEVRAAAAALRIVGDLPKPPFADFTPRMDLMDWLGLLF
GFQIDNVRNQRENLVLHLANSQMRLQPPPRHPDGLDPTVLRRFRKKLLRNYTNWCSFLGVRCHVTSPIQSRH
QTNAVLNLRRELLYVALYLLIWGESANLRFMPECLCYIFHHMAMELNKVLAGEFDDMTGMPYWPSFSGDCAF
LKSVVMPIYKTVKTEVESSNNGTKPHSAWRNYDDINEYFWSKRALKSLKWPLDYTSNFFDTTPKSSRVGKTGFV
EQRSFWNVYRSFDRLWILLLLYLQAAIIVATSDVKFPWQDRDVEVALLTVFISWAGLRLLQSVLDASTQYSLVSRE
TYWLFIRLTLKFVVAVAWTVLFSVFYARIWSQKNKDGVWSRAANERVVTFLKVVFVYVIPELLALVLFIVPCIRN
WVEELNLGVVYFLTWWFYSKTFVGRGMREGLVDNVKYTLFWIIVLATKFIFSYFLQIRPLIAPTRALLNLKDATYN
WHEFFGSTHRIAVGMLWLPVILVYLMDLQIWYSIYSSLVGATIGLFSHLGEIRNIDQLRLRFQFFSSAMQFNLKPE
EHLLSPKATMLKKARDAIHRLKLRYGIGQPFNKIESSQVEATWFALIWNEIILTFREEDLISDREVELLELPPNCWN
IRVIRWPCFLLCNELLLALSQANELCDAPDHWLWSKICSSEYRRCAVMEAFDSIKFVILKIVKNGTEEESILNRLF

MEIDENVENEKITEVYKLTVLLRIHEKLISLLERLMDPEKKVFRIVNILQALYELCAWEFPKTRRSTPQLRQLGLAPI
SLEADTELLFVNAINLPPLDDVVFYRQIRRVHTILTSRDPMHNVPKNIEARERLAFFSNSLFMTMPQAPSVEKM
MAFSVLTPYYDEEVMYRQEMLRAENEDGISTLFYLQRIYEDEWVNFLERMRREGAENENDIWSKKVRDLRLW
ASYRGQTLSRTVRGMMYYYSALKKLAFLDSASEMDIRMGTQIAPEARRSYYTNDGGDNTLQPTPSQEISRMAS
GITHLLKGSEYGSAMMKFTYVVACQVYGQHKARGDHRAEEILFLMKNHDALRIAYVDEVDLGRGEVEYYSVLV
KFDQQLQREVEIYRIRLPGPLKLGEGKPENQNHALIFTRGDAIQTIDMNQDNHFEEALKMRNLLESFKTYYGIRK
PTILGVREKVFTGSVSSLAWFMSAQETSFVTLGQRVLANPLKVRMHYGHPDVFDRFWFVPRGGISKASRVINIS
EDIFAGFNCTLRGGNVTHHEYIQVGKGRDVGLNQISMFEAKVASGNGEQALSRDVYRLGHRLDFFRMLSFFYT
TVGYYFNTMLIVFTVYAFLWGRLYLALSGVEKIAKDRSSSNEALGAILNQQFIIQLGLFTALPMILENSLERGFLPAV
WDFITMQLQLASFFYTFSMGTRTHYFGRTILHGGAKYRATGRGFVVEHKKFAENYRLYARTHFIKAIELAIILLVYA
AYSPLAKSSFVYILMTISSWFLITSWIISPFLFNPSGFDWLKTVNDFDDFIAWLWSRGGLFTKADQSWFTWWNE
EQEHLKTTGVWGKLLEIILDLRFFFFQYSIVYHLRIAENRTSIGVYLISWGCIIGIVAIYITTIYAQKRYSVKEHIKYRFI
QFLVILLTVLVVVMMLQFTKLTVVDLLISLLAFVPTGWGLISIAQVLKPFLLSTVVWDTVISVARFYDLFFGLIVMA
PVALLSWLPGFQNMQTRILFNEAFSRGLQISIILAGKKST

>AT5G13000/AtCALS3
MSATRGGPDQGPSQPQQRRIIRTQTAGNLGESFDSEVVPSSLVEIAPILRVANEVESSNPRVAYLCRFYAFEKAH
RLDPTSSGRGVRQFKTALLQRLEREHDPTLMGRVKKSDAREMQSFYQHYYKKYIQALHNAADKADRAQLTKAY
QTANVLFEVLKAVNLTQSIEVDREILEAQDKVAEKTQLYVPYNILPLDPDSANQAIMRYPEIQAAVLALRNTRGLP
WPEGHKKKKDEDMLDWLQEMFGFQKDNVANQREHLILLLANVHIRQFPKPDQQPKLDDQALTEVMKKLFK
NYKKWCKYLGRKSSLWLPTIQQEMQQRKLLYMALYLLIWGEAANLRFMPECLCYIYHHMAFELYGMLAGNVS
PMTGENVKPAYGGEEDAFLRKVVTPIYEVIQMEAQRSKKGKSKHSQWRNYDDLNEYFWSVDCFRLGWPMRA
DADFFCLPVAVPNTEKDGDNSKPIVARDRWVGKVNFVEIRSFWHVFRSFDRMWSFYILCLQAMIIMAWDGG
QPSSVFGADVFKKVLSVFITAAIMKLGQAVLDVILNFKAHQSMTLHVKLRYILKVFSAAAWVIILPVTYAYSWKDP
PAFARTIKSWFGSAMHSPSLFIIAVVSYLSPNMLAGVMFLFPLLRRFLERSNYRIVMLMMWWSQPRLYVGRG
MHESAFSLFKYTMFWVLLIATKLAFSYYIEIRPLVAPTQAIMKARVTNFQWHEFFPRAKNNIGVVIALWAPIILVY
FMDSQIWYAIFSTLFGGIYGAFRRLGEIRTLGMLRSRFESLPGAFNDRLIPDGKNQQKKKGIRATLSHNFTEDKVP
VNKEKEAARFAQLWNTIISSFREEDLISDREMDLLLVPYWADRDLDLIQWPPFLLASKIPIALDMAKDSNGKDRE
LKKRIESDTYMKCAVRECYASFKNIIKFVVQGNREKEVIEIIFAEVDKHIDTGDLIQEYKMSALPSLYDHFVKLIKYLL
DNKEEDRDHVVILFQDMLEVVTRDIMMEDYNISSLVDSSHGGTWHGGMIPLEQQYQLFASSGAIRFPIEPVTE
AWKEKIKRIYLLLTTKESAMDVPSNLEARRRISFFSNSLFMDMPMAPKVRNMLSFSVLTPYYTEEVLFSLRDLETP
NEDGVSILFYLQKIFPDEWNNFLERVKCLSEEELKESDELEEELRLWASYRGQTLTRTVRGMMYYRKALELQAFL
DMAMHEDLMEGYKAVELNSENNSRGERSLWAQCQAVADMKFTYVVSCQQYGIHKRSGDPRAQDILRLMTR
YPSLRVAYIDEVEEPVKDKSKKGNQKVYYSVLVKVPKSTDHSTLAQNLDQVIYRIRLPGPAILGEGKPENQNHAIIF
SRGEGLQTIDMNQDNYMEEALKMRNLLQEFLTKHDGVRHPSILGLREHIFTGSVSSLAWFMSNQETSFVTIGQ
RLLANPLRVRFHYGHPDVFDRLFHLTRGGVSKASKVINLSEDIFAGFNSTLREGNVTHHEYIQVGKGRDVGLNQI
SMFEAKIANGNGEQTLSRDIYRLGHRFDFFRMMSCYFTTVGFYFSTLITVLTVYIFLYGRLYLVLSGLEQGLSTQKG
IRDNTPLQIALASQSFVQIGFLMALPMLMEIGLERGFRTALSEFVLMQLQLAPVFFTFSLGTKTHYYGRTLLHGG
AKYRSTGRGFVVFHAKFADNYRLYSRSHFVKGLEMMLLLVVYQIFGSAYRGVLAYLLITISMWFMVGTWLFAPF
LFNPSGFEWQKIVDDWTDWNKWINNIGGIGVPAEKSWESWWEEEQEHLRYSGKRGIVVEILLALRFFIYQYGL
VYHLTITEKTKNFLVYGVSWLVIFLILFVMKTVSVGRRRFSASFQLMFRLIKGLIFMTFIAIIVILITLAHMTIQDIIVCI
LAFMPTGWGMLLIAQACKPVVHRAGFWGSVRTLARGYEIVMGLLLFTPVAFLAWFPFVSEFQTRMLFNQAFS
RGLQISRILGGHRKDRSSRNKE

>AT4G03550/AtCALS12
MSLRHRTVPPQTGRPLAAEAVGIEEEPYNIIPVNNLLADHPSLRFPEVRAAAAALKTVGDLRRPPYVQWRSHYD
LLDWLALFFGFQKDNVRNQREHMVLHLANAQMRLSPPPDNIDSLDSAVVRRFRRKLLANYSSWCSYLGKKSN
IWISDRNPDSRRELLYVGLYLLIWGEAANLRFMPECICYIFHNMASELNKILEDCLDENTGQPYLPSLSGENAFLT
GVVKPIYDTIQAEIDESKNGTVAHCKWRNYDDINEYFWTDRCFSKLKWPLDLGSNFFKSRGKSVGKTGFVERRT
FFYLYRSFDRLWVMLALFLQAAIIVAWEEKPDTSSVTRQLWNALKARDVQVRLLTVFLTWSGMRLLQAVLDAAS
QYPLVSRETKRHFFRMLMKVIAAAVWIVAFTVLYTNIWKQKRQDRQWSNAATTKIYQFLYAVGAFLVPEILALAL
FIIPWMRNFLEETNWKIFFALTWWFQGKSFVGRGLREGLVDNIKYSTFWIFVLATKFTFSYFLQVKPMIKPSKLL
WNLKDVDYEWHQFYGDSNRFSVALLWLPVVLIYLMDIQIWYAIYSSIVGAVVGLFDHLGEIRDMGQLRLRFQF
FASAIQFNLMPEEQLLNARGFGNKFKDGIHRLKLRYGFGRPFKKLESNQVEANKFALIWNEIILAFREEDIVSDRE
VELLELPKNSWDVTVIRWPCFLLCNELLLALSQARELIDAPDKWLWHKICKNEYRRCAVVEAYDSIKHLLLSIIKV
DTEEHSIITVFFQIINQSIQSEQFTKTFRVDLLPKIYETLQKLVGLVNDEETDSGRVVNVLQSLYEIATRQFFIEKKTT
EQLSNEGLTPRDPASKLLFQNAIRLPDASNEDFYRQVRRLHTILTSRDSMHSVPVNLEARRRIAFFSNSLFMNMP
HAPQVEKMMAFSVLTPYYSEEVVYSKEQLRNETEDGISTLYYLQTIYADEWKNFKERMHREGIKTDSELWTTKL
RDLRLWASYRGQTLARTVRGMMYYYRALKMLAFLDSASEMDIREGAQELGSVRNLQGELGGQSDGFVSEND
RSSLSRASSSVSTLYKGHEYGTALMKFTYVVACQIYGSQKAKKEPQAEEILYLMKQNEALRIAYVDEVPAGRGETD
YYSVLVKYDHQLEKEVEIFRVKLPGPVKLGEGKPENQNHAMIFTRGDAVQTIDMNQDSYFEEALKMRNLLQEY
NHYHGIRKPTILGVREHIFTGSVSSLAWFMSAQETSFVTLGQRVLANPLKVRMHYGHPDVFDRFWFLSRGGIS
KASRVINISEDIFAGFNCTLRGGNVTHHEYIQVGKGRDVGLNQISMFEAKVASGNGEQVLSRDVYRLGHRLDFF
RMLSFFYTTVGFFFNTMMVILTVYAFLWGRVYLALSGVEKSALADSTDTNAALGVILNQQFIIQLGLFTALPMIV
EWSLEEGFLLAIWNFIRMQIQLSAVFYTFSMGTRAHYFGRTILHGGAKYRATGRGFVVEHKGFTENYRLYARSH
FVKAIELGLILIVYASHSPIAKDSLIYIAMTITSWFLVISWIMAPFVFNPSGFDWLKTVYDFEDFMNWIWYQGRIS
TKSEQSWEKWWYEEQDHLRNTGKAGLFVEIILVLRFFFFQYGIVYQLKIANGSTSLFVYLFSWIYIFAIFVLFLVIQY
ARDKYSAKAHIRYRLVQFLLIVLAILVIVALLEFTHFSFIDIFTSLLAFIPTGWGILLIAQTQRKWLKNYTIFWNAVVS
VARMYDILFGILIMVPVAFLSWMPGFQSMQTRILFNEAFSRGLRIMQIVTGKKSKGDV

>AT1G05570/AtCALS1
MAQRREPDPPPPQRRILRTQTVGSLGEAMLDSEVVPSSLVEIAPILRVANEVEASNPRVAYLCRFYAFEKAHRLD
PTSSGRGVRQFKTALLQRLERENETTLAGRQKSDAREMQSFYQHYYKKYIQALLNAADKADRAQLTKAYQTAA
VLFEVLKAVNQTEDVEVADEILETHNKVEEKTQIYVPYNILPLDPDSQNQAIMRLPEIQAAVAALRNTRGLPWTA
GHKKKLDEDILDWLQSMFGFQKDNVLNQREHLILLLANVHIRQFPKPDQQPKLDDRALTIVMKKLFRNYKKW
CKYLGRKSSLWLPTIQQEVQQRKLLYMGLYLLIWGEAANLRFMPECLCYIYHHMAFELYGMLAGSVSPMTGEH
VKPAYGGEDEAFLQKVVTPIYQTISKEAKRSRGGKSKHSVWRNYDDLNEYFWSIRCFRLGWPMRADADFFCQT
AEELRLERSEIKSNSGDRWMGKVNFVEIRSFWHIFRSFDRLWSFYILCLQAMIVIAWNGSGELSAIFQGDVFLKV
LSVFITAAILKLAQAVLDIALSWKARHSMSLYVKLRYVMKVGAAAVWVVVMAVTYAYSWKNASGFSQTIKNWF
GGHSHNSPSLFIVAILIYLSPNMLSALLFLFPFIRRYLERSDYKIMMLMMWWSQPRLYIGRGMHESALSLFKYTM
FWIVLLISKLAFSYYAEIKPLVGPTKDIMRIHISVYSWHEFFPHAKNNLGVVIALWSPVILVYFMDTQIWYAIVSTLV
GGLNGAFRRLGEIRTLGMLRSRFQSIPGAFNDCLVPQDNSDDTKKKRFRATFSRKFDQLPSSKDKEAARFAQM
WNKIISSFREEDLISDREMELLLVPYWSDPDLDLIRWPPFLLASKIPIALDMAKDSNGKDRELKKRLAVDSYMTC
AVRECYASFKNLINYLVVGEREGQVINDIFSKIDEHIEKETLITELNLSALPDLYGQFVRLIEYLLENREEDKDQIVIVL
LNMLELVTRDIMEEEVPSLLETAHNGSYVKYDVMTPLHQQRKYFSQLRFPVYSQTEAWKEKIKRLHLLLTVKESA
MDVPSNLEARRRLTFFSNSLFMDMPPAPKIRNMLSFSVLTPYFSEDVLFSIFGLEQQNEDGVSILFYLQKIFPDE
WTNFLERVKCGNEEELRAREDLEEELRLWASYRGQTLTKTVRGMMYYRKALELQAFLDMAKDEELLKGYKALE
LTSEEASKSGGSLWAQCQALADMKFTFVVSCQQYSIHKRSGDQRAKDILRLMTTYPSIRVAYIDEVEQTHKESYK

GTEEKIYYSALVKAAPQTKPMDSSESVQTLDQLIYRIKLPGPAILGEGKPENQNHAIIFTRGEGLQTIDMNQDNY
MEEAFKMRNLLQEFLEKHGGVRCPTILGLREHIFTGSVSSLAWFMSNQENSFVTIGQRVLASPLKVRFHYGHPD
IFDRLFHLTRGGICKASKVINLSEDIFAGFNSTLREGNVTHHEYIQVGKGRDVGLNQISMFEAKIANGNGEQTLSR
DLYRLGHRFDFFRMLSCYFTTIGFYFSTMLTVLTVYVFLYGRLYLVLSGLEEGLSSQRAFRNNKPLEAALASQSFVQ
IGFLMALPMMMEIGLERGFHNALIEFVLMQLQLASVFFTFQLGTKTHYYGRTLFHGGAEYRGTGRGFVVFHAK
FAENYRFYSRSHFVKGIELMILLLVYQIFGQSYRGVVTYILITVSIWFMVVTWLFAPFLFNPSGFEWQKIVDDWT
DWNKWIYNRGGIGVPPEKSWESWWEKELEHLRHSGVRGITLEIFLALRFFIFQYGLVYHLSTFKGKNQSFWVY
GASWFVILFILLIVKGLGVGRRRFSTNFQLLFRIIKGLVFLTFVAILITFLALPLITIKDLFICMLAFMPTGWGMLLIAQ
ACKPLIQQLGIWSSVRTLARGYEIVMGLLLFTPVAFLAWFPFVSEFQTRMLFNQAFSRGLQISRILGGQRKDRSS
KNKE

>AT3G14570/AtCALS8
MSHEIVPVDPIDVPSTSYSRPILGPREDSPERATEFTRSLTFREHVSSEPFDSERLPATLASEIQRFLRIANLVESEEP
RIAYLCRFHAFEIAHHMDRNSTGRGVRQFKTSLLQRLELDEEFTVRRRKEKSDVRELKRVYHAYKEYIIRHGAAFN
LDNSQREKLINARRIASVLYEVLKTVTSGAGPQAIADRESIRAKSEFYVPYNILPLDKGGVHQAIMHLPEIKAAVAI
VRNTRGLPPPEEFQRHQPFLDLFEFLQYAFGFQNGNVANQREHLILLLSNTIIRQPQKQSSAPKSGDEAVDALM
KKFFKNYTNWCKFLGRKNNIRLPYVKQEALQYKTLYIGLYLLIWGEASNLRFMPECLCYIFHHMAYELHGVLTGA
VSMITGEKVAPAYGGGHESFLADVVTPIYMVVQKEAEKNKNGTADHSMWRNYDDLNEFFWSLECFEIGWPM
RPEHDFFCVESSETSKPGRWRGMLRFRKQTKKTDEEIEDDEELGVLSEEQPKPTSRWLGKTNFVETRSFWQIFR
SFDRMWSFFVLSLQALIIMACHDVGSPLQVFNANIFEDVMSIFITSAILKLIKGILDIIFKWKARNTMPINEKKKRL
VKLGFAAMWTIILPVLYSHSRRKYICYFTNYKTWLGEWCFSPYMVAVTIYLTGSAIELVLFFVPAISKYIETSNHGIF
KTLSWWGQPRLYVGRGMQETQVSQFKYTFFWILVLLTKFAFSYAFEIKPLIEPTRLIMKVGVRNYEWHEIFPEVK
SNAAAIVAVWAPIMVVYFMDTQIWYSVYCTIFGGLYGVLHHLGEIRTLGMLRGRFHTLPSAFNASLIPHSTKDE
KRRKQRGFFPFNLGRGSDGQKNSMAKFVLVWNQVINSFRTEDLISNKELDLMTMPLSSEVLSGIIRWPIFLLAN
KFSTALSIAKDFVGKDEVLYRRIRKDEYMYYAVKECYESLKYILQILVVGDLEKKIISGIINEIEESIRQSSLLEEFKMAE
LPALHDKCIELVQLLVEGSAEQLQVEKSEELHGKLVKALQDIFELVTNDMMVHGDRILDLLQSREGSGEDTGIFM
RVIEPQLFESYGEWRCIHFPLPDSASLSEQIQRFLLLLTVKDSAMDIPENLDARRRLSFFATSLFMDMPDAPKVRN
MMSFSVLTPHYQEDINYSTNELHSTKSSVSIIFYMQKIFPDEWKNFLERMGCDNLDALKKEGKEEELRNWASFR
GQTLSRTVRGMMYCREALKLQAFLDMADDEDILEGYKDVERSNRPLAAQLDALADMKFTYVVSCQMFGAQK
SSGDPHAQDILDLMIKYPSLRVAYVEEREEIVLDVPKKVYYSILVKAVNGFDQEIYRVKLPGPPNIGEGKPENQNH
AIVFTRGEALQTIDMNQDHYLEEAFKMRNLLQEFLRNRGRRPPTILGLREHIFTGSVSSLAWFMSYQETSFVTIG
QRLLANPLRVRFHYGHPDVFDRIFHITRGGISKSSRTINLSEDVFAGYNTTLRRGCITYNEYLQVGKGRDVGLNQI
SKFEAKVANGNSEQTISRDIYRLGQRFDFFRMLSCYFTTIGFYFSSLISVIGIYIYLYGQLYLVLSGLQKTLILEAKVKN
IKSLETALASQSFIQLGLLTGLPMVMEIGLEKGFLIAFQDFILMQLQLAAFFFTFSLGTKTHYFGRTILHGGAKYRPT
GRKVVVFHANFSENYRLYSRSHFIKGFELMILLVVYELFKHTSQSNMAYSFITFSVWFMSFTWLCAPFLFNPSGF
TWEIIVGDWRDWNRWIKEQGGIGIQQDKSWQSWWNDEQAHLRGSGVGARCLEIILSLRFFVYQYGLVYHLDI
TQSNTNIIVYALSWVVILATFFTVKAVDLGRQLFSTRKHLVFRFFKVFVFVSILTIIITLANICHLSVKDLLVSCLAFLPT
GWGLILIAQAVRPKIEGTSLWEFTQVLARAYDYGMGVVLFAPMAILAWLPIISAFQTRFLFNEAFNRRLQIQPILA
GKKKNR

>AT2G13680/AtCALS5
MAQSSTSHDSGPQGLMRRPSRSAATTVSIEVFDHEVVPASLGTIAPILRVAAEIEHERPRVAYLCRFYAFEKAHRL
DPSSGGRGVRQFKTLLFQRLERDNASSLASRVKKTDGREVESFYQQYYEHYVRALDQGDQADRAQLGKAYQTA
GVLFEVLMAVNKSEKVEAVAPEIIAAARDVQEKNEIYAPYNILPLDSAGASQSVMQLEEVKAAVAALGNTRGLN

WPSGFEQHRKKTGNLDLLDWLRAMFGFQRDNVRNQREHLVCLFADNHIRLTPKPEPLNKLDDRAVDTVMSK
LFKNYKNWCKFLGRKHSLRLPQAAQDIQQRKILYMGLYLLIWGEAANIRFMPECLCYIFHNMAYELHGLLAGNV
SIVTGENIKPSYGGDDEAFLRKVITPIYRVVQTEANKNANGKAAHSDWSNYDDLNEYFWTPDCFSLGWPMRD
DGDLFKSTRDTTQGKKGSFRKAGRTGKSNFTETRTFWHIYHSFDRLWTFYLLALQAMIILAFERVELREILRKDVL
YALSSIFITAAFLRFLQSVLDVILNFPGFHRWKFTDVLRNILKIVVSLAWCVVLPLCYAQSVSFAPGKLKQWLSFLP
QVKGVPPLYIMAVALYLLPNVLAAIMFIFPMLRRWIENSDWHIFRLLLWWSQPRIYVGRGMHESQIALIKYTIF
WLLLFCCKFAFSYFLQVKLLVKPTNAIMSIRHVKYKWHEFFPNAEHNYGAVVSLWLPVILVYFMDTQIWYAIFSTI
CGGVIGAFDRLGEIRTLGMLRSRFQSLPGAFNTYLVPSDKTRRRGFSLSKRFAEVTAARRTEAAKFSQLWNEIISS
FREEDLISDREMDLLLVPYTSDPSLKLIQWPPFLLASKIPIALDMAAQFRTRDSDLWKRICADEYMKCAVIECYES
FKHVLHTLVIGENEKRIIGIIIKEVESNISKNSFLSNFRMAPLPALCSKFVELVGILKNADPAKRDTVVLLLQDMLEV
VTRDMMQNENRELVELGHTNKESGRQLFAGTDAKPAILFPPVATAQWHEQISRLHLLLTVKESAMDVPTNLEA
QRRIAFFTNSLFMDMPRAPRVRNMLSFSVLTPYYSEETVYSKNDLEMENEDGVSVVYYLQKIFPDEWTNFLERL
DCKDETSVLESEENILQLRHWVSLRGQTLFRTVRGMMYYRRALKLQAFLDMANETEILAGYKAISEPTEEDKKS
QRSLYTQLEAVADLKFTYVATCQNYGNQKRSGDRRATDILNLMVNNPSLRVAYIDEVEEREGGKVQKVFYSVLIK
AVDNLDQEIYRIKLPGPAKIGEGKPENQNHALIFTRGEALQAIDMNQDHYLEEALKMRNLLEEFNEDHGVRAP
TILGFREHIFTGSVSSLAWFMSNQETSFVTIGQRVLASPLKVRFHYGHPDVFDRIFHITRGGISKASRGINLSEDIF
AGFNSTLRRGNVTHHEYIQVGKGRDVGLNQISLFEAKVACGNGEQTLSRDLYRLGHRFDFFRMMSCYFTTVGF
YISSMIVVLTVYAFLYGRLYLSLSGVEEAIVKFAAAKGDSSLKAAMASQSVVQLGLLMTLPMVMEIGLERGFRTAL
SDLIIMQLQLAPVFFTFSLGTKVHYYGRTILHGGSKYRATGRGFVVKHEKFAENYRMYSRSHFVKGMELMVLLIC
YRIYGKAAEDSVGYALVMGSTWFLVGSWLFAPFFFNPSGFEWQKIVDDWDDWNKWISSRGGIGVPANKSWE
SWWEEEQEHLLHSGFFGKFWEIFLSLRYFIYQYGIVYQLNLTKESRMGKQHSIIVYGLSWLVIVAVMIVLKIVSMG
RKKFSADFQLMFRLLKLFLFIGSVVIVGMLFHFLKLTVGDIMQSLLAFLPTGWALLQISQVARPLMKTVGMWGS
VKALARGYEYIMGVVIFMPVTVLAWFPFVSEFQTRLLFNQAFSRGLQIQRILAGGKKQK

>AT3G59100/AtCALS6
MEASSSGTAELPRSLSRRAPSRATTMMIDRPNEDASAMDSELVPSSLASIAPILRVANEIEKDNPRVAYLCRFHAF
EKAHRMDATSSGRGVRQFKTYLLHRLEKEEEETKPQLAKNDPREIQAYYQNFYEKYIKEGETSRKPEEMARLYQI
ASVLYDVLKTVVPSPKVDYETRRYAEEVERKRDRYEHYNILPLYAVGTKPAIVELPEVKAAFSAVRNVRNLPRRRIH
LPSNTPNEMRKARTKLNDILEWLASEFGFQRGNVANQREHIILLLANADIRKRNDEEYDELKPSTVTELMDKTF
KSYYSWCKYLHSTSNLKFPDDCDKQQLQLIYISLYLLIWGEASNVRFMPECICYIFHNMANDVYGILFSNVEAVSG
ETYETEEVIDEESFLRTVITPIYQVIRNEAKRNKGGTASHSQWRNYDDLNEYFWSKKCFKIGWPLDLKADFFLNS
DEITPQDERLNQVTYGKSKPKTNFVEVRTFWNLFRDFDRMWIFLVMAFQAMVIVGWHGSGSLGDIFDKDVF
KTVLTIFITSAYLTLLQAALDIILNFNAWKNFKFSQILRYLLKFAVAFMWAVLLPIAYSKSVQRPTGVVKFFSTWTGD
WKDQSFYTYAVSFYVLPNILAALLFLVPPFRRAMECSDMRPIKVIMWWAQPKLYVGRGMHEDMFSLFKYTTF
WIMLLISKLAFNYYVEILPLITPTKMIMNLHIGHYQWHEFFPHATNNIGVVIAIWAPIVLVYLMDTQIWYAIFSTL
FGGIHGAFSHLGEIRTLGMLRSRFESIPIAFSRTLMPSEDAKRKHADDYVDQKNITNFSQVWNEFIYSMRSEDKI
SDRDRDLLLVPSSSGDVSVIQWPPFLLASKIPIAVDMAKDFKGKEDAELFRKIKSDSYMYYAVIESYETLKKIIYALL
EDEADRRVMNQVFLEVDMSMQQQRFIYEFRMSGLPLLSDKLEKFLSILLSDYEDQGTYKSQLINVFQDVIEIITQ
DLLVNGHEILERARVHSPDIKNEKKEQRFEKINIHLVRDRCWREKVIRLHLLLSVKESAINVPQNLEARRRITFFAN
SLFMNMPSAPRIRDMLSFSVLTPYYKEDVLYSEEDLNKENEDGISILFYLQKIYPDEWTNYLDRLKDPKLPEKDKS
EFLREWVSYRGQTLARTVRGMMYYRQALELQCYQEVAGEQAEFSVFRAMASNDENQKAFLERARALADLKF
TYVVSCQVYGNQKKSGDIHNRSCYTNILQLMLKYPSLRVAYVDEREETADAKSPKVFYSVLLKGGDKFDEEIYRIK
LPGPPAEIGEGKPENQNHAIIFTRGEALQTIDMNQDNYFEEAFKLRNVLEEFNKERVGRRKPTILGLREHIFTGSV
SSLAWFMSNQESSFVTIGQRILANPLRVRFHYGHPDIFDRIFHITRGGVSKASKVINLSEDIFGGFNSTLRGGYVT

HHEYIQVGKGRDVGLNPISIFEAKVANGNGEQTLSRDVYRLGHRFDFYRMLSFYFTTIGFYFSSMLTVLTVYAFLY
GRMYMVMSGLEKEILRLASPNQLEALEQALATQSIFQLGFLMVLPMVMEIGLEHGFRSAIVDFFIMQLQLASV
FFTFQLGTKSHYYGRTILHGGSKYRPTGRGFVVFHAKFAENYRLYSRSHFVKGLELLLLLVVYQIYGHSYRSSNLYLY
ITVSMWFMVGSWLFAPFIFNPSGFEWQKTVDDWTDWKRWLGDRGGIGIPVEKSWESWWNVEQEHLKHTS
IRGRILEITLALRFFIYQYGIVYQLNISQRSKSFLVYGLSWVVLLTSLLVLKMVSMGRRRFGTDFQLMFRILKALLFL
GFLSVMTILFVVFKLTLTDLSASVLAFLPTGWAILLIGQVLRSPIKALGVWDSVKELGRAYENIMGLVIFAPIAVLS
WFPIVSEFQARLLFNQAFSRGLQISMILAGRKDKATSSHK

>AT5G36870/AtCALS4
MNQPNRGQILQTVFSHFFPVASPDSELVPSSLHEDITPILRVAKDVEDTNPRSLFLQDLDIKSVDDSINILSGHSH
ALDKANELDPTSSGRDVRQFKNTILQWLEKNNESTLKARQKSSDAHEMQSFYQQYGDEGINDLLNAGAGSSSS
QRTKIYQTAVVLYDVLDAVHRKANIKVAAKILESHAEVEAKNKIYVPYNILPLDPDSKNHAMMRDPKIVAVLKAIR
YTSDLTWQIGHKINDDEDVLDWLKTMFRFQKDNVSNQREHLILLLANVQMRQTQRQPNLLDDRALDTVMEK
LLGNYNKWCNHVGLESSLRFPKDKQQKVVQQRKLLYTGLYLLIWGEAANLRFMPECLCYIYHHMAFELFEMLE
SKGSKKKYKPKNPTYSGKDEDFLTKVVTPVYKTIAEEAKKSGEGKHSEWRNYDDLNEYFWSKQYLDKLGWPMK
ANADFFCKTSQQLGLNKSEKKPDLGDGCVGKVNFVEIRTFWHLFRSFDRMWSFYILSLQAMIIIAWNETSESG
GAVFHKVLSVFITAAKLNLFQAFLDIALSWKARHSMSTHVRQRYIFKAVAAAVWVLLMPLTYAYSHTSIFIVAILIY
LSPNMLPEMLLLIPSIRRTLEKSDFRPVKLIMWWSQPELYIGRGMHESAWSIYKYMMFWIVLLTSKLAFSYYVE
QIKPLMGPTKEIMSVPMPGYWLPEFFPHVKNNRGVVITLWSPVILVYFMDTQIWYAIVSTLVGGLYGAFRHIGE
IQTLGMLRSRFQSLPGAFNACLIPNENTKEKGIKLAFSRKCHKIPNTNGKEAKQFSQMWNTIINSFREEDLISNRE
LELLLMSCWAYPDLDFIRWPIFLLASKIPIAVDIAKKRNGKHRELKNILAEDNCMSCAVRECYASIKKLLNTLVTGN
SDLMLITTVFTIIDTHIEKDTLLTELNLSVLPDLHGHFVKLTEYVLQNKDKDKIQIVNVLLKILEMVTKDILKEEIKRL
HLLLTVKESAMDVPSNLEARRRLTFFSNSLFMEMPGAPKIQNMLSFSALTPYYSEDVLFSTFDLEKENDGVSILFY
LQKIFPDEWKNFLERVKCGTEEELDAIDYLKEEIRLWASYRGQTLTKTVRGMMYYQKALELQAFFDLANERELM
KGYKSAEASSSGSSLWAECQALADIKFTYVVACQQYSIHKRSGDQRAKDILTLMTTYPSLRVAYIDEVEQTHIYSK
GTSENFYYSALVKAAPQTYSTDSSDSGHMLDQVIYQIKLPGPPIIGEGKPENQNNAIIFTRGEALQTIDMNQDYY
IEEAFKMRNLLQEFLEKNGGVRYPTILGLREHIFTRSVSCLAWFMSNQEHSFVTIGQRVLANPLKVRFHYGHPD
VFDRVFHLTRGGVSKASKVINLSEDIFAGFNSTLREGTVSHHEYIQVGKGRDVGLNQISMFEAKIANGSGEQTLS
RDLYRLGHQFDFFRMLSCYFTTVGFYFCSMLTVLTVYVFLYGRLYLVLSGVEKELGNKPMMMEIILASQSFVQIVF
LMAMPMIMEIGLERGFYDALFDFVLMQLQLASVFFTFQLGTKFHYYCKTLLHGGAEYRGTGRGFVVFHAKFAE
NYRFYSRSHFVKATELGILLLVYHIFGPTYIGLFTISIWFMVGTWLFAPFLFNPSGFEWHEIVEDWADWKKWIEY
DNGGIGVPPEKSWESWWEKDIEHLQHSGKWGIVVEIFFALRFFIFQYGLVYQLSAFKNKYSSLWVFGASWLLILI
LLLTVTVLDYARRRLGTEFQLLFRIIKVSLFLAFMAIFITLMTCRLILPQDVFLCMLALIPTGWGLLLIAQSCKPLIQQ
PGIWSWVMTLAWVYDLVMGSLLFIPIAFMAWFPFISEFQTRMLFNQAFSRGLHISRILSGQRKHRSSKNKD

>AT3G07160/AtCALS9
MSRAESSWERLVNAALRRDRTGGVAGGNQSSIVGYVPSSLSNNRDIDAILRAADEIQDEDPNIARILCEHGYSL
AQNLDPNSEGRGVLQFKTGLMSVIKQKLAKREVGTIDRSQDILRLQEFYRLYREKNNVDTLKEEEKQLRESGAFT
DELERKTVKRKRVFATLKVLGSVLEQLAKEIPEELKHVIDSDAAMSEDTIAYNIIPLDAPVTTNATTTFPEVQAAVA
ALKYFPGLPKLPPDFPIPATRTADMLDFLHYIFGFQKDSVSNQREHIVLLLANEQSRLNIPEETEPKLDDAAVRKVF
LKSLENYIKWCDYLCIQPAWSNLEAINGDKKLLFLSLYFLIWGEAANIRFLPECLCYIFHHMVREMDEILRQQVAR
PAESCMPVDSRGSDDGVSFLDHVIAPLYGVVSAEAFNNDNGRAPHSAWRNYDDFNEYFWSLHSFELGWPW
RTSSSFFQKPIPRKKLKTGRAKHRGKTSFVEHRTFLHLYHSFHRLWIFLAMMFQALAIIAFNKDDLTSRKTLLQILS
LGPTFVVMKFSESVLEVIMMYGAYSTTRRLAVSRIFLRFIWFGLASVFISFLYVKSLKAPNSDSPIVQLYLIVIAIYGG

VQFFFSILMRIPTCHNIANKCDRWPVIRFFKWMRQERHYVGRGMYERTSDFIKYLLFWLVVLSAKFSFAYFLQIK
PLVGPTRMIVKQNNIPYSWHDFVSRKNYNALTVASLWAPVVAIYLLDIHIFYTIFSAFLGFLLGARDRLGEIRSLEAI
HKLFEEFPGAFMRALHVPLTNRTSDTSHQTVDKKNKVDAAHFAPFWNQIIKSLREEDYITDFEMELLLMPKNSG
RLELVQWPLFLLSSKILLAKEIAAESNSQEEILERIERDDYMKYAVEEVYHTLKLVLTETLEAEGRLWVERIYEDIQTS
LKERNIHHDFQLNKLSLVITRVTALLGILKENETPEHAKGAIKALQDLYDVMRLDILTFNMRGHYETWNLLTQAW
NEGRLFTKLKWPKDPELKALVKRLYSLFTIKDSAAHVPRNLEARRRLQFFTNSLFMDVPPPKSVRKMLSFSVFTP
YYSEVVLYSMAELTKRNEDGISILFYLQKIYPDEWKNFLARIGRDENALEGDLDNERDILELRFWASYRGQTLART
VRGMMYYRKALMLQSYLERKAGNDATDAEGFELSPEARAQADLKFTYVVTCQIYGRQKEDQKPEAVDIALLM
QRNEALRIAYIDVVDSPKEGKSHTEYYSKLVKADISGKDKEIYSIKLPGDPKLGEGKPENQNHAIVFTRGNAIQTID
MNQDNYFEEALKMRNLLEEFDRDHGIRPPTILGVREHVFTGSVSSLASFMSNQETSFVTLGQRVLAKPLKIRM
HYGHPDVFDRVFHITRGGISKASRVINISEDIFAGFNTTLRQGNVTHHEYIQVGKGRDVGLNQIALFEGKVAGG
NGEQVLSRDVYRLGQLLDFFRMMSFFFTTVGFYLCTMLTVLTVYIFLYGRAYLALSGVGATIRERAILLDDTALSA
ALNAQFLFQIGVFTAVPMVLGFILEQGFLQAIVSFITMQFQLCTVFFTFSLGTRTHYFGRTILHGGARYQATGRGF
VVKHIKFSENYRLYSRSHFVKAMEVILLLVVYLAYGNDEAGAVSYILLTVSSWFLAVSWLFAPYLFNPAGFEWQKV
VEDFKEWTNWLFYRGGIGVKGAESWEAWWEEELSHIRTLSGRIMETILSLRFFIFQYGIVYKLKLQGSDTSFAVY
GWSWVAFAMIIVLFKVFTFSQKISVNFQLLLRFIQGLSLLMALAGIIVAVVLTPLSVTDIFACVLAFIPTGWGILSIA
CAWKPVLKRMGMWKSIRSLARLYDALMGMLIFLPVALCSWFPFVSTFQTRMMFNQAFSRGLEISLILAGDNP
NSGL

>AT2G31960/AtCALS2
MAQRKGPDPPPPQRRILRTQTAGNLGEAMLDSEVVPSSLVEIAPILRVANEVEASNPRVAYLCRFYAFEKAHRLD
PTSSGRGVRQFKTALLQRLERENETTLAGRQKSDAREMQSFYQHYYKKYIQALQNAADKADRAQLTKAYQTAA
VLFEVLKAVNQTEDVEVADEILEAHTKVEEKSQIYVPYNILPLDPDSQNQAIMRFPEIQATVSALRNTRGLPWPA
GHKKKLDEDMLDWLQTMFGFQKDNVSNQREHLILLLANVHIRQFPRPEQQPRLDDRALTIVMKKLFKNYKK
WCKYLGRKSSLWLPTIQQEVQQRKLLYMGLYLLIWGEAANLRFLPECLCYIYHHMAFELYGMLAGSVSPMTGE
HVKPAYGGEDEAFLQKVVTPIYKTIAKEAKRSRGGKSKHSEWRNYDDLNEYFWSIRCFRLGWPMRADADFFCQ
TAEELRLDRSENKPKTGDRWMGKVNFVEIRSFWHIFRSFDRMWSFYILSLQAMIIIAWNGSGKLSGIFQGDVFL
KVLSIFITAAILKLAQAVLDIALSWKSRHSMSFHVKLRFIFKAVAAAIWVVLMPLTYAYSWKTPSGFAETIKNWFG
GHQNSSPSFFIIVILIYLSPNMLSTLLFAFPFIRRYLERSDYKIVMLMMWWSQPRLYIGRGMHESALSLFKYTMF
WVVLLISKLAFSFYAEIKPLVKPTKDIMRVHISVYRWHEFFPHAKSNMGVVIALWSPVILVYFMDTQIWYAIVSTL
VGGLNGAFRRLGEIRTLGMLRSRFQSLPEAFNACLVPNEKSETPKKKGIMATFTRKFDQVPSSKDKEAARFAQM
WNKIISSFREEDLISDREMELLLVPYWADRDLDLIRWPPFLLASKIPIALDMAKDSNGKDRELTKRLSVDSYMTC
AVRECYASFKNLINFLVVGEREGQVINEIFSRIDEHIEKETLIKDLNLSALPDLYGQFVRLIEYLMENREEDKDQIVIV
LLNMLEVVTRDIMDEEVPSMLESTHNGTYVKYDVMTPLHQQRKYFSQLRFPVYSQTEAWKEKIKRLHLLLTVK
ESAMDVPSNLEARRRLTFFSNSLFMEMPDAPKIRNMLSFSVLTPYYSEDVLFSIFGLEKQNEDGVSILFYLQKIFP
DEWTNFLERVKCGSEEELRAREELEEELRLWASYRGQTLTKTVRGMMYYRKALELQAFLDMAKDEELMKGYKA
LELTSEDASKSGTSLWAQCQALADMKFTFVVSCQQYSVQKRSGDQRAKDILRLMTTYPSLRVAYIDEVEQTHKE
SYKGADEKIYYSALVKAAPQTKSMDSSESVQTLDQVIYRIKLPGPAILGEGKPENQNHSIIFTRGEGLQTIDMNQD
NYMEEAFKMRNLLQEFLVKHGGVRTPTILGLREHIFTGSVSSLAWFMSNQENSFVTIGQRVLASPLKVRFHYGH
PDVFDRLFHLTRGGVCKASKVINLSEDIFAGFNSTLREGNVTHHEYIQVGKGRDVGLNQISMFEAKIANGNGEQ
TLSRDLYRLGHRFDFFRMLSCYFTTIGFYFSTMLTVLTVYVFLYGRLYLVLSGLEEGLSNQKAFRSNMPLQAALAS
QSFVQIGFLMALPMMMEIGLERGFHNALIDFVLMQLQLASVFFTFQLGTKTHYYGRTLFHGGAEYRGTGRGF
VVFHAKFAENYRFYSRSHFVKGIELMILLLVYQIFGHAYRGVVTYILITVSIWFMVVTWLFAPFLFNPSGFEWQKI
VDDWTDWNKWIYNRGGIGVPPEKSWESWWEKEIGHLRHSGKRGIILEIVLALRFFIFQYGLVYQLSTFKQENQ

SLWIYGASWFVILFILLIVKGLGVGRQRFSTNFQLLFRIIKGFVFLTFLGLLITFLALRFLTPKDIFLCMLAFMPTGWG
MLLIAQACKPLIQRLGFWSSVRTLARGYEILMGLLLFTPVAFLAWFPFVSEFQTRMLFNQAFSRGLQISRILGGQ
RKDRSSKNKE

>AT2G36850/AtCALS10
MARVYSNWDRLVRATLRREQLRNTGQGHERVSSGLAGAVPPSLGRATNIDAILQAADEIQSEDPSVARILCEQA
YSMAQNLDPNSDGRGVLQFKTGLMSVIKQKLAKRDGASIDRDRDIERLWEFYKLYKRRHRVDDIQKEEQKWR
ESGTTFSSNVGEILKMRKVFATLRALIEVLEVLSRDADPNGVGRSIRDELGRIKKADATLSAELTPYNIVPLEAQSM
TNAIGVFPEVRGAVQAIRYTEHFPRLPVDFEISGQRDADMFDLLEYIFGFQRDNVRNQREHLVLTLSNAQSQLSI
PGQNDPKIDENAVNEVFLKVLDNYIKWCKYLRIRVVYNKLEAIDRDRKLFLVSLYFLIWGEAANVRFLPECICYIFH
NMAKELDAKLDHGEAVRADSCLTGTDTGSVSFLERIICPIYETISAETVRNNGGKAAHSEWRNYDDFNEYFWTP
ACFELSWPMKTESRFLSKPKGRKRTAKSSFVEHRTYLHLFRSFIRLWIFMFIMFQSLTIIAFRNEHLNIETFKILLSA
GPTYAIMNFIECLLDVVLMYGAYSMARGMAISRLVIRFLWWGLGSAFVVYYYVKVLDERNKPNQNEFFFHLYIL
VLGCYAAVRLIFGLLVKLPACHALSEMSDQSFFQFFKWIYQERYFVGRGLFENLSDYCRYVAFWLVVLASKFTFAY
FLQIKPLVKPTNTIIHLPPFQYSWHDIVSKSNDHALTIVSLWAPVLAIYLMDIHIWYTLLSAIIGGVMGAKARLGEI
RTIEMVHKRFESFPEAFAQNLVSPVVKRVPLGQHASQDGQDMNKAYAAMFSPFWNEIIKSLREEDYLSNREM
DLLSIPSNTGSLRLVQWPLFLLCSKILVAIDLAMECKETQEVLWRQICDDEYMAYAVQECYYSVEKILNSMVNDE
GRRWVERIFLEISNSIEQGSLAITLNLKKLQLVVSRFTALTGLLIRNETPDLAKGAAKAMFDFYEVVTHDLLSHDLR
EQLDTWNILARARNEGRLFSRIAWPRDPEIIEQVKRLHLLLTVKDAAANVPKNLEARRRLEFFTNSLFMDMPQA
RPVAEMVPFSVFTPYYSETVLYSSSELRSENEDGISILFYLQKIFPDEWENFLERIGRSESTGDADLQASSTDALELR
FWVSYRGQTLARTVRGMMYYRRALMLQSFLERRGLGVDDASLTNMPRGFESSIEARAQADLKFTYVVSCQIY
GQQKQQKKPEATDIGLLLQRYEALRVAFIHSEDVGNGDGGSGGKKEFYSKLVKADIHGKDEEIYSIKLPGDPKLG
EGKPENQNHAIVFTRGEAIQTIDMNQDNYLEEAIKMRNLLEEFHGKHGIRRPTILGVREHVFTGSVSSLAWFM
SNQETSFVTLGQRVLAYPLKVRMHYGHPDVFDRIFHITRGGISKASRVINISEDIYAGFNSTLRQGNITHHEYIQV
GKGRDVGLNQIALFEGKVAGGNGEQVLSRDVYRIGQLFDFFRMMSFYFTTVGFYVCTMMTVLTVYVFLYGRV
YLAFSGADRAISRVAKLSGNTALDAALNAQFLVQIGIFTAVPMVMGFILELGLLKAIFSFITMQFQLCSVFFTFSLG
TRTHYFGRTILHGGAKYRATGRGFVVQHIKFADNYRLYSRSHFVKAFEVALLLIIYIAYGYTDGGASSFVLLTISSWF
LVISWLFAPYIFNPSGFEWQKTVEDFEDWVSWLMYKGGVGVKGELSWESWWEEEQAHIQTLRGRILETILSLR
FFMFQYGIVYKLDLTRKNTSLALYGYSWVVLVVIVFLFKLFWYSPRKSSNILLALRFLQGVASITFIALIVVAIAMTD
LSIPDMFACVLGFIPTGWALLSLAITWKQVLRVLGLWETVREFGRIYDAAMGMLIFSPIALLSWFPFISTFQSRLL
FNQAFSRGLEISIILAGNRANVET

2. BoCALS

>BolC01g053890.2J/BoCALS1
MSDTTLVKMSRVESLWERLVNAALRRDGTRESAGGGGPGHGRDIDAILRAADELQFDDPIIARILCEHAHSLAQ
KLDPNSEGISVLQFRTGLMSVIKKKNEKREVETINRSQDINRLKGFYQRYREKNNVDTLKEEEKQLCESGAFTDEL
EQKTVERKRVFANLKVLEHVLEQVSKEIFEELKHANDSDAAMSHEDTVAYNIVPFDAPFTANATTAFPEVQAAVT
ALKYFPGLPKLPAEFPIPATRNADMLDFLHYIFGFQRDSVSNQREHIVLLLSNEQSHLNIPGETEHKLDDAAVSNV
FLKSLDNYIKWCDYLCIQPSWRNLEAISGEKKLLYLSLYFLIWGEAANIRFLPECLCYIFHHMVREMDEILRQQVA
RPAESCRPVDSSGSEDDVSFLVHVIAPLYEVVSAEAFNSGNGGAPHSSWRNYDDFNEYFWSLQSFELGWPWR
TSSSFFQKPRPRAKFELKTGREKHRGKTSFVEHRTFWHLYHSFHRLWIFLAMTFQALAIVAFNEKRLASRKALREI
LSLGPTYVVMKFSKSVLDVIMMYGAYSTTRRLAVYRMFLRLIWFGLASIFISFLYVRALQEDSKPNSDSVVFTLYVI

VIAIYGGVQFFFGILMRIPACHNIANKCEGWTVVRFFKWMWRQERHYVGSGMYEKTSDFIKYLLFWLVVLSAK
FSFAYFLQIEPLVSPTRMIVKHGNYNALTVASLWAPVVSIYLLDIHVFYTLVSALLGFFLGVRDRLGKIRSLEEIHKQF
NKFPGAFMRALHVPITDRSCDPSHEVVDKRNRVEASHFAPFWNQIIKCLREEDYITDFEMDLLLMPKNYGSRLV
QWPLFLLSSKILLATEIAAESKSQEEIVKRIEKDVYMKYAVEEVYYSLERVLITTLEAEGKIWVDRIFRDIRTSITMRTI
HLDFTLKKLSLVITRVTALLGVLKENETPENAAAATKALQDLYDVMRLDILAFDMRGHYDTWNVLTRASNEGRL
FTKLQWPKDPEMKALVKRLYSLLTIKDSTATHIPRNIEARRRLQFFTNSLFMDVPQPKPVHQMSSFSVFTPYCAE
VVLYSMAELTKRNEDGISILFYLQKIYPDEWKNFLARIGQDENALEGDLHNERDILELRFWASYRGQTLARTVRG
MMYYRKALMLQSYLERKGGRGISLYPVTDEESTLYGNDPTDAEGFELSPEARAQADLKFTYVVTCQMYGRQKE
DQKPEAADIALLMQRNEALRIAYIDVVDTLKEGKSHKEYYSKLVKADIDGKDKEIYSIRLPGDPKLGQGKAENLN
HAIVFTRGNAVQTIDMNQDNYFEEALKMRNLLEEFDRDHGIRPPTILGVREHVFTGSVSSLASFMSGQETSFVT
LGQRVLAKPLKIRMHYGHSDVFDRVFHITRGGISKASRVINASEDIFAGFNSTLRQGNITHHEYIQLGKGRYVGL
NQIALFEGKVAGGNGEQVLSRDVYRLGQLLDFFRMMSFYFTTVGFYVCTMLTVLTLYIFLYGKAYLALVSFTTMQ
FQLCTVFFTFSLGTRAHYFGRTLLHGGASVCQQYQATGRGFVVEHIKFSENYRLYSRSHFVKGMEVILLLVVYLAY
GNDEAGSVSYILLIVSSCFLAFSWIFAPYLFNPSGFERQKVVEDFKEWTKWLFYRGGGIGVEGAESWEAWWEE
ELVKNSISRLLLTLEKSGKLISYQQSHIGTLSGRMMETILSLRFFIFQYGIVYKLNVHGSDTSFAVYGWSWAAFAMI
LVLFKVFAFIQKIAISFRLVRRFIQGLALLVSLAGIIVAVVLTELSVTDIFACVLAFLPTGWGILSIACAWKPTIKRIGM
WESVRSLARLYDAGMGMLIFLPVALFSLFPFVSLPSNTCDEDKAKIKLHVTLLMMVIRLVSL*

>BolC02g005380.2J/BoCALS2
MSATRGGPDQGPSQPQQRRIVRTQTAGNLGESFDSEVVPSSLVEIAPILRVANEVESSNPRVAYLCRFYAFEKAH
RLDPTSSGRGVRQFKTALLQRLEREHDPTLMGRVKKSDAREMQSFYQHYYKKYIQALQNAADKADRAQLTKAY
QTANVLFEVLKAVNLTQSIEVDREILEAQDKVAEKTQLYVHYNILPLDPDSANQAIMRYPEIQAAVLGLRNTRGLP
WPEGHKKKKDEDMLDWLHEMFGFQKDNVANQREHLILLLANVHIRQFPKPDQQPKLDDQALTDVMKKLFK
NYKKWCKYLGRKSSLWLPTIQQEMQQRKLLYMALYLLIWGEAANLRFMPECLCYIYHHMAFELYGMLAGNVS
PMTGENVKPAYGGEEDAFLRKVVTPIYEVIAMESQRSKKGKSKHSQWRNYDDLNEYFWSVDCFRLGWPMRA
DADFFYPHVDQPNTEKDGDNSKPAVARDRWVGKVNFVEIRSFWHVFRSFDRMWSFYILCLQAMIIMAWDG
GQPSSVFGADVIKKVLSVFITAAIMKLGQATLDVILSFKAHRSMSLHVKLRYILKVLSAAAWVIILPVTYAYSWKDP
PGFARTIKSWFGSAMHSPSLFIIAVVFYLAPNMLAGVFFLFPMLRRFLERSNYRIVMLMMWWSQPRLYVGRG
MHESAFSLFKYTMFWVSLIATKLTFSYYIEIKPLVAPTQAIMKARVTDFQWHEFFPRAKNNIGVVIALWAPIILVYF
MDSQIWYAIFSTIFGGIYGAFRRLGEIRTLGMLRSRFESLPGAFNDRLIPDGKNQQKKKGLRATLSHNFTEDKVP
VNKEREAARFAQLWNTIISSFREEDLISDREMDLLLVPYWADRDLDLIQWPPFLLASKIPIALDMAKDSNGKDRE
LKKRIESDSYMKCAVRECYASFKNVIKFLVQGNREKEVIEFIFTEVDEHIEKGDLIQVYKMSSLPSLYDHFVKLIKYLL
DNNVDDRDHVVILFQDMLEVVTRDIMMEDSISSLVDSSHGGAWHGGMVPLEQQYQLFASSGAIRFPIEPVTE
AWKEKIKRLYLLLTTKESAMDVPSNLEARRRISFFSNSLFMDMPAAPKVRNMLSFSVLTPYYTEEVLFSMHDLDT
PNEDGVSILFYLQKIFPDEWNNFLERVKSNEEEIKESVELEEELRLWASYRGQTLTRTVRGMMYYRKALELQAFL
DMAMHEDLMEGYKAVELNSENNSRGERSLWAQCQAVADMKFTYVVSCQQYGIHKRSGDPRAQDILRLMTR
YPSLRVAYIDEVEEPVKDKSKKGNEKVYYSVLVKVPKSTDHSNLAQNLDQVIYRIKLPGPAILGEGKPENQNHAIIF
SRGEGLQTIDMNQDNYMEEALKMRNLLEEFLTKHDGVRHPSILGLREHIFTGSVSSLAWFMSNQETSFVTIGQ
RLLANPLRVRFHYGHPDVFDRLFHLTRGGVSKASKVINLSEDIFAAGFNSTLREGNVTHHEYIQVGKGRDVGLN
QISMFEAKIANGNGEQTLSRDIYRLGHRFDFFRMMSCYFTTVGFYFSTLITVLTVYIFLYGRLYLVLSGLEQGLSTQ
KGIRDNTPLQIALASQSFVQIGFLMALPMLMEIGLERGFRTALSEFVLMQLQLAPVFFTFSLGTKTHYYGRTLLH
GGAKYRSTGRGFVVFHAKFADNYRLYSRSHFVKGLEMMLLLVVYQIFGSAYRGVVAYLLITISMWFMVGTWLF
APFLFNPSGFEWQKIVDDWTDWNKWINNIGGIGVPAEKSWESWWEEEQEHLRHSGKRGIVVEILLSLRFFIYQ
YGLVYHLTITEKTKNFMVYGVSWLVIFLILFVMKTVSVGRRKFSASFQLMFRLIKGLIFLTFIAIIVILITLAHMTIQDII

VCILAFMPTGWGMLLIAQACKPVVHRAGFWGSVRTLARGYEIVMGLLLFTPVAFLAWFPFVSEFQTRMLFNQ
AFSRGLQISRILGGHRKDRSSRNKE*

>BolC03g038840.2J/BoCALS3
MSLAESSWKRLVNAALQRDRAGGGAGGGTGQGSLMDYVPSSLPNNRDIDAILRAADELQNEDPSIARILCEH
AYSLAQNLDPKSEGRGVLQFKTGLMSVVKQKLAKREVGTIDRTQDSKRLQDFYRLYREKNNVDTLKEEEMQLRE
SGVLTGELERKTVKRKRLFATLKILGNVLEKVAKEIPDELKHVIDSDAAISEDTIAYNIIPLEAHVTTNAITGFPEVKA
AVAALKYFRGLPKLPDNFPIPATRNADMLDFLQYIFGFQKDSVSNQREHIVLLLANEQSRLNIPEEMEPKLDDGA
VHKVFLKSLDNYIKWCDYLCIKPAWSNLERISGEKKLLVLSLYFLIWGEAANIRFLPECLCYIFHHMVVEMNEILRQ
QVSRPAVSCMPVDSHGGSDVGVSFLDHVIAPLYEVVSAEAFHNENGRAPHSEWRNYDDFNEYFWSLRSFELG
WPWRTSSSFFQKPIRRRKVCMPPPLIIYINRILYSHWFVTSFVFILVCYFFFNILVLFLQYEFEAGRDKHRGKTSFVE
HRTFLRLYHSFHRLWIFLAIMFQTSLQALAIIAFNKNSIISTKTLRQILSLGPTYVLMKFSESLLDVIMMYGAYSTTR
RLAVSRIFLRLIWFSLACVFICFLYVKMLQEDTKPIYNSVMFKVYGLVIAIYGGIQLFFSILMHIPSCRNIASKCDRW
AVIRFVEGMRQERHYVGRDMYERMSDFIKYFLFWLVVLSAKFSFAYFLQIAPLVGPTRMIMKQDNIQYSWHEF
VSIKNYNALTVASLWAPVVAIYLLDIHIFYTIASAFFGFLLGARDRLGEIRSLEAIHKQFEEFPGAFMKALHVPITNR
LGSCPSKAVDKNIVDAAHFAPFWNQIIKCLREEDYITDLEMELLLMPKNSGRLQLVQWPLFLLSSKILLAKEIAAE
SNSQEEIVERIGKIDYMKYAVEEVYHTLKLVLTETLEAEGRMWVERIYEDIDTSIKNRKIHNDFQLNKLSLVITRVT
ALLGILKENETPEHAKGAIKALQDLYDVIRLDVLNFNMRGQYEMWNNLTQAWNEGRLFTELKWPKDPELKALV
RRLYSLFTIKDSAAHVPRNLEARRRLQFFTNSLFMDVPPPKSVDKIVFTPYYSEVVLYSMAELTKRNEDGISILFYL
QKIYPDEWKNFLARIGQDENALEGDLRNERDVLELRFWASYRGQTLARTVRGMMYYRKALMLQSYLERKAGR
DGGESTLFGNNMTDAEGFELSPEARAQADLKFTYVVTCQIYGRQKEDKKPEAVDIALLMQRNEALRIAYIDVVD
TPKEGISHTEYYSKLVKADINGKDKEIYSIRLPGDPKLGEGKPENQNHAIVFTRGNALQTIDMNQDNYFEEALKM
RNLLEEFDPNRDHGIRPPTILGVREHVFTGSVSSLASFMSNQETSFVTLGQRVLAKPLKIRMHYGHPDVFDRVF
HITRGGISKASRVINISEDIFAGFNSTLRQGNITHHEYIQVGKGRDVGLNQIALFEGKVAGGNGEQVLSRDVYRL
GQLLDFFRMLSFYFTTVGFYFSTMLTVLTLYIFLYGRAYLALSGVGATLRERAISLDDTALSAALNAQFLFQIGVFTA
VPMILGFILEQGFLKAIVSFTTMQFQLCTVFFTFSLGTRTHYFGRTILHGGARYQATGRGFVVKHITFSENYRLYSR
SHFVKGLEVILLLVVYLAYGNDKAGAVSYILLTVSSWFLAGSWLFAPFLFNPAGFEWQKVVVDFTEWTNWLFYR
GGIGVKGSESWEAWWEEELSHIRTLSGRIMETILSLRFFVFQYGIVYKLKLQGSDTSFSIYGWSWAALAIIIFLFKV
FTFSRKISVNFQLVLRLAQGIAFLVALAVVTVGVVLTDLSVTDIFASVLALIPTGWGILSIACAWKPIIKRIGMWKSI
RSLARLYDAGMGMLIFLPVAFCSWFPFLSTFQTRMMFNQAFSRGLEISLILAGNHPNSDL*

>BolC04g011790.2J/BoCALS4
MARVYSNWDRLVRATLRREQLRDSGQGHERVNSGLAGAVPPSLGRATNIDAILQAADEIQAEDPNVARILCEQ
AYSMAQNLDPNSDGRGVLQFKTGLMSVIKQKLAKRDGASINRDRDIERLWQFYQLYKRRHRVDDIQREEQKW
RESGTAFSSNVGEILKMRKVFATLRALVEVLELLSRDADPNGVGRSIREELGRIKKADATLSAELTPYNIVPLEAQS
MTNAISVFPEVRGAIQAIRYTEHFPKLPDDYEISGQRDADMFDLLEYIFGFQKDNVRNQREHLVLTLSNAQSQL
GLPSPNDPKIDEKAVNEVFLKVLDNYIKWCKYLRIRLVYNKLEAINRDRKLFLVSLYFLIWGEAANVRFLPECICYIF
HNMAKELDAKLDHGEAVRADSCLIENGSVSFLDRVISPIYAAMSAETLRNNNGKAAHSEWRNYDDFNEYFWT
PGCFELSWPMKTESKFLTGPKGRKRTGKSSFVEHRTYLHLFRSFHRLWIFMVIMFQALAIIAFRKEHLDKDTFKIL
LSAGATYAIMNFIESFLDVVLMYGAYSMARGMAISRVFIKFFWWGLGSVFVVYVYVQVLQERNKRSSDEFFYRL
YVLVLGIYAAVRLIFGLLVKLPACHALSEMSDQSFFQFFKWIYQERYFVGRGLFENMSDYCRYVAFWLIVLASKFT
FAYFLQARISNHLLNPPRRSLIFLRLNIHGMILSQKIYLMDIHIWYTLLSAIIGGVMGAKARLGEIRSIEMVHKRFES
FPEAFAKNLVSPVVKRVSFGQHASQDGQDMNKAYAAMFSPFWNEIIKSLREEDYISNREMDLLSIPSNTGSLGL
VQWPLFLLCSKILVAIDLAMECTETQGVLWRQICDDEYMAYAVQECYYSVQNILNSMVDGVGRRWVERVFME

ISNSIQEGSLAITLNLKKLQLVVSRFTALTGLLIRNETPDLAKGAAKAMFDFYEVVTHDLLAENLRDQLDTWNILA
RARNEGSLFSTIEWPRDPEIIEQVKRLHLLLTVKDAAANVPKNLEARRRLEFFTNSLFMDMPQARPVAEMVPFS
VFTPYYSETVIYSSSELRSENEDGISTLFYLQKIFPDEWENFLERIGRSDSTGDVDLQESATDALELRFWVSFRGQT
LARTVRGMMYYRRALMLQSFLERRGLGVDDVSLTNMPRGFEASPEARAQADLKFTYVVSCQIYGQQKQQKK
PEATDIALLLQRFEALRVAFIHSEDVGVEGKKEFYSKLVKADIHGKDQEIYSIKLPGDPKLGEGKPENQNHAIVFTR
GEAIQTIDMNQDNYLEEAIKMRNLLEEFHGKHGIRRPTILGVREHVFTGSVSSLAWFMSNQETSFVTLGQRVLA
YPLKVRMHYGHPDVFDRVFHITRGGISKASRVINISEDIYAGFNSTLRQGNITHHEYIQVGKGRDVGLNQIALFE
GKVAGGNGEQVLSRDVYRIGQLFDFFRMMSFYFTTVGFYVCTMVLSFSSVLYFCFLHYHVVKPFSLMQMTVLT
VYVFLYGRVYLAFSGSDRAISRVAKLSGNTALDAALNAQFLVQIGVFTAVPMVMGFILELGLLKAIFSFITMQFQL
CSVFFTFSLGTRTHYFGRTILHGGAKYRATGRGFVVQHIKFADNYRLYSRSHFVKAFEVALLLIVYIAYGYTDGGAV
SFVLLTISSWFLVISWLFAPYIFNPSGFEWQKTVEDFDNWVSWLMYKGGVGVKGELSWESWWEEEQMHIQTL
RGRILETILSLRFFMFQYGVVYKLNLTGKDTSLAIYGYSWIVLVAVVLLFKLFWYSPRKSSNILLALRFLQGVVSLTFI
ALIALAIALTDLSIPDMFACVLGFIPTGWAILSLAITWKRLIKLFGLWETVREFGRIYDAAMGMLIFAPIALLSWFPF
ISTFQSRLLFNQAFSRGLEISIILAGNRANVET*

>BolC04g017120.2J/BoCALS5
MSQRRGGGPDPPPPQRRILRTQTAGNLGEAMLDSEVVPSSLVEIAPILRVANEVEASNPRVAYLCRFYAFEKAH
RLDPTSSGRGVRQFKTALLQRLERENETTLAGRQKSDAREMQSFYQHYYKKYIQALLNAADKADRAQLTKAYQT
AAVLFEVLKAVNQTEDVEVADEILEAHTKVEEKSQIYVPYNILPLDPDSQNQAIMRFPEIQATVIALRNTRGLPW
PAGHKKKLDEDMLDWLQTMFGFQKDNVANQREHLILLLANVHIRQFPRPEQQPKLDDRALTIVMKKLFKNYK
KWCKYLGRKSSLWLPTIQQEVQQRKLLYMGLYLLIWGEAANLRFMPECLCYIYHHMAFELYGMLAGSVSPTTG
EHIKPAYGGDDEAFLQKVVTPIYKTIAKEAKRSRGGKSKHSEWRNYDDLNEYFWSVRCFRLGWPMRADADFFC
QTAEEHRLDRSEDKPKTGDRWMGKVNFVEIRSFWHIFRSFDRMWSFYILSLQAMIIIAWNGSGDLGAIFQGD
VFLKVLSIFITAAILKLAQAVLDIALSWKSRHSMSFHAKLRFILKAIAAAVWVVLMPVTYAYSWRSPSGIAETIKN
WLGGHSGSSPSLFIMVILIYLSPNMLSTLLFVFPFIRRYLERSDIKIVMLMMWWSQPRLYIARGMHESAWSLFKY
TMYWVVLLISKLAFSFYAEIKPLVVPTKDIMRVHISVYRWHEFFPHAKNNLGVVVALWSPVILVSWPQLPLTYAS
FLYIYANFHIQVYFMDTQIWYAIVSTLVGGLNGAFRRLGEIRTLGMLRSRFQSLPGAFNACLVPNEKSETAKKRGI
RATFSRKFDQIPSSKDKEAARFAQMWNKIISSFREEDLISNREMELLLVPYWADPDLDLIRWPPFLLASKIPIALD
MAKDSNGKDRELKKRLSIDSYMTCAVRECYASFRNLINFLVVGERERQVINEIFAKIDEYIANETLIETLDLSALPDL
YGQFVRLIEYLMENKEEDKDQIVIVLLNMLEVVTRDIMDYEVPSLLETAHNGTYVKYDVMTPLHQQKKYFSQLR
FPVYSQTEAWKEKAKDLLSYLFVFLFFIVQIKRLHLLLTVKESAMDVPSNLEARRRLTFFSNSLFMEMPDAPKIRN
MLSFSVLTPYYQEDVLFSIFGLEKQNEDGVSILFYLQKIFPDEWTNFLERIKCGSEEEIRAREDLEEELRLWASYRG
QTLTKTVRGMMYYRKALELQAFLDMAKDEELMKGYKALELTSEDASKTGKSLWAQCQALADMKFTFVVSCQ
QYSIQKRSGDQRAKDILRLMTTYVFLQLVCYFHSRMHELNVLFLCGRYPSLRVAYIDEVEQTHKDSYKGADDKIY
YSALVKAAPQTKPMDSSESVQTLDQVIYRIKLPGPAILGEGKPENQNHAIIFTRGEGLQTIDMNQDNYMEEAFK
MRNLLQEFLEKHGGVRTPTILGLREHIFTGSVSSLAWFMSNQENSFVTIGQRVLASPLKVRFHYGHPDVFDRLF
HLTRGGVCKASKVINLSEDIFAGTCFNSLLLVTRLLPLIVSSYLYFFSGFNSTLREGNVTHHEYIQVGKGRDVGLNQI
SMFEAKIANGNGEQTLSRDLYRLGHRFDFFRMLSCYFTTIGFYFSTMLTVLTVYVFLYGRLYLVLSGLEEGLSNQK
AFRSNMPLQAALASQSFVQIGFLMALPMMMEIGLERGFHNALIDFVLMQLQLASVFFTFQLGTKTHYYGRTLF
HGGAEYRGTGRGFVVFHAKFAENYRFYSRSHFVKGIELMILLLVYQIFGHAYRGVVTYILITVSIWFMVVTWLFA
PFLFNPSGFEWQKIVDDWTDWNKWIYNRGGIGVPPEKSWESWWEKEIGHLRHSGKRGIILEIVLALRFFIFQY
GLVYQLSTFKQENQSLWIYGASWFVILFILLIVKGLGMGRRRFSTNFQLLFRIIKGLVFLTFLAILITFIALRLLTPKDIL
LCMLAFMPTGWGMLLIAQACKPLIQRLGIWSSVRTLARGYEILMGLLLFTPVAFLAWFPFVSEFQTRMLFNQA
FSRGLQISRILGGQRKDRSSKNKE*

>BolC05g003830/BoCALS6
MSQRRESGPSRPHRPIQRTQTLGSLGEAMLDSEVVPSSLVEIAPILRVANEVEASNPRVAYLCRFYAFEKAHRLD
PTSSGRGVRQFKTALLQRLERENETTLAGRQKSDAREMQRFYQHYYEKYIHALNAADKADRAQLTKAYQTASVL
FEVLKAVNQTEDVPVPVKILQQQKKVEEKTQIYKPYNILPLDPDSQNQAIMRLPEIQAAVTALRNIRGLPWKAG
HKKKIDEDILDWLQSMFGFQEDSVSNQREHLILLLANVHIRQYPRPEQEPKLDDRALTIVMKKLFRNYKKWCKY
LGRKSSLWLPTIQQEVQQRKLLYMGLYLLIWGEAANLRFMPECLCYIYHHMAFELYGMLAGSVSSLTGEHVKPA
YGGDDEAFLQKVVTPIYKTIAKEAKRSRDGKSKHSVWRNYDDLNEYFWSIRCFRLGWPMRADADFFCLTAEEL
RVENSEIKSNSGDRWMGKVNFVEIRSFWHIFRSFDRMWSFYILCLQAMIVIAWNGSGELSAIFQGDVFLKVLSI
FITAAVLKLAQALLDIALSWKARHSMSHYVKLRYVLKAGAAAGWVIVMPVAYAYSWKNASGFALTIKNWFGGH
SHNSPSLFIVAILIYLSPNMLSALLFLFPFIRRYLERSDFKIMMLMMWWSQPRLYIGRGMHESALSLFKYTMFWI
VLLVSKLAFSFYAEIKPLVGPTKDIMRIHISVYSWHEFFPHAKNNLGVVIALWSPVILVYFMDTQIWYAIVSTLVGG
LNGAFRRLGEIRTLAMLRSRFQSIPGAFNDCLVPHDNSDDTKKRGFKATFSRKFDQLPSSKDKEAARFAQMWN
KIISSFREEDLISDREMELLLVPYWSDPDLDLIRWPPFLLASKIPIALDMAKDSNGKDRELKKRLAVDSYMTCAVS
ECYASFKNLINYLVIGERERQVINDIFSKIDEHIEKETLITELNLSSLPDLYGQFVQLIEYLIQNREEDKDQIVIVLLNM
LEVVTRDIMDEEVPSLLETAHNGAYVKYDVMTPLHQQRKYFSQLQFPVFSQKEAWKEKARLFSFICLILIKRLHLL
LTVKESAMDVPSNLEARRRLTFFSNSLFMDMPPAPKIRNMLSFSVLTPYFSEDVLFSIFGLEQQNEDGVSILFYLQ
KIFPDEWTNFLERVKCGSEEELRTKDDLEEELRLWASYRGQTLTKTVRGMMYYRKALELQAFLDMAKDEELLKG
YKALELTSEEASKSGESLWAQCQALADMKFTFVVSCQQYSIHKRSGDQRAKDILRLMTTYPSIRVAYIDEVEQTH
KESYKGTEEKIYYSALVKAAPQTKPMDSSESVQTLDQLIYRIKLPGPAILGEGKPENQNHAIIFTRGEGLQTIDMN
QDNYMEEAFKMRNLLQEFLVKHGGVRFPTILGLREHIFTGSVSSLAWFMSNQENSFVTIGQRVLASPLKIRFHY
GHPDIFDRLFHLTRGGICKASKVINLSEDIFAGFNSTLREGNVTHHEYIQVGKGRDVGLNQISMFEAKIANGNGE
QTLSRDLYRLGHRFDFFRMLSCYFTTIGFYFSTMLTVLTVYVFLYGRLYLVLSGLEQGLSNQRAFRNNRPLEAALAS
QSFVQIGFLMALPMMMEIGLERGFHNALIEFVLMQLQLASVFFTFQLGTKTHYYGRTLFHGGAEYRGTGRGFV
VFHAKFAENYRFYSRSHFVKGIELMILLLVYQLFGQSYRGVVTYILITVSIWFMVVTWLFAPFLFNPSGFEWQKIV
DDWTDWNKWIYNRGGIGVPAEKSWESWWEKELEHLKHSGVRGIVLEIFLALRFFIFQYGLVYQLSIFKGKNQSF
WVYGASWFVILFLLLIVKGLGMGRRRFSTSFQLLFRIIKGLVFLAFVTILITLLALPLITIKDLFICMLAFMPTGWGM
LLIAQACKPLIQHLRIWSSVRTLARGYEIVMGLLLFTPVAFLAWFPFVSEFQTRMLFNQAFSRGLQISRILGGHRK
DRSSKNKE*

>BolC05g004600.2J/BoCALS7
MASTGGGRGEDGRPPQMQPVRSLSRRMTRAGTMMIDHPNEDENVIDSELVPSSLASIAPVLRVANEIEDQN
PRVAYLCRFHAFEKAHRMDPTSSGRGVRQFKTYLLHKLEKEEPTSDPKEIQNYYQRFYEENIQKGEGRKTPEEM
AKLYQMASVLYDVLKTVVPPARIDEKTHRYAKEVERKKDHYEHYNILPLDAGGAKTAIMELPEIKVAIRAVSNVEN
LPRPRLRSSSTNPDQVEREETRSFNDILEWLALVFGFQRGNVANQREHLILLLANVDVRKRDLENYDELKPSTVR
KLMDKYFKNYRSWCKYLRCESFLRFPPGCNEQQLSLAYIGLYLLIWGEASNIRFMPECLCYIFHKMADEVHGILFS
NVYPVTGDTYEAGAPDDEAFLRNVITPIYQVLRKEVRRNKMGKASHSKWRNYDDLNEYFWDKRCFRLGWPM
KPEADFFIHTDEIAQHPNERRDQVPHGKRKPKTNFVEARTFWNLYRSFDRMWMFLVLSLQTMMIVAWSPSG
SILAIFEEDVFLNVLTIFITSAFLNLLQATLDIILSFGAWKSLKFSQILRFITKFLMAAMWAIILPIAYSKSVQNPTGLIK
FFSSWVQSWPHQTLYNYAIALYVLPNILAAVFFLLPPLRRIMERSNMRIVTLIMWWAQPKLYVGRGMHEEMFA
LFKYTFFWVMLLLSKLAFSFYVEILPLVKPTKLIWDMSGVNYQWHEFFPNATHNIGVIISIWGPIVLVYFMDTQI
WYAIFSTIFGGIYGAFSHLGEIRTLGMLRSRFRFVPSAFCSKLTPSPPGRAKRKHLDEQVDENDIARFSQMWNKF
IYTMRDEDLISDRERDLLLVPSSSGDVTVVQWPPFLLASKIPIALDMAKDFKGKEDAELFKKIKSEYYMYYAVVEA
YESMRDVIYGLLEDESDKRIVREICFEIDDSIQQHRFLTAFRMTGMPLLSDKLEKFLKILLSDYGEDETYKSQIINVL

QDIIEIITQDVMVKGHEILERAHYQSGDIENEKKEQRFEKINLGGQNDSWREKVVRLLLLVTVKESAINIPQNLEA
RRRMTFFANSLFMNMPDAPRVRDMLSFSVLTPYYKEDVLYSEEELNKENEDGISILFYLQRIYPGELPSGVFVRLY
NKFEVYRMKMICYTEEWSNFSERVNDPKRIFSEKDKTDQLREWVSYRGQTLSRTVRGMMYYRMALELQCFQ
EYTEYAAHSGYLPSASYDEFMNRARALADLKFTYVVSCQVYGNQKKSSDGRDRSCYNNILQLMLKYPSLRVAYI
DEREETINKKSQKVFYSVLLKGCNKLDEEIYRIKLPGNPTEIGEGKPENQNHAIIFTRGEALQTIDMNQDNYFEET
FKMRNVLQEFDEGRRGKRNPTILGLREHIFTGSVSSLAWFMSNQETSFVTIGQRVLANPLRVRFHYGHPDIFDR
IFHITRGGISKASKIINLSEDIFAGYNSTLRGGYITHHEYIQAGKGRDVGMNQVSVFEAKVANGNGEQTLSRDVY
RLGRRFDFYRMLSFYFTTVGFYFSSMITVLTVYVFLYGRLYLVLSGLEKEILQSATVHQSKALEEALAAQSVFQLGFL
MVLPMVMEIGLEKGFRKALGDFIIMQLQLASVFFTFQLGTKAHYFGRTILHGGSKYRATGRGFVVFHAKFADNY
RLYSRSHFVKGLELVMLLIVYQVYGNSYRSSSLYIYITFSMWFLVTSWLFAPFIFNPSGFEWQKTVDDWTDWKR
WMGNRGGIGIVVEKSWESWWESEQEHLKHTNLRGRVLEILLALRFLLYQYGIVYHLNVAHRDTTILVYGLSWG
VLLAVLLVLKMVSMGRRKFGTDFQVMFRILKALLFLGFLSVMTVLFVVCGLTIADVCASFLAFLPTGWAILLIGQA
LRGVLKGLGFWDSIKELGRAYEYIMGLSIFTPIAVLSWFPFVSEFQTRLLFNQAFSRGLQISMILAGKKDKETPAPS
K*

>BolC05g051770.2J/BoCALS8
MSHEIVPVDPIDVPSTSYSRPNLGREDSPERHQHQYTRSLTFREHVSEPFDSERLPATLASEIQRFLRIANLVESEE
SRIAYLCRFHAFEIAHHMDRNSTGRGDEEFTVIRRKEKSDVRELKRVYHAYKEYIIKHGATFNLDHSQREKLVNAR
RIASVLYEVLKTVTSGAGPQAIADRESIRAKSEFYVPYNILPLDRGGVHQAIMHLPEIKAAVAIVRNTRGLPPPEDF
QRHQPFVDLFEFLQYAFGFQSGNVANQREHLILLLSNTIIRQPQKQSSQPKSGDEAVDALMKKFFKNYTSWCKF
LGRKNNIRLPCVKKEALQYKTLYIGLYLLIWGEASNLRFMPECLCYIFHQMAYELHGVLAGDVSMITGEKVVPAYR
GVSHETFLEKVVTPIYKVIEKEAEKNKNGTADHSMWRNYDDLNEFFWSIECFELGWPMRSEHDFFCVEQLDTS
KPRRWREKLRLRRQTKKTDEEMEDDEELGPITEEQTKPTQRWLGKTNFVEIRSFWQIFRSFDRMWSFFVLSLQ
ALIIMACHDVESPLQIFNANIFEDVMSIFITSAFLKLIKGILDIIFKWKTRTTMPINEKKKQMARLGLAAMWTVILP
VLYSHSRRKYICYFTSYKTWLGEWCFSPYMVAVTIYLTGSAVELVLFFVPAISKYIETSNHRIFKTLYWWGQPRLFI
GRGVQETQISQFKYTLFWILVLLTKFAFSYAFEIKPLIEPTRLIMKVGVRNYEWHEIFPEVKSNAAAIVAVWAPIM
VVYFMDTQIWYSVFCTIFGGLYGVLHHLGEIRTLGMLRGRFHTLPSAFNASLIPHSIKDEKRRKQSGFFPFNFCR
GSDGQKNSMAKFVLVWNQVINSFRTEDLISNKELDLMTMPMSSEVLSGIIRWPIFLLANKFTTALSIAKDFKDK
DEALYRRIRRDEYMYYAVKECYESLKYILQILVVGDLEKKIISGIINEIEESIRQSSLLEEFKLKELPTLHKKCIELVQLLV
EGSEDKLPVEKIEEQHSKLVKALQDIFELVTNDMMVHGHRILDLLEPLEDSEEDTGIFMRVIEPQLFESYGERRCI
HFPLPDSTSLSEQIQRFLLLLTVKDSAMDIPENLDARRRISFFATSLFMDMPDAPKVRNMMSFSVLTPHYQEDIN
FSTKELHSANSSVSIIFYMQKIFPDEWKNFLERMGCENLDALKREGKEEELRNWASFRGQTLRRTGEKLQQYLQI
ALYRELTNFMLFSCMNTVRGMMYCRDALKLQAFLDMADDEDILEGYDDVERSNRPLAAQLDALADMKFTYV
VSCQMFGAQKSAGDPHAQDILDLMIKYPSLRVAYVEEREEIVSDNPEKVYYSILVKAVNGFDQEIYRVKLPGPPN
IGEGKPENQNHAIIFTRGEALQTIDMNQDNYLEEAFKMRNLLQEFLRNRGRRPPTILGLREHIFTGRVRFHYGH
PDVFDRIFHITRGGISKASRTINLSEDVFAGYNTTLRRGCITYNEYLQVGKGRDVGLNQISKFEAKVANGNSEQTI
SRDIYRLGQRFDFFRMLSCYFTTIGFYFSSLISVIGIYIYLYGQLYLVLSGLQKTLILEAKVKNIKSLETALASQSFLQLG
LLTGLPMVMEIGLEKGFLIAFQDFILMQLQLAAFFFTFSLGTKTHYFGRTILHGGARYRPTGRKVVVFHANFSEN
YRLYSRSHFIKGFELIILLVVYELFKHTSQSNMAYSFITFSVWFMSLTWLCAPFLFNPSGFTWEIIVGDWRDWNR
WIKEQGGIGIQQDKSWQSWWNDEQAHLRGSGVGARCLEIVLSLRFFLYQYGLVYHLDITQSSTNIIVYALSWVV
ILATFLTVKAVDLGRQLFSTRKHLVYRFFKVFVFVSILTVIITLSNICHLSIKDLIVSCLAFLPTGWGLILIAQAVRPKIE
GTSLWEFTQVLARAYDYGMGVVLFAPMAILAWLPIISAFQTRFLFNEAFNRRLQIQPILAGKKKK*

>BolC05g058550.2J/BoCALS9

MSRAESSWERLVNAALQRDRAGGGSGGGGPGQGSLMEYVPSSLANNRDIDAILRAADELQDEDPSIARILCE
HAYSLAQNLDPNSEGRGVLQFKTGLMSVVKQKLAKREVGTIDRSQDIKRLQDFYRLYREKNNVDTLKEDEKQLR
ESGVFTKEMERKTLRRKRVFATLKVLGNVLEQVAKEIPDELKHVIDSDAAMSEDTIAYNIIPLDAPVTTNATTSFPE
VQAAVAALKYFPGLPKLPADFPIPATRNADMLDFLHYIFGFQKDSVSNQREHIVLLLANEQSRLNIPEEREPKLDD
AAVRKVFLKSLDNYIKWCDYLCIQPAWSNLETISEEKRLLFLSLYFLIWGEAANIRFLPECLCYIFHHMVREMDEIL
RQQVARPAESCMPVDSRGSDDGVSFLDHVIAPLYGVVSAEAFNNDNGRAPHSAWRNYDDFNEYFWSLHSFE
LGWPWRTSSSFFQKPIPRKKYDLQTGRATHRGKTSFVEHRTFLHLYHSFHRLWIFLAMMFQTSLQALAIIAFNK
NDIYSRKTLREILSLGPTFVVMKFSESVLDVIMMYGAYSTTRRLAVSRIFLRFIWFSLASIVISFLYVKALQEDSNPN
SNSVTFKFYVILIAIYGGVQLFLSILMRFPTCHNIANKCDRWPVIRFFKWMRQERHYVGRGMYEKTSDFIKYLLF
WLVVLSAKFSFAYFLQIEPLVGPTRMIVKQDNIQYSWHDLVSRNNYNALTVASLWAPVVAIYLLDIHIFYTIVSAFL
GFLLGARDRLGEIRSLEAIHKQFEEFPGAFMKALHVPITNRLDSFPSAADKNKVDAAHFAPFWNQIIKCLREEDYI
TDFEMDLLLMPKNSGRLQLVQWPLFLLSSKILLAKEIAAESNTQEEMIERIERDDYMKYAVEEVYHTLKLVLMET
LEAEGRMWVERIYEDIQASIKDRKIHHDFQLNKLSLVITRVTALLGILKENETPEHAKGAIKALQDLYDVMRLDILT
FNMRGQYETWNILTQAWNEGRLFTKLKWPKDPELKALVKRLYSLFTIKDSAAHVPRNLEARRRLQFFTNSLFM
DVPPPKSVDKMLSFSVFTPYYSEVVLYSMAELTKRNEDGISILFYLQKIYPDEWKNFLARIGQDENALEGDLRNE
RDILELRFWASYRGQTLARTVRGMMYYRKALMLQSYLERKAGRDGESTPYGNDATDAEGFELSPEARAQADL
KFTYVVTCQIYGRQKEDQKPEAADIALLMQRNEALRIAYIDVVDTPKEGKSHTEYYSKLVKADISGKDKEIYSIRLP
GDPKLGEGKPENQNHAIVFTRGNAVQTIDMNQDNYFEEALKMRNLLEEFDPNRGHGIRPPTILGVREHVFTGS
VSSLASFMSNQETSFVTLGQRVLAKPLKIRMHYGHPDVFDRVFHITRGGISKASRVINISEDIFAGFNSTLRQGNI
THHEYIQVGKGRDVGLNQIALFEGKVAGGNGEQVLSRDVYRLGQLLDFFRMMSFYFTTVGFYFCTMLTVLTVYI
FLYGRLYLALSGVGATIRERAILLDDTALSAALNAQFLFQIGVFTAVPMILGFILEQGFLQAIVSFTTMQFQLCTIFFT
FSLGTRTHYFGRTILHGGARYQATGRGFVVKHIKFSENYRLYSRSHFIKAMEVILLLVVYLAYGNDEAGAVSYILLTV
SSWFLVVSWLFAPYLFNPAGFEWQKVVEDFKEWTNWLFYRGGIGVKGGESWEAWWEEEISHIRTLSGRIVETI
LSLRFFIFQYGIVYKLNLQGSDTSIAVYGWSWAAFAMLIVLFKVFTFSQKMSVNFQLVLRFIQGVSLLMALAGIVV
AIVLTSLSVTDIFASILAFIPTGWGILSIACAWKPVIKRLGMWKSVRSLARLFDAGMGMLIFLPVALCSWFPFVSTF
QTRMMFNQAFSRGLEISLILAGNNPNSGL*

>BolC06g024740.2J/BoCALS10
MTITRTLFESSTAKCYAPSFVAVNLCLALIDGALAFIAFLQLSRFHRRDKRVGWTRQKVLHLMIGSSNTGSLVYFV
AAVIAMHHWSNAFGFLLMAFPKILFLATFLLLLSFWVDVCHQGNGEEEDDDDEESSIQQVFLEKSKSKPGSSSA
SDRRKCCSFHGIHVGTRQKFVVAAVVLVFILMISFAILIWIASGDNSADPSLLAEVYVDIFASILLITGGGLCFYGM
RLLFNLRKVRSEQVSSEMRKVSGLAGVSVVCFSVSSLIALLTHIPLFYHWNPNKLHGINALVLLIIYYFIGSTLPLAF
VLWVLRELPPQNIVSRQEEQTRITYVNYDTVPRQPPQQWTSTTVSKNQERVTDCSYSRLAMEASSSGTAELPR
SLSRRAAPSRATTMMIDRPNEDASAMDSELVPSSLASIAPILRVANEIEKDNPRVAYLCRFHAFEKAHRMDDTSS
GRGVRQFKTYLLHRLEKEEEETKPQLAKSDPREIQAYYQNFYEKYIKEGETSRKPEEMARLYQIASVLYDVLKTVV
PSPKVDYETRRYAEEVERKRDRYEHYNILPLYAVGTKPAIVELPEVKAAFSAVRNVRNLPKRRVHLPSNAPDEMR
KARSRRFNDILEWLASEFGFQRGSVANQREHIILLLANADIRNRTDEEYDGLKSSTVTELMDKTFKNYYSWCKYL
HREPNLKFPPDCDEQQLQLIYISLYLLIWGEASNVRFMPECICYIFHHMANDVYGILFSNVEAVSGEAYETGEIIDE
ETFLRNVITPIYQVIRDEARRNKGGTASHSQWRNYDDLNEYFWSRKCFKIGWPLDPNADFFQNSDESTPQNER
LNQVTYGKRKPKTNFVEVRTFWNLFRDFDRMWIFFVMAFQAMVIVGWNGSGSLGEIFDKDIFKKVLTIFITSA
YLTLLQAALDIILTFNAWKNFKLSQILRYLLKLAVAAMWAVLLPIAYSKSVQRPSGVVKFFSTWTGDWKDKSFYNY
AVSFYVLPNILSAFLFLIPPFRRVMECSDMRIIKLIMWWSQASLKFILGMHEDMLSLFKYTSFWIMLLISKLSFNY
YVEILPLIKPTKMIMNLHIRNYQWHEFFPHANNNVGVVIAVWAPIVLVYLMDAQIWYAIFSTLFGGIHGAFSHL
GEIRTLGMLRSRFESIPIAFSRTLMPTEDANRKHADDYGDQKKITNFSQVWNEFIISMRREDKISDRDRDLLLVPS

SSGDVSVIQWPPFLLASKIPIAVKMAKDFKGKEDAELFRKVTSDNYMHYAVTESYETLKKIISALLEDNADRRVM
NQVFSEVDMSIQKQRFIYDFRMSGLTLLSDKLEKFLSILLSDYEDEGTYKSQLINVFQDVIEIITQDLLVNGHEIVEK
ARIHSPDIKNEKKEQRFEKINIHLIQDKRWREKVVRLHLLLSVKESAINVPQNLEARRRITFFANSLFMNMPNAP
RIRDMLSFSVLTPYYKEDVLYSEEELNKENEDGISILFYLQKIYPDEWTNFLDRLNDPKLLEKDKSEFLREWVSYRG
QTLARTVRGMMYYRQALELQCYQEVAGENGLWFTNLEAKHEILQLTIFLLLLAAKFSLHQAMASNDEHQKAFL
ERAKALADLKFTYVVSCQVYGNQKKSGDIHNRSCYTNILQLMLKYPSLRVAYVDEREETADAKSPKVFYSVLLKG
GAKFDEEIYRIKLPGPPAEIGEGKPENQNHAIIFTRGEALQTIDMNQDNYFEEAFKLRNVLEEFKKERVGRRKPTI
LGLREHIFTGSVSSLAWFMSNQESSFVTIGQRILANPLRVRFHYGHPDIFDRIFHITRGGVSKASKVINLSEDIFGG
FNSTLRGGYVTHHEYIQVGKGRDVGLNPISIFEAKVANGNGEQTLSRDVYRLGHRFDFYRMLSFYFTTIGFYFSS
MLTVITVYAFLYGRMYMVMSGAEKEILRLATPNQLAALEQALATQSIFQLGFLMVLPMVMEIGLEEGFRSAIVD
FFIMQLQLASVFFTFQLGTKSHYYGRTILHGGSKYRPTGRGFVVFHAKFAENYRLYSRSHFVKGLELLLLLIVYQVY
GHSYRSSNLYLYITVSMWFMVGSWLFAPFIFNPSGFEWQKTVDDWTDWKRWLDDRGGIGIPVDKSWESW
WSVEQEYLKHTNIRGRILEITLALRFFIYQYGIVYQLNISQNSKSFLVYGLSWVVLLTSLLVLKMVSMGRRKFGTDF
QLMFRILKALLFLGFLSVMTVLFVVCELTLTDLSASILAFLPTGWAILLIGQVLRSPIKALGIWDSVKELGRAYEKIM
GLVIFAPIAVLSWFPIVSEFQARLLFNQAFSRGLQISMILAGRKDKAASSYK*

>BolC08g058150.2J/BoCALS11
MASTSGGRGGEDGRPPQMQPVRSLSRRMTRAGTMMMIEPNEDESIIDSELVPSSLAAIAPILRVANDIEEDNP
RVAYLCRFHAFEKAHKMDPTSSGRGVRQFKTYLLHKLQEEEPTSDPNEIQTYYQKFYVDNIENGEGKKTPEEMA
KLYQMATVLYDVLKTVIHPARIDEKTHRYAKEVERKKDHYEPYNILPLDVGGAKTVIMELPEIKAAIRAVCNVQNL
PHPRVPSASTKPNEVDREKARTFNDILEWLALVFGFQRGNVANQREHLILLLANVDVRKRNLEDYEDVKPSTVN
KLMGKYFKNYKSWCNYLRMESYLRFPAGCNEQQLSLLYIGLYLLIWGEASNVRFMPECLCYIFHNMANEVHGIL
FSNVYPVTGETYEAGAPDDEAFLRNVITPIYQVLRKEVRRNKMGKASHSKWRNYDDLNEYFWDKRCFRLDWP
MKPEADFFIHTDVISQRPNERHDPVSHGKRKPKTNFVEARTFWNLYRTFDRMWMFLALSLQVMIIVAWSPSG
SILNIFSEDVFKNVLTIFITSAFLNLLQATLDVILSFGAWKSLKFTQILRYITKFLMAAMWAIILPITYSNSLQNPTELI
KFFSSWIGSWLHQSSYNYAIALYVLPNILAAVFFLLPPLRRIMERSNMRIVTFIMWWAQPKLYVGRGMHEEMF
ALFKYTFFWVMLLLSKLAFSYYVEILPLVKPTRLIWDMTGVNYQWHEFFPNATHNIGVIISIWGPIVLVYFMDTQI
WYAIFSTLFGGISGAFSHLGEIRTLGMLRSRFRFVPSAFCGKLTPLPPGHPRRKHLEETVDERDIARFSQMWNKF
VYTMRDEDLISDRERDLLLVPSSSKDVTVLQWPPFLLASKIPIALDMAKDFKGKEDIDLFKKIKSEYYMHYAVVEA
YESVRDVIYGLLEDESDKRIVREICYEIDVSIQQHKFLSKFRMIGMPLLSDKLEKFLKILLSGDEEDETYKSQIINVLQ
DIIEIITQDIMVNGHEILERAHFQSGDIDTDKKEQRFEKINLYKQDASWREKVVRLLLLVTVKESAINIPQNLEARR
RMTFFANSLFMNMPDAPRVRDMLSFSVLTPYYKEDVLYSEEELNKQNEDGISILFYLQKIYPEEWSNYVERVTD
AKRNFSDKEKTDQLREWVSFRGQTLSRTVRGMMYYRMSLELQCYQEYTGEDDTHDGYLSSASNENFMNRAR
ALADLKFTYVVSCQVYGNQKKSSEAKDRSCYNNILQLMLKYPSLRVAYIDEREETVNNKSQKVFYSVLLKGGDKL
DEEIYRIKLPGNPTEIGEGKPENQNHAIIFTRGEALQTIDMNQDNYFEESFKMRNVLQEFDEGRRGKRNPTILGL
REHIFTGSVSSLAWFMSNQETSFVTIGQRVLANPLRVRFHYGHPDIFDRIFHITRGGISKASKIINLSEDIFAGYNS
TLRGGYITHHEYIQAGKGRDVGMNQISIFEAKVANGNGEQTLSRDVYRLGRRFDFYRMLSFYFTTVGFYFSSMI
TVVTVYVFLYGRLYLVLSGLEKEILQSATVHQSKALEQALAAQTVFQLGFLMVLPMVMEIGLEKGFRTALGDFIIM
QLQLASVFFTFQLGTKAHYFGRTVLHGGSKYRATGRGFVVFHAKFAENYRLYSRSHFVKGLELVILLVVYQVYGNS
YRSSSLYLYITFSMWFLVSSWLFAPFIFNPSGFEWQKTVDDWTDWKRWMGNRGGIGIVVEKSWESWWESEQ
EHLKHTNLRGRVLEILLALRFLLYQYGIVYHLNVAHRDTTLLVYGLSWAVLLSVLLVLKMVSMGRRKFGTDFQVM
FRILKALLFLGFLSVMTVLFVVCGLTISDLCASFLAFLPTGWAILLIGQTLRGVLKGIGIWDSIKELGRAYESIMGLLIF
TPIAVLSWFPFVSEFQTRLLFNQAFSRGLQISMILEGKKDKETPSLSNKKRLLRFYTHFRKATQHREKVRKHHIEQ
TKPPPPSLGSRRRLGGPSFMPQLVKAKAFPETHNVISPLQSLALLITDPRKMQHGGNKSGKSSTNVWANNASL

VKTMAALDEFKSGFPSKGLATVSNKWWGTSGQRQDAGLEIITEEDVKDEEAASEKKQSSLLAVRRRIAEEGREA
LELGVSQGFGSKRPDKRDQHLLSQIFGSSLPKEWVNDSS*

>BolC09g002840.2J/BoCALS12
MSLRHRTTVPSQPGRPPAAGAIDEEPYNIIPVNNLLADHPSLRYPEVRAAAAALKTVGDLRRPTYVQWRPHYD
LLDWLALFFGFQKDNVRNQREHLVLHLANAQMRLTPPPDNIDSLDPAVVRRFRRKLLGNYSSWCSYLGRKSNI
WISDRNPDSRRELLYVGLYLLVWGEAANLRFMPECICYIFHNMASELNKILEDCLDESTGQPYSPRITGENSFLN
GVVKPIYETIKAEINESKNGTEPHCKWRNYDDINEYFWTDRCFSKLKWPIDLGSSFFKSSRGSGVGKTGFVERRT
FFYLYRSFDRLWVMLALFLQAAIIVAWEEKPGGGSVRSQLWNALKSTDVQVRLLTVFLTWSGMRLLQAVLDAG
SQRPLISRETKRLFFRMLMKVVAATVWIIAFIVLYTNIWKQRKQDRQWSRAANDKIYQFLYAVVAFLVPEILALAL
FIVPWIRNFLEETNWKIFFALTWWFQGKSFVGRGLREGLVDNIKYSTFWIFVLATKFTFSYFLQVKPMIKPSKLL
WNLKEVDYEWHQFFGKSNRFSVLLLWLPVVLIYLMDIQIWYAIYSSIVGAVVGLFDHLGEIRDMGQLRLRFQFF
ASAIQFNLMPEEQLLNARGFGNKLKDAIHRLKLRYGLGRPFKKLESNQVEANKFALIWNEIILAFREEDIVSDREV
ELLELPKNSWNVTVIRWPCFLLCNELLLALSQAKELVDAPDKWLWHKICKNEYRRCAVVEAYESIKHLLLSIIKIDT
EEHKIVTIFFQMIEVSIQGEQFTKTFKVDLLPKIYETLQKLVGLLNDEKVDVGRVVNGLQSIYEIATRQFFIEKKTTE
QLSTEGLTPHDPASKLLFQNAVRLPDASNEDFFRQVRRLHTILTSRDSMHSVPVNLEARRRIAFFSNSLFMNLPH
APQVEKMLAFSVMTPYYSEEVVYSKEQLRNETEDGISTLYYLQTIYADEWKNFKERMRREGIKTDVELWTTKLR
ELRLWASYRGQTLARTVRGMMYYYRALKMLAFLDSASEMDIREDAQELGSMRSSQGNRLDGVDDVNDRSSL
SRATSSVSMLYKGHEYGTALMKFTYVVACQIYGSQKAKKEPQAEEILYLMKQNEALRIAYVDEVHAGRGETEYYS
VLVKYDHTLEREVEIFRVKLPGPVKLGEGKPENQNHAMIFTRGDAVQTIDMNQDNYFEEALKMRNLLQEFRHY
HGIRKPTILGVREHIFTGSVSSLAWFMSAQETSFVTLGQRVLANPLKVRMHYGHPDVFDRFWFLSRGGISKASR
VINISEDIFAGFNCTLRGGNVTHHEYIQVGKGRDVGLNQISMFEAKVASGNGEQVLSRDVYRLGHRLDFFRMLS
FFYTTVGFFFNTMMVILTVYAFLWGRVYLALSGVEKSALADSTDTNAALAVILNQQFIIQLGLFTALPMIVEWSLE
EGFLLAIWNFIRMQIQLSSVFYTFSMGTRAHYFGRTILHGGAKYRATGRGFVVEHKSFTENYRLYARSHFVKAIEL
GLILIVYATHSPIAKDSLIYIAMTLTSWFLVISWILAPFVFNPSGFDWLKTVYDFEGFMNWIWYQGRISTKSEQSW
EIWWYEEQDHLRTTGLPGRIMEIILDLRFFFFQYGIVYQLKIANGSTSVLVYLLSWIYIFAVFVFFLVIQYARDKYSA
RNHIRYRLVQFLLIVFGTLVIVALLEFTHFSFVDIFTSLLAFVPTGWGILLIAQALRPALQKIGLIWNAVISLARLYDILF
GIVIMVPVAFMSWMPGFQSMQTRILFNEAFSRGLRIMQIVTGKKSKGDVEVEKRR*

>BolC09g011540.2J/BoCALS13
MAQSSTSHDSGPQGLMRRPSRSAATTMSIEVFDHEVVPASLGTIAPILRVAAEIEHERPRVAYLCRFYAFEKAHR
LDPSSSGRGVRQFKTLLFQRLERDNASSLASRVKKTDGREVESYYQQYYEHYVRALDQGDQADRAQLGKAYQT
AGVLFEVLMAVNKSEKVEAVAPEIIAAARDVQEKNEIYAPYNILPLDSAGASQSAMQLEEVKAAVAALGNTRGL
NWPSGFEQHKKKSGNLDLLDWLRAIDNVRNQREHLVCLLADNHIRLTPRPEPLNKLDDRAVDAVKTKLFKNYK
NWCKFLGRKHSLRLPQGAEDIQQRKILYMGLYLLIWGEAANIRFMPECLCYIFHNMAYELHGLLAGNVSIVTGE
NIKPSYGGDDEAFLRKVITPIYRVVEKEASKSANGKAAHSDWSNYDDLNEYFWSPDCFSLGWPMRDDGDFFK
STRDMAQGKKGSLRKAGNTGKSNFTETRTFWHIYHSFDRLWTFYLLALQAMIILAFKRVELREILNKDVLYSLSSI
FITAAFLRFLQSTPPSMLRQNFPCLEFFCFRHLVTLPGSNDYIVSCFYARSLGRYLKLPGFPQSVSFAPGMLKQWL
SFLPRVKGVPPLYILAAALYLLPNVLAAIMFIFPMLRRWIENSDWHIIRLLLWWSQPRIYVGRGMHESQISLIKYTI
FWLLLFCCKFAFSYFLQVKLLVKPTNAIMSIRHVKYKWHEFFPDAEHNYGAVVSLWLPVILVYFMDTQIWYAIFS
TICGGVIGAFDRLGEIRTLGMLRSRFQSLPGAFNTYLVPSDKTRRRGFSLSKRFAEVTAARRTEAAKFSQLWNEIIS
SFREEDLISDREMDLLLVPYTSDPSLKLIQWPPFLLASKIPIALDMAAQFRTKDSDLWKRICADEYMKCAVIECYE
SFKHVLHTLVIGENEKRIIGIIKEVESNISKNSFLSNFRMAPLPALCSKFVELVGILKDADPSKRDTVVLLLQDMLEV
TTRDMMQNENRELVELGHTNKESGRQLFAGTDAKPAILFPPVETAQWDEQIRRLHLLLTVKESAMDVPINLEA

RRRIAFFSNSLFMDMPRAPRVRNMLSFSEETVYSKNDLEMENEDGISVVYYLQKIFPDEWTNFLERLGCKDETA
VLESDENILQLRHWVSLRGQTLFRTVRGMMYYRRALKLQAFLDMATEKEILEGYKAISEPTEEDKKSQRSLYAQL
EAVADLKFTYVATCQNYGNQKRSGDRRATDILNLMVNNPSLRVAYIDEVEEREGGKVHKVFYSVLIKAVENLDQ
EIYRVKLPGPAKIGEGKPENQNHALIFTRGEALQAIDMNQDHYLEEALKMRNLLEEFNEDHGVRAPTILGFREHI
FTGSVSSLAWFMSNQETSFVTIGQRVLASPLKVRFHYGHPDVFDRIFHITRGGISKASRGINLSEDIFAGFNSTLR
RGNITHHEYIQVGKGRDVGLNQISLFEAKVACGNGEQTLSRDLYRLGHRFDFFRMMSCYFTTVGFYISSMIVVLT
VYAFLYGRLYLSLSGVEEAIVKYAAAKGDSSLKAAMASQSVVQLGMLMTLPMIMEIGLERGFRTALCDLIIMQLQ
LAPVFFTFSLGTKVHYYGRTILHGGAKYRATGRGFVVRHEKFAENYRMYSRSHFVKGMELMVLLICYRLYGKATE
DSVAYVLVLGSTWFLVASWLFSPFLFNPSGFEWQKIVDDWDDWNKWISSRGGIGVPAVKSWESWWEEEQEH
LLHSGFFGKFWEIFLSLRYFIYQYGIVYHLNLTKESRLGKQQSVIVYGLSWLVIVAVMIVLKIVSMGRKKFSADFQL
MFRLLKLFLFIGSVVIVGMLFHFLKLTVGDILQSFLAFLPTGWALLQISQVGRTLMKAVGMWGSVKALARGYEYI
MGVVIFMPVTILAWFPFVSEFQTRLLFNQAFSRGLQIQRILAGGKKQK*

>BolC09g029770.2J/BoCALS14
MSMRPRPSPATAHAPSQEVYNIIPIHDFLTEHPSLRYPEVRAAAAALKVVGDLPKPPFVDFTPRMDLMDWLGL
LFGFQLDNLRNQRENLVLHLANSQMRLQPPPLHPDGLDPTVLRRFRKKLLRNYTNWCSFLGVRCHVTSPAHSR
HQTNVVLNLRRELLYVALYLLIWGESANLRFMPECICYIFHHMAMELNKVLGGEFDDMTGMPYWPSFSGDCA
YLKSVVMPIYRTVKTEVESSNNGTKPHSAWRNYDDINEYFWRKRALKSLKWPLDCTSNFFDTTPKSSRVGKTGF
VEQRSFWNVFRSFDRLWILLLLYLQAAIIVATSRVQYPWQDKDVAVALLTIFISWAGLRLLQSVLDASTQYSLVGR
ETFWLFVRLILKLVVAVTWTVLFSVFYARIWSQKNKDGRWSRAADDRIIVFLKVVFVYVIPEMLALVLFIVPCIRN
WIEELNLGIVYFFTWWFYSKSFVGRGLREGLVDNVKYSIFWIVVLATKFIFSYFLQIRPLIKPTRALLNLKNAPYNW
HEFFGSTHRIAVVMLWLPVILVYLMDLQIWYSIYSSLVGATIGLFSHLGEIRNIDQLRLRFQFFSSAMQFNLKPEE
HLLGPKATVLKKVRDAIHRLKLRYGIGQPFNKIESSQVEATWFALLWNEIILTFREEDLISDREVELLELPPNCWNI
QVIRWPCFLLCNELLLALSQANELCDAPDRWLWSKICSSEYRRCAVIEAFDSIKFVIRKIVKNGTEEESIVNRLLNEI
DENVEIGKVTEVYKLTVLLRIHEKLISLLERLMDPDKKVFRIVNILQALYELCAWEFPRIRRSTQQLRQLGLAPVSLN
ADTELLFVNAINLPPPGDVVFYRQIRRVHTILTSRDPMHNVPKNLEARERLAFFSNSLFMNMPQAPSVEKMLA
FSVLTPYYDEEVMYRQEMLRAENEDGISTLFYLQKIYEDEWVNFVERMRREGVENENDIWSQKVRDLRLWAS
YRGQTLSRTVRGMMYYYSALKKLAFLDSASEMDISMGTQIAPEPPRSYYTSDGGDNTLQPTASQEISRMVSGIS
HLYKGSESGSAMMKFTYVVACQVYGQHKAKGDHRAEEILFLMKSHEALRIAYVDEVNLGLGNVEYYSVLVKFD
QRLQREVEIYRIRLPGPLKLGEGKPENQNHAIIFTRGDAIQTIDMNQDNHFEEALKMRNLLESFKKNYGIRKPTIL
GVREKVFTGSVSSLAWFMSSQETSFVTLGQRVLANPLKVRMHYGHPDVFDRFWFIPRGGISKASRVINISEDIF
AGFNCTLRGGNVTHHEYIQVGKGRDVGLNQISMFEAKVASGNGEQALSRDVYRLGHRLDFFRMLSFFYTTVG
YYFNTMLIVFTVYAFLWGRLYLALSGVEKIAKDRSSSNEALGAILNQQFVIQLGLFTALPMILENSLERGFLPAIWD
FITMQLQLASFFYTFSLGTRSHYFGRTILHGGAKYRATGRGFVVEHKKFAENYRLYARTHFIKAIELAIILLVYAAYSP
LAKSSLVYILMTISSWFLITSWIISPFLFNPSGFDWLKTVYDFDDFMSWLWSRGGLFTKADQSWFTWWNEEQD
HLKTTGVWGKLLEILLDLRFFFFQYSIVYHLRIADGQTSIGVYLVSWGCIIGIAAIYITTIYAQKRFSVKEHIKYRFIQF
LVIWLTVLVVVLMLQFTKLTVVDLLISLLAFIPTGWGLISIAQVLRPFLISTVVWDTVISVARLYDLCFGLIVMAPVA
LLSWLPGFQNMQTRILFNEAFSRGLQISIILAGKKSA*

>BolC09g060050.2J/BoCALS15
MSASRGGGPDQGPSQPQQRRIMRTQTVGNLGESFDSEVVPSSLVEIAPILRVANEVESSNPRVAYLCRFYAFEK
AHRLDPTSSGRGVRQFKTALLQRLEREHDPTLMGRVKKSDAREMQSFYQHYYKKYIQALQNAADKADRAQLT
KAYQTANVLFEVLKAVNLTQSIEVDREILEAQDKVAEKTQLYVHYNILPLDPDSANQAIMRYPEIQAAVLGLRNTR
GLPWPEGHKKKKDEDMLDWLQEMFGFQKDNVANQREHLILLLANVHIRQFPKPDQQPKLDDQALTDVMKK

LFKNYKKWCKYLGRKSSLWLPTIQQEMQQRKLLYMALYLLIWGEAANLRFMPECLCYIYHHMAFELYGMLAGN
VSPMTGENVKPAYGGEEDAFLRKVVTPIYEVIAMEAQRSKKGKSKHSQWRNYDDLNEYFWSVDCFRLGWPM
RADADFFYPPVEETNIEKDGDNSKPAVARDRWVGKVNFVEIRSFWHVFRSFDRMWSFYILCLQAMIIMAWD
GGEPSSVFDAGVFKKVLSVFITAAIMKLGQATLDVILNFKAHRSMSLHVKLRYILKVISAAAWVIILPVTYAYSWK
DPPAFARTIKSWFGSAMHSPSLFIIAVVFYLAPNMLAAVLFMFPMLRRFLERANFRIVMLMMWWSQPRLYVG
RGMHEGAFALLKYTMFWVSLIATKLAFSYYIEIKPLVAPTQAIMRARVTNFQWHEFFPRAKNNIGVVIALWAPII
LVYFMDSQIWYAIYSTIFGGIYGAFRRLGEIRTLGMLRSRFESLPGAFNDRLIPDGNQQRKKKGLRATLSHNFTED
KVPVNKEKEAARFAQLWNTIISSFREEDLISDREMDLLLVPYWADRDLDLIQWPPFLLASKIPIALDMAKDSNGK
DRELMKRIESDSYMKCAVRECYASFKNIINFLVQGNREKEVIEIIFSEVDKHIDTGALIQEYRMSALPSLYDHFVKLI
KYLLDNNVEDRDHVVILFQDMLEVVTRDIMMEDYNISSLVDSSQGGAWHGGMVPLEQQYQLFASSGAIRFPI
EPVTEAWKEKIKRLHLLLTTKESAMDVPSNLEARRRISFFSNSLFMDMPAAPKVRNMLSFSVLTPYYTEEVLFSM
HDLDTPNEDGVSILFYLQKIFPDEWNNFLERVKCYSEEEIKESVDLEEELRLWASYRGQTLTRTGILFWQFTSQW
KIRWINKIFVNIFAVRGMMYYRKALELQAFLDMAMHEDLMEGYKAVELNSEDTSRGERSLWAQCQAVADMK
FTYVVSCQQYGIHKRSGDQRAQDILRLMTRCAELSYFVFFFLKKTCLKLMAFLITIGRYPSLRVAYIDEVEETVKDT
SKKGNQKVYYSVLVKVPKSTDHSSLAQNLDQVIYRIKLPGPAILGEGKPENQNHAIIFSRGEGLQTIDMNQDNY
MEEALKMRNLLQEFLTKHDGVRHPSILGLREHIFTGSVSSLAWFMSNQETSFVTIGQRLLANPLRVRFHYGHPD
VFDRLFHLTRGGVSKASKVINLSEDIFAAGFNSTLREGNVTHHEYIQVGKGRDVGLNQISMFEAKIANGNGEQT
LSRDIYRLGHRFDFFRMMSCYFTTVGFYFSTLITVLTVYIFLYGRLYLVLSGLEQGLSTQKGIRDNTPLQIALASQSF
VQIGFLMALPMLMEIGLERGFRTALSEFVLMQLQLAPVFFTFSLGTKTHYYGRTLLHGGAKYRSTGRGFVVFHA
KFADNYRLYSRSHFVKGLEMLLLLVVYQIFGSAYRGVLAYLLITISMWFMVGTWLFAPFLFNPSGFEWQKIVDD
WTDWNKWINNIGGIGVPAEKSWESWWEEEQEHLRHSGKRGIVVEILLSLRFFIYQYGLVYHLTITERTKNFLVY
GVSWLVIFLILFVMKTISVGRRKFSASFQLMFRLIKGLIFMTFIAIIVILITLAHMTIQDIIVCILAFMPTGWGMLLIA
QACKPVVHRAGFWGSVRTLARGYEIVMGLLLFTPVAFLAWFPFVSEFQTRMLFNQAFSRGLQISRILGGHRKD
RSSRNKE*

3. BrCALS

>Bra032416
MASTSGGRGGEDGRPPQMQPVRSLSRRMTRAGTMMMIEPNEDESIIDSELVPSSLAAIAPILRVANDIEEDNP
RVAYLCRFHAFEKAHKMDPTSSGRGVRQFKTYLLHKLQEEEPTSDPNEIQTYYQNFYVDNIENGEGKKTPEEMA
KLYQMATVLYDVLKTVIHPARIDEKTHRYAKEVERKKDHYEPYNILPLDVGGAKTVIMELPEIKAAIRAVCNVQNL
PQPRVPSASTKPNEVDREKARTFNDILEWLALVFGFQRGNVANQREHLILLLANVDVRKRNLENYEDVKPSTV
NKLMEKYFKNYKSWCNYLRMESYLRFPAGCDEQQLSLLYIGLYLLIWGEASNVRFMPECLCYIFHNMANEVHGI
LFSNVYPVTGETYEAGAPDDEAFLRNVITPIYQVLRKEVRRNKMGKASHSKWRNYDDLNEYFWDKRCFRLDW
PMKPEADFFIHTDVISQRPNERHDPVSHGKRKPKTNFVEARTFWNLYRTFDRMWMFLALSLQVMIIVAWSPS
GSILNIFSEDVFKNVLTIFITSAFLNLLQATLDVILSFGAWKSLKFTQILRYITKFLMAAMWAIILPITYSNSLQNPTGL
IKFFSSWIGSWLHQSSYNYAIALYVLPNILAAVFFLLPPLRRIMERSNMRIVTFIMWWAQPKLYVGRGMHEEMF
ALFKYTFFWVMLLLSKLAFSYYVEILPLVKPTRLIWDMTGVNYQWHEFFPNATHNIGVIISIWGPIVLVYFMDTQI
WYAIFSTLFGGISGAFSHLGEIRTLGMLRSRFRFVPSAFCGKLTPLPPGHPKRKHLEETVDERDIARFSQMWNKF
VYTMRDEDLISDRERDLLLVPSSSKDVTVLQWPPFLLASKIPIALDMAKDFKGKEDIDLFKKIKSEYYMHYAVVEA
YESVRDVIYGLLEDESDKRIVREICYEIDVSIQQHKFLSKFRMTGMPLLSDKLEKFLKILLSGDEEDDTYKSQIINVL
QDIIEIITQDIMVNGHEILERAHFQSGDIDTDKKEQRFEKINLYKQDASWREKVVRLLLLVTVKESAINIPQNLEAR
RRMTFFANSLFMNMPDAPRVRDMLSFSVLTPYYKEDVLYSEEELNKENEDGISILFYLQRIYPEEWSNYVERVID

VKRNFSDKEKTDQLREWVSFRGQTLSRTVRGMMYYRMSLELQCYQEYTGEDDTNDGYLSSASNENFMNRAR
ALADLKFTYVVSCQVYGNQKKSSEGRDRSCYNNILQLMLKYPSLRVAYIDEREETVNNKSQKVFYSVLLKGGNKL
DEEIYRIKLPGNPTEIGEGKPENQNHAIIFTRGEALQTIDMNQDNYFEESFKMRNVLQEFDEGRRGKRNPTILGL
REHIFTGSVSSLAWFMSNQETSFVTIGQRVLANPLRVRFHYGHPDIFDRIFHITRGGISKASKIINLSEDIFAGYNS
TLRGGYITHHEYIQAGKGRDVGMNQISIFEAKVANGNGEQTLSRDVYRLGRRFDFYRMLSFYFTTVGFYFSSMI
TVVTVYVFLYGRLYLVLSGLEKEILQSATIHQSKALEEALAAQTVFQLGFLMVLPMVMEIGLEKGFRTALGDFIIM
QLQLASVFFTFQLGTKAHYFGRTVLHGGSKYRATGRGFVVFHAKFAENYRLYSRSHFVKGLELVILLVVYQVYGNS
YRSSSLYLYITFSMWFLVSSWLFAPFIFNPSGFEWQKTVDDWTDWKRWMGNRGGIGIVVEKSWESWRESEQE
HLKHTNLGGRVLEILLALRFLLYQYGIVYHLNVAHRDTTLLVYGLSWAVLLSVLLVLKMVSMGRRKFGTDFQVMF
RILKALLFLGFLSVMTVLFVVCGLTISDLCASFLAFLPTGWAILLIGQTLRGVLKGIGIWDSIKELGRAYEYIMGLLIF
TPIAVLSWFPFVSEFQTRLLFNQAFSRGNSASRESQKAPYRPDETFLLLVVTDPRKMQHGGNKSGKSSTNVWA
NNANLAKTMGALDEFKSGFPSKGLATVSNKWWGTGGQRQDVTEDGGDVKDEEAASEKQSSLLGIRKRIAEEG
REALELGVSQGFGSKRPDKRDQHLLSQIFGSSLPKEWVNDSS

>Bra005624
MSQRRGGGGGPDPPPPQRRILRTQTAGNLGEAMLDSEVVPSSLVEIAPILRVANEVEASNPRVAYLCRFYAFEK
AHRLDPTSSGRGVRQFKTALLQRLERENETTLAGRQKSDAREMQSFYQHYYKKYIQALLNAADKADRAQLTKA
YQTAAVLFEVLKAVNQTEDVEVADEILEAHTKVEEKSQIYVPYNILPLDPDSQNQAIMRFPEIQATVIALRNTRGL
PWPAGHKKKLDEDMLDWLQTMFGFQKDNVANQREHLILLLANVHIRQFPRPEQQPKLDDRALTIVMKKLFK
NYKKWCKYLGRKSSLWLPTIQQEVQQRKLLYMGLYLLIWGEAANLRFMPECLCYIYHHMAFELYGMLAGSVSP
TTGEHIKPAYGGDDEAFLQKVVTPIYKTIAKEAKRSRGGKSKHSEWRNYDDLNEYFWSVRCFRLGWPMRADA
DFFCQTAEELRLDKGEDKPKTGDRWMGKVNFVEIRSFWHIFRSFDRMWSFYILSLQAMIIIAWNGSGDLGAIF
HGDVFLKVLSIFITAAILKLAQAVLDIALSWKSRHSMSFHAKLRFILKAIAAAVWVVLMPVTYAYSWRSPSGIAETI
KNWLGGHSGSSPSLFIMVILIYLSPNMLSTLLFVFPFIRRYLERSDIKIVMLMMWWSQPRLYIGRGMHESAWSL
FKYTMYWVVLLISKLAFSFYAEIKPLVVPTKDIMRVHISVYRWHEFFPHAKNNLGVVVALWSPVILVYFMDTQI
WYAIVSTLVGGLNGAFRRLGEIRTLGMLRSRFQSLPGAFNACLVPNEKSETAKKRGIRATFSRKFDQIPSSKDKEA
ARFAQMWNKIISSFREEDLISNREMELLLVPYWADPDLDLIRWPPFLLASKIPIALDMAKDSNGKDRELKKRLSI
DSYMTCAVRECYASFRNLINFLVVGERERQVINEIFAKIDEYIANETLIETLDLSALPDLYGQFVRLIEYLMENKEED
KDQIVIVLLNMLEVVTRDIMDYEVPSLLETAHNGTYVKYDVMTPLHQQKKYFSQLRFPVYSQTEAWKEKAKSA
MDVPSNLEARRRLTFFSNSLFMEMPDAPKIRNMLSFSVLTPYYQEDVLFSIFGLEKQNEDGVSILFYLQKIFPDE
WTNFLERIKCGSEEEIRAREDLEEELRLWASYRGQTLTKTVRGMMYYRKALELQAFLDMAKDEELMKGYKALEL
TSEDASKSGKSLWAQCQALADMKFTFVVSCQQYSIQKRSGDQRAKDILRLMTTYPSLRVAYIDEVEQTHKDSYK
GADDKIYYSALVKAAPQTKPMDSSESVQTLDQVIYRIKLPGPAILGEGKPENQNHAIIFTRGEGLQTIDMNQDNY
MEEAFKMRILLQEFLEKHGGVRTPTILGLREHIFTGSVSSLAWFMSNQENSFVTIGQRVLASPLKVRFHYGHPD
VFDRLFHLTRGGVCKASKVINLSEDIFAGTCFNSLLLVTRFNSTLREGNVTHHEYIQVGKGRDVGLNQISMFEAKI
ANGNGEQTLSRDLYRLGHRFDFFRMLSCYFTTIGFYFSTMLTVLTVYVFLYGRLYLVLSGLEEGLSNQKAFRSNMP
LQAALASQSFVQIGFLMALPMMMEIGLERGFHNALIDFVLMQLQLASVFFTFQLGTKTHYYGRTLFHGGAEYR
GTGRGFVVFHAKFAENYRFYSRSHFVKGIELMILLLVYQIFGHAYRGVVTYILITVSIWFMVVTWLFAPFLFNPSG
FEWQKIVDDWTDWNKWIYNRGGIGVPPEKSWESWWEKEIGHLRHSGKRGIILEIVLALRFFIFQYGLVYQLSTF
KQENQSLWIYGASWFVILFILLIVKGLGMGRRRFSTNFQLLFRIIKGLVFLTFLAILITFIALRLLTPKDILLCMLAFMP
TGWGMLLIAQACKPLIQRLGIWSSVRTLARGYEILMGLLLFTPVAFLAWFPFVSEFQTRMLFNQAFSRGLQISRI
LGGQRKDRSSKNKE

>Bra037213

MAQSSTSHDSGPQGLMRRPSRSAATTMSIEVFDHEVVPASLGTIAPILRVAAEIEHERPRVAYLCRFYAFEKAHR
LDPSSSGRGVRQFKTLLFQRLERDNASSLASRVKKTDGREVESYYQQYYEHYVRALDQGDQADRAQLGKAYQT
AGVLFEVLMAVNKSEKVEAVAPEIIAAARDVQEKNEIYAPYNILPLDSAGASQSVMQLEEVKAAVAALGNTRGL
NWPSGFEQHKKKSGNLDLLDWNQREHLVCLLADNHIRLTPRPEPLNKLDDRAVDAVKTKLFKNYKNWCKFLG
RKHSLRLPQGAEDIQQRKILYMGLYLLIWGEAANIRFMPECLCYIFHNMAYELHGLLAGNVSIVTGENIKPSYGG
DDEAFLRKVITPIYRVVEKEASKSANGKAAHSDWSNYDDLNEYFWSPDCFSLGWPMRDDGDFFKSTRDMAQ
GKKGSLRKAGNTGKSNFTETRTFWHIYHSFDRLWTFYLLALQAMIILAFKRVELREILNKDVLYSLSSIFITAAFLRL
LQSLLDVILNFPGFHRWKFTEILRNILKIVVSLAWCVVLPLCYAQSVSFAPGMLKQWLSFLPRVKGVPPLYILAVAL
YLLPNVLAAIMFSFPMLRRWIENSDWHIIRLLLWWSQPRIYVGRGMHESQISLIKYTIFWLLLFCCKFAFSYFLQV
KLLVKPTNAIMSIRHVKYKWHEFFPDAEHNYGAVVSLWLPVILVYFMDTQIWYAIFSTICGGVIGAFDRLGEIRTL
GMLRSRFQSLPGAFNTYLVPSDKTRRRGFSLSKRFAEVTAARRTEAAKFSQLWNEIISSFREEDLISDREMDLLLV
PYTSDPSLKLIQWPPFLLASKIPIALDMAAQFRTKDSDLWKRICADEYMKCAVIECYESFKHVLHTLVIGENEKRII
GIIIKEVESNISKNSFLSNFRMAPLPALCSKFVELVGILKDADPSKRDTVVLLLQDMLEVTTRDMMQNENRELVEL
GHTNKESGRQLFAGTDAKPAILFPPVATAQWDEQIRRLHLLLTVKESAMDVPINLEARRRIAFFSNSLFMDMPR
APRVRNMLSFSVLTPYYSEETVYSKNDLEMENEDGISVVYYLQKIFPDEWTNFLERLGCKDETAVLESDENILQLR
HWVSLRGQTLFRTVRGMMYYRRALKLQAFLDMATEKEILEGYKAISEPTEEDKKSQRSLYAQLEAVADLKFTYVA
TCQNYGNQKRSGDRRATDILNLMVNNPSLRVAYIDEVEEREGGKVHKVFYSVLIKAVENLDQEIYRVKLPGPAKI
GEGKPENQNHALIFTRGEALQAIDMNQDHYLEEALKMRNLLEEFNEDHGVRAPTILGFREHIFTGSVSSLAWF
MSNQETSFVTIGQRVLASPLKVRFHYGHPDVFDRIFHITRGGISKASRGINLSEDIFAGFNSTLRRGNITHHEYIQ
VGKGRDVGLNQISLFEAKVACGNGEQTLSRDLYRLGHRFDFFRMMSCYFTTVGFYISSMIVVLTVYAFLYGRLYLS
LSGVEEAIVKYAAAKGDSSLKAAMASQSVVQLGMLMTLPMIMEIGLERGFRTALCDLIIMQLQLAPVFFTFSLG
TKVHYYGRTILHGGAKYRATGRGFVVRHEKFAENYRMYSRSHFVKGMELMVLLICYRLYGKATEDSVAYMLVLG
STWFLVASWLFSPFLFNPSGFEWQKIVDDWDDWNKWISSRGGIGVPAVKSWESWWEEEQEHLLHSGFFGKF
WEIFLSLRYFIYQYGIVYHLNLTKESRLGKQQSLIVYGLSWLVIVAVMIVLKIVSMGRKKFSADFQLMFRLLKLFLFI
GSVVIVGMLFHFLKLTVGDILQSFLAFLPTGWALLQISQVGRTLMKAVGMWGSVKALARGYEYIMGVVIFMPV
TILAWFPFVSEFQTRLLFNQAFSRGLQIQRILAGGKKQK

>Bra034218
MSLRHRTVPSQPGRPPAAGAIEDEPYNIIPVNNLLADHPSLRYPEVRAAAAALKTVGDLRRPTYVQWRPHYDLL
DWLALFFGFQKDNVRNQREHLVLHLANAQMRLSPPPDNIDSLDPAVVRRFRRKLLGNYSSWCSYLGRKSNIWI
SDRTPDSRRELLYVGLYLLVWGEAANLRFMPECICYIFHNMASELNKILEDCLDESTGQPYSPKITGENSFLNGVV
KPIYDTIRAEINESKNGTEPHCKWRNYDDINEYFWTDRCFSKLKWPIDLGSSFFKNSRGSGVGKTGFVERRTFFY
LYRSFDRLWVMLALFLQAAIIVAWEEKPGGGSVTSQLWNALKSTDVQVRLLTVFLTWSGMRLLQAVLDAGSQR
SLISRETKRLFFRMLMKVVAATVWIIAFIVLYTNIWKQRKQDRQWSRAANDKIYQFLYAVVAFLVPEILALALFIVP
WIRNFLEETNWKIFFALTWWFQGKSFVGRGLREGLVDNIKYSTFWIFVLATKFTFSYFLQVKPMIKPSKLLWNLK
EVDYEWHQFFGESNRFSVLLLWLPVVLIYLMDIQIWYAIYSSIVGAVVGLFDHLGEIRDMGQLRLRFQFFASAIQ
FNLMPEEQLLNARGFGNKLKDAIHRLKLRYGLGRPFKKLESNQVEANKFALIWNEIILAFREEDIVSDREVELLEL
PKNSWNVTVIRWPCFLLCNELLLALSQAKELVDAPDKWLWHKICKNEYRRCAVVEAYESIKHLLLSIIKIDTEEHKI
VTIFFQMIEVSIQGEQFTKTFKVDLLPKIYETLQKLVGLLNDEKVDVGRVVNGLQSIYEIATRQFFIEKKTTEQLSTE
GLTPHDPASKLLFQNAVRLPDASNEDFFRQVRRLHTILTSRDSMHSVPVNLEARRRIAFFSNSLFMNLPHAPQV
EKMLAFSVMTPYYSEEVVYSKEQLRNETEDGISTLYYLQTIYADEWKNFKERMRREGIKTDVELWTTKLRELRLW
ASYRGQTLARTVRGMMYYYRALKMLAFLDSASEMDIREDAQELGSMRSSQGNRLDGVDDVNDGSSLSRATS
SVSMLYKGHEHGTALMKFTYVVACQIYGSQKAKKEPQAEEILYLMKQNEALRIAYVDEVHAGRGETEYYSVLVK
YDHTLEREVEIFRVKLPGPVKLGEGKPENQNHAMIFTRGDAVQTIDMNQDNYFEEALKMRNLLQEFRHYHGIR

KPTILGVREHIFTGSVSSLAWFMSAQETSFVTLGQRVLANPLKVRMHYGHPDVFDRFWFLSRGGISKASRVINIS
EDIFAGFNCTLRGGNVTHHEYIQVGKGRDVGLNQISMFEAKVASGNGEQVLSRDVYRLGHRLDFFRMLSFFYT
TVGFFFNTMMVILTVYAFLWGRVYLALSGVEKSALADSTDTNAALAVILNQQFIIQLGLFTALPMIVEWSLEEGF
LLAIWNFIRMQIQLSSVFYTFSMGTRAHYFGRTILHGGAKYRATGRGFVVEHKSFTENYRLYARSHFVKAIELGLI
LIVYATHSPIAKDSLIYIAMTLTSWFLVISWILAPFVFNPSGFDWLKTVYDFEGFMNWIWYQGRISTKSEQSWEI
WWYEEQDHLRTTGIPGRIVEIILDLRFFFFQYGIVYQLKIANGSTSILVYLLSWIYIFAVFVFFLVIQYARDKYSARNH
IRYRLVQFLLIVFGTLVIVALLEFTHFSFVDIFTSLLAFVPTGWGILLIAQALRPALQKIGLIWNAVVSLARLYDILFGIV
IMVPVAFMSWMPGFQSMQTRILFNEAFSRGLRIMQIVTGKKSKGDVEVEKRR

>Bra029628
MSRAESSWERLVNAALQRDRAGGASAGGGGPGQGSLMEYVPSSLANNRDIDAILRAADELQDEDPSIARILCE
HAYSLAQNLDPNSEGRGVLQFKTGLMSVVKQKLAKREVGTIDRSQDIKRLQDFYRLYREKNNVDTLKEDEKQLR
ESGVFTKEMERKTLRRKRVFATLKVLGNVLEQVAKEIPDELKHVIDSDAAMSEDTIAYNIIPLDAPVTTNATTSFPE
VQAAVAALKYFPGLPKLPADFPIPATRNADMLDFLHYIFGFQKDSVSNQREHIVLLLANEQSRLTIPEEREPKLDD
AAVRKVFLKSLDNYIKWCDYLCIQPAWSNLETISGEKRLLFLSLYFLIWGEAANIRFLPECLCYIFHHMVREMDEIL
RQRVARPAESCMPVESRGSDDGVSFLDHVIAPLYGVVSAEAFNNDNGRAPHSAWRNYDDFNEYFWSLHSFEL
GWPWRTSSSFFQKPIPRKKYDLQTGRAKHRGKTSFVEHRTFLHLYHSFHRLWIFLAMMFQTSLQALAIIAFNKN
DLYSRKTLREILSLGPTFVVMKFSESVLDVIMMYGAYSTTRRLAVSRIFLRFIWFSLASVVVSFLYVKALQEDSNPN
SNSVMFKFYVILIAIYGGVQFFLSILMRFPTCHNIANKCDRWPVIRFFKWMRQERHYVGRGMYEKTSDFIKYLLF
WVVVLSAKFSFAYFLQIKPLVGPTRMIVKQDNIQYSWHDLVSRNNYNALTVASLWAPVVAIYLLDIHIFYTLVSAF
LGFLLGARDRLGEIRSLEAIHKQFEEFPGAFMRALHVPITNRTSDPSHQAADKNKVDAAHFAPFWNQIIKCLREE
DYITDFEMDLLLMPKNSGRLQLVQWPLFLLSSKILLAKEIAAESNTQEEIIERIERDDYMKYAVEEVYHTLKLVLME
TLEAEGRMWVERIYEDIQASIKDRKIHHDFQLNKLSRVITRVTALLGILKENETPEHAKGAIKALQDLYDVMRLDIL
TFNMRGQYETWNILTQAWNEGRLFTKLKWPKDPELKALVKRLYSLFTIKDSAAHVPRNLEARRRLQFFTNSLFM
DVPPPKSVDKMLSFSVFTPYYSEVVLYSMAELTKRNEDGISILFYLQKIYPDEWKNFLARIGQDENALEGDLRNE
RDILELRFWASYRGQTLARTVRGMMYYRKALMLQSYLERKAGRDGESTPFGNDATDSEGFELSPEARAQADLK
FTYVVTCQIYGRQKEDQKPEAADIALLMQRNEALRIAYIDVVDTPKEGKSHTEYYSKLVKADISGKDKEIYSIRLPG
DPKLGEGKPENQNHAIVFTRGNAVQTIDMNQDNYFEEALKMRNLLEEFDPNRGHGIRPPTILGVREHVFTGSV
SSLASFMSNQETSFVTLGQRVLAKPLKIRMHYGHPDVFDRVFHITRGGISKASRVINISEDIFAGFNSTLRQGNIT
HHEYIQVGKGRDVGLNQIALFEGKVAGGNGEQVLSRDVYRLGQLLDFFRMMSFYFTTVGFYFCTMLTVLTVYIF
LYGRLYLALSGVGATIRERAILLDDTALSAALNAQFLFQIGVFTAVPMILGFILEQGFLQAIVSFTTMQFQLCTIFFTF
SLGTRTHYFGRTILHGGARYQATGRGFVVKHIKFSENYRLYSRSHFIKAMEVILLLVVYLSYGNDEAGAVSYILLTVS
SWFLAVSWLFAPYLFNPAGFEWQKVVEDFKEWTNWLFYRGGIGVKGAESWEAWWEEEISHIRTLSGRIVETIL
SLRFFIFQYGIVYKLNLQGSDTSIAVYGWSWAAFAMLIVLFKVFTFSQKVSVNFQLVLRFVQGVSLLVALAGIVVAI
VLTNLSVTDIFASILAFIPTGWGILSIACAWKPVIKRLGMWKSVRSLARLFDAGMGMLIFLPVALCSWFPFVSTFQ
TRMMFNQAFSRGLEISLILAGNNPNSGL

>Bra008864
MSASRGGGPDQGPSQPQQRRIMRTQTVGNLGESFDSEVVPSSLVEIAPILRVANEVVGSMRSRKLTGWIPPPV
EEEHDPTLMGRVKKSDAREMQSFYQHYYKKYIQALQNAADKADRAQLTKAYQTANVLFEVLKAVNLTQSIEVD
REILEAQDKVAEKTQLYVHYNILPLDPDSANQAIMRYPEIQAAVLGLRNTRGLPWPEGHKKKKDEDMLDWLQE
MFGFQKDNVANQREHLILLLANVHIRQFPKPDQQPKLDDQALTDVMKKLFKNYKKWCKYLGRKSSLWLPTIQ
QEMQQRKLLYMALYLLIWGEAANLRFMPECLCYIYHHMAFELYGMLAGNVSPMTGENVKPAYGGEEDAFLRK
VVTPIYEVIAMEAQRSKKGKSKHSQWRNYDDLNEYFWSVDCFRLGWPMRADADFFYPPVEETNIEKDGDNS

KPAVARDRWVGKVNFVEIRSFWHVFRSFDRMWSFYILCLQAMIIMAWDGGEPSSVFDAGVFKKVLSVFITAAI
MKLGQATLDVILNFKAHRSMSLHVKLRYILKVISAAAWVIILPVTYAYSWKDPPAFARTIKSWFGSAMHSPSLFII
AVVFYLAPNMLAAVLFMFPMLRRFLERANFRIVMLMMWWSQPRLYVGRGMHEGAFALLKYTMFWVSLIAT
KLAFSYYIEIKPLVAPTQAIMRARVTNFQWHEFFPRAKNNIGVVIALWAPIILVYFMDSQIWYAIYSTIFGGIYGAF
RRLGEIRTLGMLRSRFESLPGAFNDRLIPDGNQQRKKGLRATLSHNFTEDKVPVNKEKEAARFAQLWNTIISSFR
EEDLISDREMDLLLVPYWADRDLDLIQWPPFLLASKIPIALDMAKDSNGKDRELMKRIESDSYMKCAVRECYAS
FKNIINFLVQGNREKEVIEIIFSEVDKHIDTGALIQEYRMSALPSLYDHFVKLIKYLLDNNVEDRDHVVILFQDMLE
VVTRDIMMEDYNISSLVDSSQGGAWHGGMVPLEQQYQLFASSGAIRFPIEPVTEAWKEKIKRLHLLLTTKESA
MDVPSNLEARRRISFFSNSLFMDMPAAPKVRNMLSFSVLTPYYTEEVLFSMHDLDTPNEDGVSILFYLQKIFPDE
WNNFLERVKCYSEEEIKESVDLEEELRLWASYRGQTLTRTVRGMMYYRKALELQAFLDMAMHEDLMEGYKAV
ELNSEDTSRGERSLWAQCQAVADMKFTYVVSCQQYGIHKRSGDQRAQDILRLMTRYPSLRVAYIDEVEETVKD
TSKKGNQKVYYSVLVKVPKSTDHSSLAQNLDQVIYRIKLPGPAILGEGKPENQNHAIIFSRGEGLQTIDMNQDNY
MEEALKMRNLLQEFLTKHDGVRHPSILGLREHIFTGSVSSLAWFMSNQETSFVTIGQRLLANPLRVRFHYGHPD
VFDRLFHLTRGGVSKASKVINLSEDIFAGFNSTLREGNVTHHEYIQVGKGRDVGLNQISMFEAKIANGNGEQTLS
RDIYRLGHRFDFFRMMSCYFTTVGFYFSTLITVLTVYIFLYGRLYLVLSGLEQGLSTQKGIRDNTPLQIALASLSFVQI
GFLMALPMLMEIGLERGFRTALSEFVLMQLQLAPVFFTFSLGTKTHYYGRTLLHGGAKYRSTGRGFVVFHAKFA
DNYRLYSRSHFVKGLEMMLLLVVYQIFGSAYRGVLAYLLITISMWFMVGTWLFAPFLFNPSGFEWQKIVDDWT
DWNKWINNIGGIGVPAEKSWESWWEEEQEHLRHSGKRGIVVEILLSLRFFIYQYGLVYHLTITERTKNFLVYGVS
WLVIFLILFVMKTISVGRRKFSASFQLMFRLIKGLIFMTFIAIIVILITLAHMTIQDIIVCILAFMPTGWGMLLIAQAC
KPVVHRAGFWGSVRTLARGYEIGMGLLLFTPVAFLAWFPFVSEFQTRMLFNQAFSRGLQISRILGGHRKDRSSR
NKE

>Bra001239
MSRAESSWKRLVNAALQRDRAGGGAGGGTGQGSLMDYVPSSLPNNRDIDAILRAADELQNEDPSIARILCEH
AYSLAQNLDPKSEGRGVLQFKTGLMSVVKQKLAKREVGTIDRTQDSKRLQDFYRLYREKNNVDTLKKEEMQLR
ESGVLTGELERKTVKRKRLFATLKILGNVLEKVAKEIPDELKHVIDSDAAISEDTIAYNIIPLEAHVTTNAITAFPEVKA
AVAALKYFRGLPNLPANFPIPATRNADMLDFLHYIFGFQKDSVSNQREHIVLLLANEQSRLNIPEEMEPKLDDAA
VHKVFLKSLDNYIKWCDYLGIKPAWSNLERISGEKKLLFVSLYFLIWGEAANIRFLPECLCYIFHHMIVEMNEILRQ
QVSRPAVSCMPVDSHGGSDVGVSFLDHVIAPLYEVVSAEAFHNENGRAPHSEWRNYDDFNEYFWSLRSFELG
WPWRTSSSFFQKPIRRRKYEFEAGRDKHRGKTSFVEHRTFLRLYHSFHRLWIFLAIMFQTSLQALAIIAFNKNSIIS
TKTLRQILSLGPTYVVMKFSESLLDVIMMYGAFSTTRRLAVLRIFLRLIWFSLACVFICFLYVKMLQEDTKPIYNSV
MFKVYGLVIAIYGGIQFLFTILMHIQICRNIASKCDRWAVIRFVEGMRQERHYVGRDMYERMSDFIKYFLFWLV
VLSAKFSFAYFLQIAPLVDPTRMIMKQDNIQYSWHEFVSIKNYNALTVASLWAPVVAIYLLDIHIFYTIASAFFGFLL
GARDRLGEIRSLEAIHKQFEEFPGAFMKALHVPVTNRTFDPSHQAVDKNIVDATHFAPFWNQIIKCLREEDYITD
LEMELLLMPKKSGRLQLVQWPLFLLSSKILLAKEIAAESNSQEEIVERITRDGYMKYAVEEVYHALKLVLTETLEAE
GRMWVERIYEDIDASIKNRKIHNDFQLNKLSLVITRVAALLGILKENETPEHAKGAIKALQDLYDVIRLDVLNVNM
RGQYEMWNNLTQAWNEGRLFTELKWPKDPELKALVRRLYSLFTVKDSAAHVPRNLEARRRLQFFTNSLFMDV
PPPKSVDKIVFTPYYSEVVLYSMAELTKRNEDGISILFYLQKIYPDEWKNFLARIGQDENALEGDLRNERDILELRF
WASYRGQTLARTVRGMMYYRKALMLQSYLERKAGRDGGESTLFGNNMTDAEGFELSPKARAQADLKFTYVV
TCQIYGRQKEDKKPEAVDIALLMQRNEALRIAYIDVVDTPKDGISHTEYYSKLVKADINGKDKEIYSIRLPGDPKLG
EGKPENQNHAIVFTRGNALQTIDMNQDNYFEEALKMRNLLEEFNRDHGIRPPTILGVREHVFTGSVSSLASFM
SNQETSFVTLGQRVLAKPLKIRMHYGHPDVFDRVFHITRGGISKASRVINISEDIFAGFNSTLRQGNITHHEYIQV
GKGRDVGLNQIALFEGKVAGGNGEQVLSRDVYRLGQLLDFFRMLSFYFTTVGFYFSTMLTVLTLYIFLYGRAYLAL
SGVGDTLRERAISLDDTALSAALNAQFLFQIGVFTAVPMILGFILEQGFLKAIVSFTTMQFQLCTVFFTFSLGTRTH

YFGRTILHGGARYQATGRGFVVKHITFSENYRLYSRSHFVKGLEVILLLVVYLAYGNDKAGAVSYILLTVSSWFLAG
SWLFAPFLFNPAGFEWQKVVVDFTEWTNWLFYRGGIGVKGSESWEAWWEEELSHIRTLSGRIMETILSLRFFV
FQYGIVYKLKLQGSDTSFSIYGWSWAALAIFFFLFKVFTFSRKISVSFQLVLRLAQGITFLVALAVVTVGVVLTDLSV
TDIFATVLALIPTGWGILSIACAWKPIIKRIGMWKSIRSLARLYDAGMGMLIFLPVAFCSWFPFLSTFQTRMMFN
QAFSRGLEISLILAGNHPNSDL

>Bra015504
MASTSGGRGEDGRPPQMQPVRSLSRRMTRAGTMMIDHPNEDENVIDSELVPSSLASIAPVLRVANEIEDQNP
RVAYLCRFHAFEKAHRMDPTSSGRGVRQFKTYLLHKLEKEEPTSDPKEIQNYYQRFYEENIQKGEGRKTPEEMA
KLYQMASVLYDVLKTVVPPARIDEKTHRYAKEVERKKDHYEHYNILPLDAGGAKTAIMELPEIKVAVRAVSNVEN
LPRPKLHSSSTNPDQVEREETRSFNDILEWLALVFGFQRGNVANQREHLILLLANVDVRKRDLENYDELKPSTVR
KLMDKYFKNYRSWCKYLRCESYLRFPPGCNEQQLSLVYIGLYLLIWGEASNIRFMPECFCYIFHKMADEVHGILF
SNVYPVTGDTYEAGAPDDEAFLRNVITPIYQVLRKEVRRNKMGKASHSKWRNYDDLNEYFWDKRCFRLGWP
MKPEADFFIHSDEIAQHPNERRDQVPHGKRKPKTNFVEARTFWNLYRSFDRMWMFLVLSLQTMMIVAWSPS
GSILAIFEEDVFFNVLTIFITSAFLNLLQATLDVILSFGAWKSLKFSQILRFITKFLMAAMWAIILPIAYSKSVQNPTGL
IKFFSSWVQSWPHQTLYNYAIALYVSPNILAAVFFLLPPLRRIMERSNMRIVTLIMWWAQPKLYVGRGMHEEM
FALFKYTFFWVMLLLSKLAFSFYVEILPLVKPTKLIWDMSGVNYQWHEFFPNATHNIGVIISIWGPIVLVYFMDT
QIWYAIFSTIFGGIYGAFSHLGEIRTLGMLRSRFRFVPSAFCSKLTPSPPGRAKRKHLDEQVDENDIARFSQMWN
KFIYTMRDEDLISDRERDLLLVPSSSGDVTVVQWPPFLLASKIPIALDMAKDFKGKEDAELFKKIKSEYYMYYAVV
EAYESMRDVIYGLLEDESDKRIVREICFEIDDSIQQHRFLSAFRMTGMPLLSDKLEKFLKILLSDYGEDETYKSQIIN
VLQDIIEIITQDVMVKGHEILERAHYQSGDIENEKKEQRFEKINLGGQNDSWREKVVRLLLLVTVKESAINIPQSLE
ARRRMTFFANSLFMNMPDAPRVRDMLSFSVLTPYYKEDVLYSEEELNKENEDGISILFYLQRIYPEEWSNFSERV
NDPKRIFSEKDKTDQLREWVSYRGQTLSRTVRGMMYYRMALELQCFQEYTEYAAHSGYLPSASYDEFMNRAR
ALADLKFTYVVSCQVYGNQKKSSDGRDRSCYNNILQLMLKYPSLRVAYIDEREETINKKSQKVFYSVLLKGCNKL
DEEIYRIKLPGNPTEIGEGKPENQNHAIIFTRGEALQTIDMNQDNYFEETFKMRNVLQEFDEGRRGKRNPTILGL
REHIFTGSVSSLAWFMSNQETSFVTIGQRVLANPLRVRFHYGHPDIFDRIFHITRGGISKASKIINLSEDIFAGYNS
TLRGGYITHHEYIQAGKGRDVGMNQVSVFEAKVANGNGEQTLSRDVYRLGRRFDFYRMLSFYFTTVGFYFSSM
ITVLTVYVFLYGRLYLVLSGLEKEILQSAIVHQSKALEEALAAQSVFQLGFLMVLPMVMEIGLEKGFRKALGDFIIM
QLQLASVFFTFQLGTKAHYFGRTILHGGSKYRATGRGFVVFHAKFADNYRLYSRSHFVKGLELVMLLIVYQVYGN
SYRSSSLYIYITFSMWFLVTSWLFAPFIFNPSGFEWQKTVDDWTDWKRWMGNRGGIGILVEKSWESWWESE
QEHLKHANLRGRVLEILLALRFLLYQYGIVYHLNVAHRDTTILVYGLSWGVLLAVLLVLKMVSMGRRKFGTDFQV
MFRILKALLFLGFLSVMTVLFVVCGLTIADVCASFLAFLPTGWAILLIGQALRGVLKGLGFWDSIKELGRAYEYIM
GLSIFTPIAVLSWFPFVSEFQTRLLFNQAFSRGLQISMILAGKKDKETPAPSK

>Bra023386
MSASRGGPDQGPSQPQQRRIVRTQTAGNLGESFDSEVVPSSLVEIAPILRVANEVESSNPRVAYLCRFYAFEKAH
RLDPTSSGRGVRQFKTALLQRLEREHDPTLMGRVKKSDAREMQSFYQHYYKKYIQALQNAADKADRAQLTKAY
QTANVLFEVLKAVNLTQSIEVDREILEAQDKVAEKTQLYVHYNILPLDPDSANQAIMRYPEIQAAVLGLRNTRGLP
WPEGHKKKKDEDMLDWLQEMFGFQKDNVANQREHLILLLANVHIRQFPKPDQQPKLDDQALTDVMKKLFK
NYKKWCKYLGRKSSLWLPTIQQEMQQRKLLYMALYLLIWGEAANLRFMPECLCYIYHHMAFELYGMLAGNVS
PMTGENVKPAYGGEEDAFLRKVVTPIYEVIAMESQRSKKGKSKHSQWRNYDDLNEYFWSVDCFRLGWPMRA
DADFFYPHVDQPNTEKDGDNSKPAVARDRWVGKVNFVEIRSFWHVFRSFDRMWSFYILCLQAMIIMAWDG
GQPSSVFGADVIKKVLSVFITAAIMKLGQASLDVILSFKAHRSMSLHVKLRYILKVLSAAAWVIILPVTYAYSWEDP
PGFARTIKSWFGSAMHSPSLFIIAVVFYLAPNMLAGVFFLFPMLRRFLERSNYRIVMLMMWWSQPRLYVGRG

MHESAFSLFKYTMFWVLLIATKLTFSYYIEIKPLVAPTQAIMRARVTDFQWHEFFPRAKNNIGVVIALWAPIILVYF
MDSQIWYAIFSTIFGGIYGAFRRLGEIRTLGMLRSRFESLPGAFNDRLIPDGKNQQRKKGLRATLSHNFTEDKVP
VNKEKEAARFAQLWNTIISSFREEDLISDREMDLLLVPYWADRDLDLIQWPPFLLASKIPIALDMAKDSNGKDRE
LKKRIESDSYMKCAVRECYASFKNVIKFLVQGNREKEVIEFIFAEVDEHIEKGDLIQVYKMSCLPSLYDHFVKLIKYL
LDNNVDDRDHVVILFQDMLEVVTRDIMMEDSISSLVDSSHGGTWHGGMVPLEQQYQLFASSGAIRFPIEPVT
EAWKEKIKRLYLLLTTKESAMDVPSNLEARRRISFFSNSLFMDMPAAPKVRNMLSFSVLTPYYTEEVLFSMHDLD
TPNEDGVSILFYLQKIFPDEWNNFLERVKSNEEEIKESVELEEELRLWASYRGQTLTRTVRGMMYYRKALELQAF
LDMAMHEDLMEGYKAVELNSENNSRGERSLWAQCQAVADMKFTYVVSCQQYGIHKRSGDPRAQDILRLMT
RYPSLRVAYIDEVEEPVKDKSKKGNEKVYYSVLVKVPKSTDHSSLAQNLDQVIYRIKLPGPAILGEGKPENQNHAII
FSRGEGLQTIDMNQDNYMEEALKMRNLLQEFLTKHDGVRHPSILGLREHIFTGSVSSLAWFMSNQETSFVTIG
QRLLANPLRVRFHYGHPDVFDRLFHLTRGGVSKASKVINLSEDIFAGFNSTLREGNVTHHEYIQVGKGRDVGLN
QISMFEAKIANGNGEQTLSRDIYRLGHRFDFFRMMSCYFTTVGFYFSTLITVLTVYIFLYGRLYLVLSGLEQGLSTQ
KGIRDNTPLQIALASQSFVQIGFLMALPMLMEIGLERGFRTALSEFVLMQLQLAPVFFTFSLGTKTHYYGRTLLH
GGAKYRSTGRGFVVFHAKFADNYRLYSRSHFVKGLEMMLLLVVYQIFGSAYRGVVAYLLITISMWFMVGTWLF
APFLFNPSGFEWQKIVDDWTDWNKWINNIGGIGVPAEKSWESWWEEEQEHLRHSGKRGIVVEILLSLRFFIYQ
YGLVYHLTITEKTKNFLVYGVSWLVIFLILFVMKTVSVGRRKFSASFQLMFRLIKGLIFLTFIAIIVILITLAHMTIQDIIV
CILAFMPTGWGMLLIAQACKPVVHRAGFWGSVRTLARGYEIVMGLLLFTPVAFLAWFPFVSEFQTRMLFNQA
FSRGLQISRILGGHRKDRSSRNKE

>Bra027328
MSHEIVPVDPIDIPSTSYSRPAQGREDSPEREHQYTRSLTFREHVSEPFDSERLPATLASEIQRFLRIANLVESEEPRI
AYLCRFHAFEIAHHMDRNSTGRGDEEFTVIRRKEKSDVRELKRVYHAYKEYIIKHGATFNLDHSQREKLVNARRI
ASVLYEVLKTVTSGAGPQAIADRESIRAKSEFYVPYNILPLDRGGVHQAIMHLPEIKAAVAIVRNTRGLPPPEDFQ
RHQPFVDLFEFLQYAFGFQSGNVANQREHMILLLSNTIIRQPQKQSSQPKSGDEAVDALMKKFFKNYTSWCKF
LGRKNNIRLPCVKKEALQYKTLYIGLYLLIWGEASNLRFMPECLCYIFHQMAYELHGVLAGDVSMITGEKVVPAYR
GVSHETFLEKVVTPIYKVIEKEAEKNKNGTADHSMWRNYDDLNEFFWSIECFELGWPMRSEHDFFCVEPLDTS
KPRRWREKLRFRRQTKKTDEEMEDDEELGPITEEQIKPTQRWLGKTNFVEIRSFWQIFRSFDRMWSFFVLSLQ
ALIIMACHDVGSPLQIFNANIFEDVMSIFITSAFLKLIKGILDIIFKWKTRTTMPINEKKKQMARLGLAAMWTVILP
VLYSHSRRKYICYFTSYKTRLGEWCFSPYMVAVTIYLTGSAVELVLFFVPAISKYIETSNHRVFKTLYWWGQPRLFIG
RGVQETQISQFKYTFFWILVLLTKFAFSYAFEIKPLIEPTRLIMKVGVRNYEWHEIFPEVKSNAAAIVAVWAPIMV
VYFMDTQIWYSVFCTIFGGLYGVLHHLGEIRTLGMLRGRFHTLPSAFNASLIPHSMKDERKRKERGFFPFNFCRG
SDGQKNSMAKFVLVWNQVINSFRIEDLISNKELDLMTMPMSSEVLSGIIRWPIFLLANKFSTALSIAKDFKEKDE
ALYRRIRRDEYMYYAVKECYESLKYILQILVVGDLEKKIISGIINEIEESIRQSSLLEEFKLKELPTLHKKCIELVQLLVEG
SEDKLPVEKIEEQHSKLVKALQDIFELVTNDMMVHGDRILDLLEPLEDSEEDTGIFMRVIEPQLFESYGERRCIHF
PLPDSTSLSEQIQRFLLLLTVKDSAMDIPENLDARRRISFFATSLFMDMPDAPKVRNMMSFSVLTPHYQEDINFS
TKELHSAKSSVSIIFYMQKIFPDEWKNFLERMGCENLEALKREGKEEELRKWASFRGQTLSRTVRGMMYCREAL
KLQAFLDMADDEDILEGYDDVERSNRPLAAQLDALADMKFTYVVSCQMFGAQKSAGDPHAQDILDLMIKYPS
LRVAYVEEREEIVSDNPEKVYYSILVKAVNGFDQEIYRVKLPGPPNIGEGKPENQNHAIIFTRGEALQTIDMNQD
NYLEEAFKMRNLLQEFLRNRGRRPPTILGLREHIFTGSVSSLAWFMSYQETSFVTIGQRLLANPLRVRFHYGHPD
VFDRIFHITRGGISKASRTINLSEDVFAGYNTTLRRGCITYNEYLQVGKGRDVGLNQISKFEAKVANGNSEQTISR
DIYRLGQRFDFFRMLSCYFTTIGFYFSSLISVIGIYIYLYGQLYLVLSGLQKTLILEAKVKNIKSLETALASQSFLQLGLLT
GLPMVMEIGLEKGFLIAFQDFILMQLQLAAFFFTFSLGTKTHYFGRTILHGGAKYRPTGRKVVVFHANFSENYRL
YSRSHFIKGFELIILLVVYELFKHTSQSNMAYSFITFSVWFMSLTWLCAPFLFNPSGFTWEIIVGDWRDWNRWIK
EQGGIGIQQDKSWQSWWNDEQAHLRGSGVGARCLEIVLSLRFFLYQYGLVYHLDITQSSTNIIVYALSWVVILAT

FLTVKAVDLGRQLFSTRKHLVYRFFKVFVFVSILTVIITLSNICHLSIKDLIVSCLAFLPTGWGLILIAQAVRPKIEGTSL
WEFTQVLARAYDYGMGVVLFAPMAILAWLPIISAFQTRFLFNEAFNRRLQIQPILAGKKKK

>Bra040364
MSRVEALWERLVNAALRRDRTGESAGGGCPGEGRDIDDILRAADELQADDPVIARILCEHAYSLAQKLDPNSES
VSVLQFRTGLMSVIKQRDEKREVETINRSQDINRLQGFYQRYREKNNVDTLKEEEKQLCESGAFTDELEQKTVER
KRVFANLKVLEHVLEQVSKEIFEELKHANDSDAAMSHEDTVAYNIVPFDAPVTANATTAFCEVQAAVTALKYFPG
LPKLPAEFPLPATRNADMLDFLHYIFGFQRDSVSNQREHIILLLSNEQSRLNIPGETEHKLDDAAVSNVFLKSLDN
YIKWCDYLCIQPSWRNLEAISGEKKLLYLSLYFLIWGEAANIRFLPECLCYIFHHMVREMDEILRQQVARPAESCR
PVDSCGSEDDVSFLDHVISPLYEVVSAEAFNNGNGRVPHSAWRNYDDFNEYFWSLQSFELGWPWRTSSSFFQ
KPRPRAKFELKPGREKHRGKTSFVEHRTFLHLYHSFHRLWIFLAMTFQALAIVAFNEKSLASRKTLREILSLGPTYV
VMKFSKSVLDVFMMYGAYSTTRRLAVYRMFLRLIWFGLASVFISFLYVRALQEDSKPNSDSVMFTLYVIVIAIYG
GVQFFFGVLMRIPACHNIANKCDGWTVVRFFKWMWRQERHYVGRGMYEKTSDFIKYLLFWLVVLSAKFSFAY
FLQIEPLVSPTRMIVKHGNIPYYWHDFVSRSYKYEIGPSGYVISSTIIGNYNALTVASLWAPVASIYLLDIHIFYTLVS
AFLGFFLGVRDRLGKGPYVALPFSQIRSLEEIHKQFKKFPGAFMRALHVPITNRFFFSYLHVFINSKVVDKKNRVE
AAHFAPFWNQIIKCLREEDYITDFEMDLLLMPKNYGSRLVQWPLFLLSTKILLATEIAAESKSQEEIVKRIEKDAYM
KYAVEEVYYSLERVLITTLEAEGKIWVDRIFRDIRTSITMRTIHLDFTLKKLSLVITRVTALLGVLKENETPENAAAVT
KALQDLYDVMRLDILAVDMRGHYDAWNVITRASNEGRLFTKLKWPKDPEMKALVKRLYSLLTIKDSTAPHVPR
NLEARRRLQFFTNSLFMDVPQPKPVHQMLSFSVFTPYCSEVVLYSMAELTKRNEDGISILFYLQKIYPDEWKNFL
ARIGKDENALEGDLHNERDILELRFWASYRGQTLARTVRGMMYYRKALMLQSYLERKAGTVTDEESTLYGNNP
TDAEGFELSPEARAQADLKFTYVVTCQMYGRQKEDQKPEAADIALLMQRNEALRIAYIDVVDTLKEVKYHKEYY
SKLVKADIDGKDKEIYSIRLPGDPKLGQGKAENLNHAIVFTRGNAVQTIDVNQDNYFEEALKMRNLLEEFDRDH
GIRPPTILGVREHVFTGSVSSLASFMSNQETTFVTLGQRVLAKPLKIRMNYGHSDVFDRVFHITRGGISKASRVIN
ASEDIFAGLNSTLRQGNITHHEYIQVGKGRDVGLNQIALFEGKVAGGNGEQVLSRDVYRLGQLLDFFRMMSFY
FTTVGFYFCTMLTVLTLYIFLYGKAYLAFSRVGATIRERAILVNNTAHSAALSVQFLLQIGVFTAVPMILGFILEHGFL
QAIVSFTAMQFQLCTVFFTFSLGTRAHYFGRTLLHGGASVCQQYQATRRGFVLKHIKFSENYRLYSRSHFVKGM
EVILLLVVYLAYGNDEAGSVSYILLTVSSCFLAFSWLFAPYMFNPSGFERQKVVEDFKEWTKWLFYRGGGIGVEG
AESWEAWWEEELSHIGTLSGRMVETILSLRFFIFQYGIVYKLNVHGSDTSFAVYGWSWAAFAVILVIFKVFAFIQK
IAVSFRLVRRFIQGLALLVSLAGIIVAVVLTELSVQDIFASVLAFLPTGWGILSIACAWKPPIKRIGMWESVRSLARLY
DAGMGMLIFLASAFLSLFPFVSTFKHV

>Bra003364
MTITRTLFESKTDKCYAPSFVAVNLCLALIDGALAFIAFLQLSRFHRRDKRVGWTRQKVLHLMIGSSNTAFPKILFL
ATFLLLLSFWVDVCHQGNGEEEDDDDEENSVQQVLLEKAKSKPGSSSASDRRKCCSFHGIHVGTRQKFVVAAV
VLVFILMISFAILIWIASGDNSADPSLLAEVYVDIFASTLLITGGGLCFYGMRLFFNLRKVRSEQVSSEMRKVSGLA
GVSVVCFTVSSLIALLTHIPLFYHWNPNKLHGINALVLLIIYYFIGSTLPLAFVLWVLRELPPQNMVSRQEEQTRITY
VNYDAVPRQPPQQWTSTTVSKNQVSEFELFGEEGVTDCSYSRLAMEASSSGTAELPRSLSRRAAPSRATTMMI
DRPNEDASAMDSELVPSSLASIAPILRVANEIEKDNPRVAYLCRFHAFEKAHRMDDTSSGRGVRQFKTYLLHRLE
KEEEETKPQLAKSDPREIQAYYQNFYEKYIKEGETSRKPEEMARLYQIASVLYDVLKTVVPSPKVDYETRRYAEEVE
RKRDRYEHYNILPLNAVGTKPAIVELPEVKAAFSAVRNVRNLPKRRVHMPSNAPDEMRKARSRRFNDILEWLA
SEFGFQRGSVANQREHIILLLANADIRNRTDEEYDGLKSSTVTELMDKTFKNYYSWCKYLHREPNLKFPPDCDV
QQLQLIYISLYLLIWGEASNVRFMPECICYIFHHMANDVYGILFSNVKAVSGEAYETGEIIDEETFLRNVITPIYQVI
RNEARRNKGGTASHSQWRNYDDLNEYFWSRKCFKIGWPLDPNADFFQNSDESTPQNERLNQVTYGKRKPKT
NFVEVRTFWNLFRDFDRMWIFFVMAFQAMVIVGWNGSGSLGEIFDKDIFKKVLTVFITSAYLTLLQTALDIILTF

NAWKNFKLSQILRYLLKLAVAAMWAVLLPIAYAKSVQRPSGVVKFFSTWTGDWKDKSFYNYAVSFYVLPNILAA
FLFLVPPFRRVMECSDMRPKLYVGRGMHEDMLSLFKYTSFWIMLLISKLSFNYYVEILPLIKPTKMIMNLHIRNY
QWHEFFPYANNNVGVVIAVWAPIVLVYLMDAQIWYAIFSTLFGGIHGAFSHLGEIRTLGMLRSRFESIPIAFSRTL
MPTENANRKHADDFGDQKKITNFSQVWNEFIISMRREDKISDRDRDLLLVPSSSGDVSVIQWPPFLLASKIPIA
VKMAKDFKGKEDAELFRKVTSDSYMHYAVTESYETLKKIISALLEDEADRRVMNQVFSEVDMSIQKQRFIYDFR
MSGLTLLSDKLEKFLRILLSDYEDEGTYKSQLINVFQDVIEIITQDLLVNGHEIVERARIHSPDVKNEKKEQRFEKINI
HLIQDKRWRDKVVRLHLLLSVKESAINVPQNLEARRRITFFANSLFMNMPNAPRIRDMLSFSVLTPYYKEDVLYS
EEELNKENEDGISILFYLQKIYPDEWTNFLDRLNDPKLLEKDKSEFLREWVSYRGQTLARTVRGMMYYRQALEL
QCYQEVAGENAKFSVHQAMASNDEHQKAFLERAKALADLKFTYVVSCQVYGNQKKSGDIHNRSCYTNILQLM
LKYPSLRVAYVDEREETADAKSPKVFYSVLLKGGAKFDEEIYRIKLPGPPAEIGEGKPENQNHAIIFTRGEALQTID
MNQDNYFEEAFKLRNVLEEFKKERVGRRKPTILGLREHIFTGSVSSLAWFMSNQESSFVTIGQRILANPLRVRFH
YGHPDIFDRIFHITRGGVSKASKVINLSEDIFGGFNSTLRGGYVTHHEYIQVGKGRDVGLNPISIFEAKVANGNGE
QTLSRDVYRLGHRFDFYRMLSFYFTTIGFYFSSMLTVITVYAFLYGRMYMVMSGAEKEILRLATPNQLAALEQAL
ATQSIFQLGFLMVLPMVMEIGLEEGFRSAIVDFFIMQLQLASVFFTFQLGTKSHYYGRTILHGGSKYRPTGRGFV
VFHAKFAENYRLYSRSHFVKGLELLLLLIVYQVYGHSYRSSNLYLYITVSMWFMVGSWLFAPFIFNPSGFEWQKT
VDDWTDWKRWLDDRGGIGIPVDKSWESWWSVEQEYLKHTNIRGRILEITLALRFFIYQYGIVYQLNISQNSKSF
LVYGLSWVVLLTSLLVLKMVSMGRRKFGTDFQLMFRILKALLFLGFLSVMTVLFVVCELTLTDLSASILAFLPTGW
AILLIGQVLRSPIKALGIWDSVKELGRAYEKIMGLVIFAPIAVLSWFPIVSEFQARLLFNQAFSRGLQISMILAGRKD
KAASSYK

>Bra029541
MSMRPRPSPATAHAPSQEVYNIIPIHDFLTEHPSLRYPEVRAAAAALKVVGDLAKPPFVDFTPRMDLMDWLGL
LFGFQLDNVRNQRENLVLHLANSQMRLQPPPLHPDGLDPTVLRRFRKKLLRNYTNWCSFLGVRCHVTSPAHSR
HQTNVVLNLRRELLYVALYLLIWGESANLRFMPECICYIFHHMAMELNKVLGGEFDDMTGMPYWPSFSGDCA
YLKSVVMPIYRTVKTEVESSNNGTKPHSAWRNYDDINEYFWRKRALKSLKWPLDCTSNFFDTTPKSSRVGKTGF
VEQRSFWNVFRSFDRLWILLLLYLQAAIIVATSRVKYPWQDKDVEVALLTVFITWAGLRLLQSVLDASTQYSLVGR
ETFWLFVRLILKVLVAVTWTVLFSVFYARIWSQKNKDGLWSQAANDRIIVFLKVVFVYVIPEMLALVLFIVPCIRN
WVEELNLGIVYFFTWWFYSKSFVGRGLREGLVDNVKYSIFWIVVLATKFIFSYFLQIRPLIKPTRALLNLKNAPYN
WHEFFGSTHRIAVVMLWLPVILVYLMDLQIWYSIYSSLVGATIGLFSHLGEIRNIDQLRLRFQFFSSAMQFNLKPE
ERLLGPKATVLKKARDAIHRLKLRYGIGQPFNKIESSQVEATWFALLWNEIILTFREEDLISDREVELLELPPNCWNI
QVIRWPCFLLCNELLLALSQANELCDAPDRWLWSKICSSEYRRCAVIEAFDSIKFVIRKIVKNGTEEESIVNRLLNEI
DENVESRKVTEVYKLTVLLRIHEKLISLLERLMDPDKKVFRIVNILQALYELCAWEFPRIRRSTQQLRQLGLAPVSL
NADTELLFVNAINLPPPGDVVFYRQIRRVHTILTSRDPMHNVPKNLEARERLAFFSNSLFMNMPQAPSVEKML
AFSVLTPYYDEEVMYRQEMLRAENEDGISTLFYLQKIYADEWVNFVERMRREGVENENDIWTQKVRDLRLWA
SYRGQTLSRTVRGMMYYYSALKKLAFLDSASEMDISMGTQIAPEPPRSYYTRDGGDNILQPTASQEISRMANGI
GHLFKGSESGSAMMKFTYVVACQVYGQHKAKGDHRAEEILFLMKSHEALRIAYVDEVNLGLGNVEYYSVLVKF
DQRLQREVEIYRIRLPGPLKLGEGKPENQNHAIIFTRGDAIQTIDMNQDNHFEEALKMRNLLESFKKNYGIRKPT
ILGVREKVFTGSVSSLAWFMSSQETSFVTLGQRVLANPLKVRMHYGHPDVFDRFWFIPRGGISKASRVINISEDI
FAGFNCTLRGGNVTHHEYIQVGKGRDVGLNQISMFEAKVASGNGEQALSRDVYRLGHRLDFFRMLSFFYTTV
GYYFNTMLIVFTVYAFLWGRLYLALSGVEKIAKDRSSSNEALGAILNQQFVIQLGLFTALPMILENSLERGFLPAIW
DFITMQLQLASFFYTFSLGTRSHYFGRTILHGGAKYRATGRGFVVEHKKFAENYRLYARTHFIKAIELAIILLVYAAYS
PLAKSSLVYILMTISSWFLITSWIISPFLFNPSGFDWLKTVYDFDDFMNWLWSRGGLFTKADQSWFTWWNEE
QDHLKTTGVWGKLLEILLDLRFFFFQYSIVYHLRIADGQTSIGVYLVSWGCIIGIAAIYITTIYAQKRFSVKEHIKYRFI
QFLVIWLTVLVVVLMLQFTKLTVVDLLISLLAFIPTGWGLISIAQVLRPFLISTVVWDTVISVARLYDLCFGLIVMAP

VALLSWLPGFQNMQTRILFNEAFSRGLQISIILAGKKSA

>Bra005239
MARVYSNWDRLVRATLRREQLRDSGQGHERVNSGLAGAVPPSLGRATNIDAILQAADEIQAEDPNVARILCEQ
AYSMAQNLDPNSDGRGVLQFKTGLMSVIKQKLAKRDGASINRDRDIERLWQFYQLYKRRHRVDDIQREEQKW
RESGTAFSSNVGEILKMRKVFATLRALVEVLEVLSRDADPNGVGRSIREELGRIKKADATLSAELTPYNIVPLEAQS
MTNAISVFPEVRGAIQAIRYTEHFPKLPDDYEISGQRDADMFDLLEYIFGFQKDNVRNQREHLVLTLSNAQSQL
GLPSPNDPKIDEKAVNEVFLKVLDNYIKWCKYLRIRLVYNKLEAINRDRKLFLVSLYFLIWGEAANVRFLPECICYIF
HNMAKELDAKLDHGEAVRADSCVIENGSVSFLDRVISPIYAAMSAETLRNNNGKAAHSEWRNYDDFNEYFWT
PGCFELSWPMKTESKFLTGPKGRKRTGKSSFVEHRTYLHLFRSFHRLWIFMVIMFQALAIIAFRKEHLDKDTFKIL
LSAGATYAIMNFIESFLDVVLMYGAYSMARGMAISRVFIKFFWWGLGSVFVVYVYVQVLQERNKRTSDEFFYRL
YILVLGSYAAVRLIFGLLVKLPACHALSEMSDQSFFQFFKWIYQERYFVGRGLFENISDYCRYVAFWLIVLASKFTFA
YFLQARMFLQLYIFATIKPLVKPTKTIIDLPSFEYSWHDIVSKSNDHALTIVSLWAPVVCIYLMDIHIWYTLLSAIIGG
VMGAKARLGEIRSIEMVHKRFESFPEAFAKNLVSPVVKRVSFGQHTSQDGQDMNKAYAAMFSPFWNEIIKSLR
EEDYISNREMDLLSIPSNTGSLGLVQWPLFLLCSKILVAIDLAMECTETQGVLWRQICDDEYMAYAVQECYYSVQ
NILNSMVDGVGRRWVERVFMEISNSIQEGSLAITLNLKKLQLVVSRFTALTGLLIRNETPALAKGAAKAMFDFYE
VVTHDLLAENLRDQLDTWNILARARNEGSLFSNIEWPRDPEIIEQVKRLHLLLTVKDAAANVPKNLEARRRLEFF
TNSLFMDMPQAKPVAEMVPFSVFTPYYSETVIYSSSELRSENEDGISTLFYLQKIFPDEWENFLERIGRSDSTGDA
DLQESATDALELRFWVSFRGQTLARTVRGMMYYRRALMLQCFLERRGLGVDDFSLTNMPRGFEASPEARAQA
DLKFTYVVSCQIYGQQKQQKKPEATDIALLLQRFEALRVAFIHSEDVGVEGKKEFYSKLVKADIHGKDQEIYSIKLP
GDPKLGEGKPENQNHAIVFTRGEAIQTIDMNQDNYLEEAIKMRNLLEEFHGKHGIRRPTILGVREHVFTGSVSS
LAWFMSNQETSFVTLGQRVLAYPLKVRMHYGHPDVFDRVFHITRGGISKASRVINISEDIYAGFNSTLRQGNIT
HHEYIQVGKGRDVGLNQIALFEGKVAGGNGEQVLSRDVYRIGQLFDFFRMMSFYFTTVGFYVCTMMTVLTVY
VFLYGRVYLAFSGSDRAISRVAKLSGNTALDAALNAQFLVQIGVFTAVPMVMGFILELGLLKAIFSFITMQFQLCS
VFFTFSLGTRTHYFGRTILHGGAKYRATGRGFVVQHIKFADNYRLYSRSHFVKAFEVALLLIVYIAYGYTDGGAVSF
VLLTISSWFLVISWLFAPYIFNPSGFEWQKTVEDFDNWVSWLMYKGGVGVKGELSWESWWEEEQMHIQTLR
GRILETILSLRFFMFQYGVVYKLNLTAKDTSLALYGYSWIVLVAVVLLFKLFWYSPRKSSNILLALRFLQGVVSLVVIA
LIALAIALTDLSIPDMFACVLGFIPTGWAILSLAITWKRLIKLFGLWETVREFGRIYDAAMGMLIFAPIALLSWFPFI
STFQSRLLFNQAFSRGLEISIILAGNRANVET

>Bra015436
MSQRRESGPSRPHRPIQRTQTLGSLGEAMLDSEVVPSSLVEIAPILRVANEVEASNPRVAYLCRFYAFEKAHRLD
PTSSGRGVRQFKTALLQRLERENETTLAGRQKSDAREMQRFYQHYYEKYIHALNAADKADRAQLTKAYQTAAV
LFEVLKAVNQTEDVPVPIKILQQQKKVEEKTQIYKPYNILPLDPDSQNQAIMRLPEIQAAVTALRNIRGLPWKAG
HKKKIDEDILDWLQSMFGFQEDSVSNQREHLILLLANVHIRQYPRPEQEPKLDDRALTIVMKKLFRNYKKWCKY
LGRKSSLWLPTIQQEVQQRKLLYMGLYLLIWGEAANLRFMPECLCYIYHHMAFELYGMLAGSVSSMTGEHVKP
AYGGDDEAFLQKVVTPIYKTIAKEAKRSRNGKSKHSVWRNYDDLNEYFWSIRCFRLGWPMRADADFFCLTAEE
LRVENSEIKSNSGDRWMGKVNFVEIRSFWHIFRSFDRMWSFYILCLQAMIVIAWNGSGELSAIFQGDVFLKVLS
IFITAAVLKLAQALLDIALSWKARHSMSPYVKLRYVLKAGAAAGWVIVMPVAYAYSWKNASGFALTIKNWFGG
HSHNSPSLFIVAILIYLSPNMLSALLFLFPFIRRYLERSDFKIMMLMMWWSQPRLYIGRGMHESALSLFKYTMF
WIVLLISKLAFSFYAEIKPLVGPTKDIMRIHISVYSWHEFFPHAKNNLGVVIALWSPVILVYFMDTQIWYAIVSTLV
GGLNGAFRRLGEIRTLAMLRSRFQSIPGAFNDCLVPHEQSDDTKKRGFKATFSRKFDQLPSSKDKEAARFAQM
WNKIISSFREEDLISDREMELLLVPYWSDPDLDLIRWPPFLLASKIPIALDMAKDSNGKDRELKKRLAVDSYMTC
AVSECYASFKNLINYLVIGERERQVINDIFSKIDQHIEKETLITELNLSSLPDLYGQFVQLIEYLIQNREEDKDQIVIVL

LNMLEVVTRDIMDEEVPSLLETAHNGAYVKYDVMTPLHQQRKYFSQLQFPIKRLHLLLTVKESAMDVPSNLEAR
RRLTFFSNSLFMDMPPAPKIRNMLSFSVLTPYFSEDVLFSIFGLEQQNEDGVSILFYLQKIFPDEWTNFLERVKCG
SEEELRTKDELEEELRLWASYRGQTLTKTVRGMMYYRKALELQAFLDMAKDEELLKGYKALELTSEEASKSGESL
WAQCQALADMKFTFVVSCQQYSIHKRSGDQRAKDILRLMTTYPSIRVAYIDEVEQTHKESYKGTEEKIYYSALVK
AAPQTKPMDSSESVQTLDQLIYRIKLPGPAILGEGKPENQNHAIIFTRGEGLQTIDMNQDNYMEEAFKMRNLL
QEFLVKHGGVRFPTILGLREHIFTGSVSSLAWFMSNQENSFVTIGQRVLASPLKIRFHYGHPDIFDRLFHLTRGGI
CKASKVINLSEDIFAGFNSTLREGNVTHHEYIQVGKGRDVGLNQISMFEAKIANGNGEQTLSRDLYRLGHRFDFF
RMLSCYFTTIGFYFSTMLTVLTVYVFLYGRLYLVLSGLEQGLSNQRAFRNNRPLEAALASQSFVQIGFLMALPMM
MEIGLERGFHNALIEFVLMQLQLASVFFTFQLGTKTHYYGRTLFHGGAEYRGTGRGFVVFHAKFAENYRFYSRS
HFVKGIELMILLLVYQLFGQSYRGVVTYILITVSIWFMVVTWLFAPFLFNPSGFEWQKIVDDWTDWNKWIYNR
GGIGVPAEKSWESWWEKELEHLKHSGVRGIVLEIFLALRFFIFQYGLVYQLSIFKGKNQSFWVYGASWFVILFLLL
IVKGLGMGRRRFSTSFQLLFRIIKGLVFLAFVTILITLLALPLITIKDLFICMLAFMPTGWGMLLIAQACKPLIQHLGV
WSSVKTLARGYEIVMGLLLFTPVAFLAWFPFVSEFQTRMLFNQAFSRGLQISRILGGHRKDRSSKNKE
