# Supplementary material for: Genome-Wide Analysis of Callose Synthase (CALS) Genes in Cabbage (Brassica oleracea var. capitata L.): Identification and Expression Profiling During Hyaloperonospora parasitica Infection
Source: Int J Mol Sci. 2025 Oct 23;26(21):10304. doi: 10.3390/ijms262110304 (PMC12610245; doi:10.3390/ijms262110304)
Supplement: Supplementary file 1 [file ijms-26-10304-s001.zip › Supplementary Table S2. Primer senquence used for Quantitative Real-Time PCR.pdf]

Supplementary Table S2. Primer senquence used for Quantitative Real-Time PCR

Primer name	Forward primer sequence (5’-3’)	Reverse primer sequence (5’-3’)
qBoCALS2	CAGTGGCCTCCGTTCTTATT	CTTCTTGAGCTCTCTGTCCTTC
qBoCALS4	CGACACTTAGGCAGGGAAATA	CCGCTACCTTCCCTTCAAATA
qBoCALS5	CTGTGCGAGGCATGATGTATTA	CTCAAGAGCCTTGTACCCTTTC
qBoCALS6	CAAACTGCTTCCGTTCTCTTTG	GTCTTCTCTTCCACCTTCTTCTG
qBoCALS8	GATCTCCGTGATCGGGATTTAC	CTTCACCTTGGCTTCGAGAATA
qBoCALS9	CTGGTTCTTGGTAGTCTCTTGG	GAGCCAGTTTGTCCATTCTTTG
qBoCALS10	CTGGAGGAGGACTATGCTTCTA	CAACTGAGACGCCTGCTAAA
qBoCALS11	CACTGCGTTTCCTGCTTTAC	CGCCCAAGAGAGTCCATAAA
qBoCALS14	GAGTAGTAACAACGGGACGAAG	GGTACAGTCAAGAGGCCATTTA
qBoCALS15	TCCGAATGATGTCGTGTTACTT	GCCCACTCAAGACAAGATAGAG
